# Bidirectional Photoswitching of a Tailored Azobenzene with Red and Far‐Red Light Involving Triplet Sensitization in an Aqueous System

**DOI:** 10.1002/advs.75155

**Published:** 2026-04-23

**Authors:** Mila Miroshnichenko, Helen Hölzel, Edvinas Orentas, Karolis Kazlauskas, Pedro Ferreira, Fabienne Dumoulin, Carles Alcaide, Miquel Solà, Roger Bresolí‐Obach, Santi Nonell, Pankaj Bharmoria, Kasper Moth‐Poulsen

**Affiliations:** ^1^ The Institute of Materials Science of Barcelona ICMAB‐CSIC Barcelona Spain; ^2^ Department of Chemical Engineering Universitat Politècnica de Catalunya EEBE Barcelona Spain; ^3^ Institute of Organic Chemistry Justus‐Liebig‐University Giessen Giessen Germany; ^4^ Institute of Chemistry Faculty of Chemistry and Geosciences Vilnius University Vilnius Lithuania; ^5^ Institute of Photonics and Nanotechnology Faculty of Physics Vilnius University Vilnius Lithuania; ^6^ Acıbadem Mehmet Ali Aydınlar University Faculty of Engineering and Natural Sciences Biomedical Engineering Department Istanbul Türkiye; ^7^ Acibadem Mehmet Ali Aydinlar University Graduate School of Natural and Applied Sciences Istanbul Türkiye; ^8^ Institute of Computational Chemistry and Catalysis (IQCC) and Department of Chemistry Universitat de Girona Girona Spain; ^9^ Institut Químic de Sarrià, Universitat Ramon Llull Barcelona Spain; ^10^ Catalan Institution for Research & Advanced Studies ICREA Barcelona Spain; ^11^ Department of Chemistry and Chemical Engineering Chalmers University of Technology Gothenburg Sweden

**Keywords:** azobenzene, photoswitching, singlet‐oxygen, spectral shift, triplet‐sensitization

## Abstract

Shifting action spectrum of azobenzene‐based photopharmaceuticals toward bio‐optical window is highly desired, since red/far‐red light offers improved tissue penetration and reduced cellular toxicity. While unidirectional photoswitching (*trans‐to‐cis* and *cis‐to‐trans*) of azobenzenes (AZO) with red/far‐red light is known in separate systems, achieving bidirectional photoswitching in a single system faces issues of spectral overlaps. Here, bidirectional photoswitching of a tailored azobenzene is achieved in a single bio‐relevant solution with red and far‐red light excitation. A new azobenzene molecule (**AZO‐N**) bearing four *ortho*‐methoxy, and one *para*‐N donor lipophile is synthesized. The **AZO‐N** exhibits separate n‐π* absorption bands and triplet energies for *trans* and *cis* isomers. As a result, *trans*‐to‐*cis* photoswitching is observed upon 625 nm excitation at the tail of the red‐shifted n‐π*absorption band via direct absorption, whereas *cis*‐to‐*trans* photoswitching is enabled upon 730 nm excitation through triplet sensitization. Further, triplet sensitization of **AZO**‐based molecules could lead to singlet oxygen generation—experiments in the presence of serum albumin and glutathione (biological oxidative stress addressors), suggest no effect of singlet oxygen on the triplet‐sensitization of *cis*‐**AZO‐N,** and non‐significant change in the fingerprint all‐*α* secondary structure of serum albumin. This advance presents modular design principles to develop practically functional azo‐based photopharmaceuticals.

## Graphical abstract

The action spectrum of a tailored azobenzene is shifted toward bio‐optical window through the chemical modification of its singlet and triplet energy levels. Consequently, the bidirectional photo‐switching of azobenzene in physiological conditions is achieved when excited by red and far‐red light, which offers greater tissue penetration. This represents an important advancement for the potential application of azobenzene‐based photopharmaceuticals.

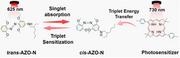

## 1 Introduction

Organic photoswitches are at the center of photo‐functional materials research, including photopharmaceuticals, as they leverage spatiotemporal control to molecules/drugs due to light‐induced geometrical changes [1, 2, 3, 4, 5, 6]. Azobenzenes, due to their superior photochemical stability, large geometrical change upon isomerization, and synthetic accessibility, are the most promising photoswitches in the field of photopharmaceuticals [7, 8, 9]. Azobenzene undergoes *cis*‐*trans* geometrical isomerization upon photoexcitation that provides spatial control, whereas the power dependence of their photo‐kinetics leverages temporal control. The unsubstituted *trans*‐azobenzene shows a strong π–π* absorption at 320 nm and a weak n–π* absorption at 440 nm, whereas the *cis*‐azobenzene shows n–π* absorption at 440 nm along with the π–π* absorptions at 280 and 250 nm [10, 11]. However, the practical in vivo photopharmacology demands photopharmaceuticals, to work under excitation with tissue penetrating red to near‐infrared light (the bio‐optical window) [12, 13], due to low skin toxicity compared to UV‐light [14, 15]. As a result, several strategies have been developed to red‐shift the action spectrum of azobenzene toward the bio‐optical window [16, 17]. These strategies include chemical engineering with electron‐rich/poor or pull‐push substituents at *ortho*, *para* or *meta* position [16, 17, 18, 19, 20, 21], 2‐ or 3‐photon absorption [22], and recently, triplet sensitization [20, 23, 24]. Woolley and co‐workers introduced the *ortho*‐substitution strategy with electron‐donating groups (amino, o‐methoxy, chloro, thioether), which allowed *trans*‐to‐*cis* isomerization upon irradiation with >400 nm light (530–625 nm), separation of n‐π* absorption bands of *trans* and *cis* isomers, increased thermal half‐lives of *cis*‐isomer (2.4 days), and increased stability against reduction in water [25, 26, 27, 28]. Hecht and co‐workers also exploited the *ortho*‐effect and synthesized tetra‐*ortho*‐fluoro azobenzenes, which showed *trans*‐to‐*cis* isomerization upon direct absorption of 530 nm light [29] and via two‐photon absorption at 640 nm, albeit using intense laser excitation [30]. Szymanski, Lützel, and coworkers reported synthetic protocols to design and synthesize *ortho*‐chloro or chloro/fluoro substituted azobenzenes and effect of substituent position on the *trans‐to‐cis* photoswitching behavior upon excitation with red light [16, 17]. Recently, Klajn, Priimagi, and co‐workers reported so‐called disequilibration by sensitization under confinement (DESC), where *trans*‐to‐*cis* isomerization of azobenzenes was made possible with green‐red or yellow light via triplet‐sensitization [31, 32]. While the *trans*‐to‐*cis* isomerization of the above‐mentioned DESC azobenzene derivatives was achieved with green, yellow, or red light, the action spectrum of the *cis*‐to‐*trans* isomerization remained in the blue region [25, 31, 32].

Efforts toward shifting the action spectrum of *cis*‐to‐*trans* isomerization in the red or NIR region have largely focused on molecular engineering of azobenzene and conversion via non‐linear optical processes, such as 2‐ or 3‐photon absorption (2PA or 3PA) or triplet sensitization [16, 17, 18, 19, 22, 23, 24]. In this regard, Gorostiza and co‐workers reported *cis*‐to‐*trans* isomerization of an azobenzene drug with 2PA [22, 33] or 3PA [34] upon excitation with NIR‐I or NIR‐II light, respectively [22, 33, 34]. Priimagi and coworkers reported molecular iodine‐induced photo‐electrocatalytic *cis*‐to‐*trans* isomerization of dimethoxyazobenzene upon 770 nm excitation [35]. Recently, Durandin [23] and Moth‐Poulsen, Bharmoria [24] and coworkers reported endothermic triplet‐sensitized *cis*‐to‐*trans* isomerization of azobenzenes upon excitation with 740–770 nm light [23, 24]. However, the action spectrum of *trans*‐to‐*cis* isomerization in these systems remains in the UV‐region [22, 23, 24, 25, 26, 27, 28, 29, 30, 31, 32, 33, 34, 35].

Therefore, simultaneous bidirectional switching of azobenzenes outside the UV‐blue region in a single aqueous system remains a challenge. In this direction, Ellis‐Davies and co‐workers used 2PA as a tool for bidirectional switching (*cis*↔*trans*) of a tetra‐fluoroazobenzene‐based ion channel antagonist with NIR light (780 and 900 nm) in HEPES buffer [36]. However, while 2PA is a useful tool, the use of high‐excitation‐power lasers in biological systems is rather unattractive due to thermal and optical degradation effects. Durandin and coworkers achieved bidirectional (*cis*↔*trans*) switching of a bridged azobenzene derivative, diazocine, via triplet‐sensitized photoswitching with green (530 nm) and far‐red (740 nm) light in deaerated organic solvents [37]. However, practical applications in photopharmacology demand such systems to function effectively in an aqueous environment where the generation of singlet‐oxygen presents an additional challenge.

**FIGURE 1:**
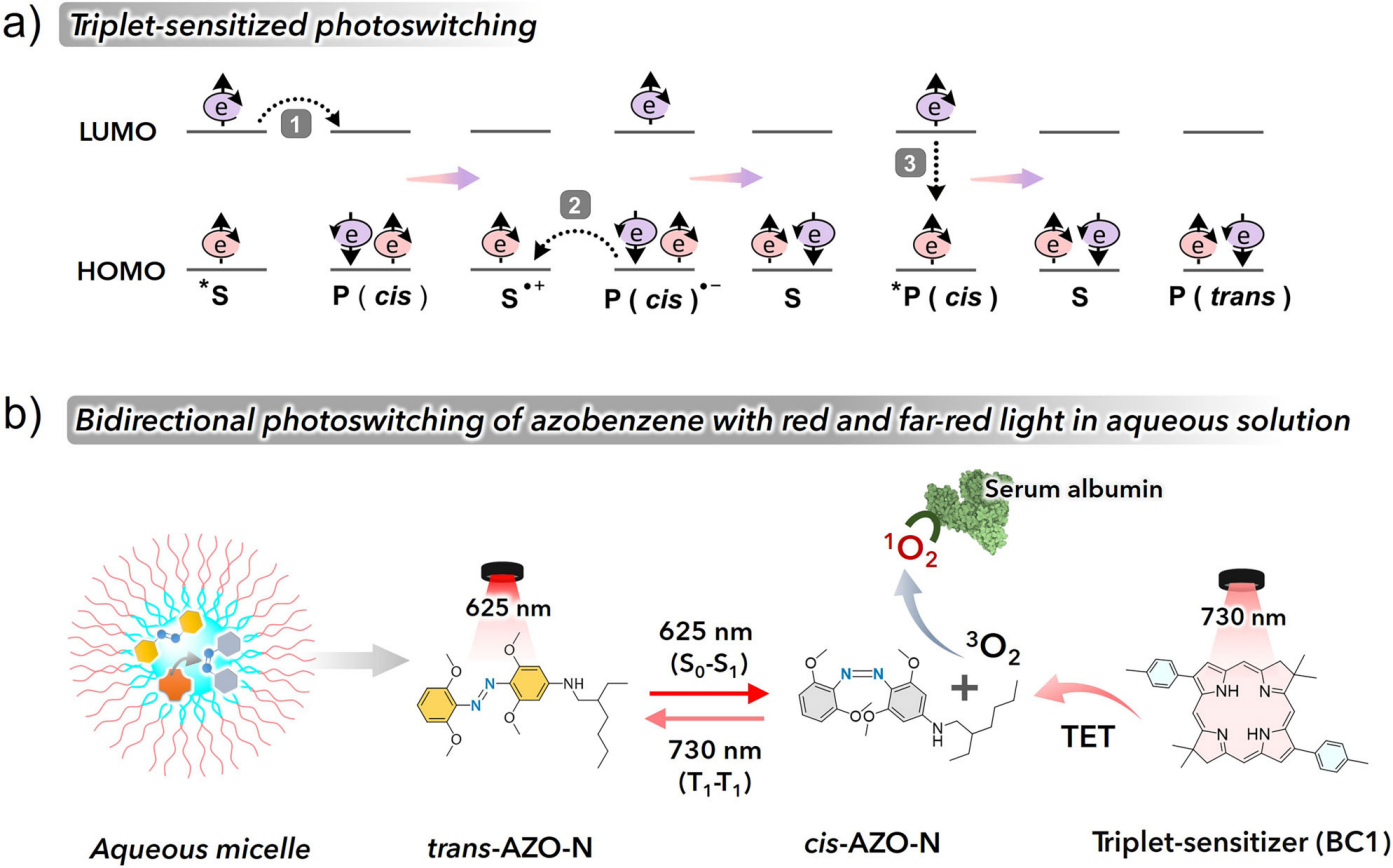
(a) Illustration of the proposed mechanism of the electron (e) exchange between photosensitizer (S) and photoswitch (P) during triplet‐sensitized *cis*‐to‐*trans* photoswitching. (b) Illustration of the modular design for bidirectional photoswitching of azo‐derivative in aqueous micellar solution upon excitation with red and far‐red light via singlet absorption (S_0_‐S_1_), and triplet sensitization (T_1_‐T_1_). During the triplet sensitization of *cis*‐**AZO‐N**, photosensitizer also transfers triplet energy to the ground state of triplet oxygen (^3^O_2_), which produces harmful singlet oxygen (^1^O_2_) to create oxidative stress, which however can be addressed by serum albumin present in the plasma.

Triplet‐sensitized photoswitching (TSP) entails a one‐step Dexter energy‐transfer process, wherein a photosensitizer in the excited triplet state transfers energy to the photoswitch via electron exchange, thereby inducing geometrical isomerization of the photo‐switch via triplet manifolds (Figure 1a) [38, 39, 40, 41, 42, 43]. Due to the relatively low excitation intensities and tunable excitation wavelengths, TSP is an emerging tool in photopharmacology for spatiotemporal control of biological photoactivation [38, 39]. For instance, Thorn‐Seshold and co‐workers demonstrated in vitro photocontrol of SNAP‐mGluR2 expression in granule cells of the dentate gyrus brain slices due to triplet‐sensitized *cis*‐to‐*trans* photoisomerization of a bioactive azobenzene‐photosensitizer dyad upon irradiation with 660 or 740 nm light [38]. Moth‐Poulsen, Bharmoria, and coworkers reported utilization of a triplet‐sensitization strategy to regulate the heart rate of a frog tadpole through the *cis*‐to‐*trans* photoisomerization of an azobenzene‐modified muscarinic acetylcholine receptor M2 agonist upon 730 nm excitation at low photon fluence (42 mW cm^−2^) [39]. However, a few key challenges remain to achieve enhanced utility: 1) an action spectrum of azo‐derivative for *trans*‐to‐*cis* isomerization outside the UV region, 2) a short half‐life of the *cis*‐isomer, 3) spectral overlap of photoswitches with the photosensitizers, and 4) limiting generation of singlet oxygen (^1^O_2_) during the triplet‐sensitization to minimize oxidative damage during in vivo application [44].

This study involves a comprehensive experimental investigation aimed at addressing these challenges. Herein, a bidirectional (***trans*** ↔ ***cis***) photoswitching with red and far‐red light has been achieved with a new lipophilic tetra‐*ortho*‐methoxy azobenzene derivative (**AZO‐N**) in organic and bio‐relevant aqueous solution via singlet absorption and triplet‐sensitization at excitation intensities lower than the tolerance limits of skin (200 mW cm^−2^) [45], illustrated in Figure 1b. Hence, the action spectrum of azobenzene is shifted toward the bio‐optical window.

Overall, this work investigates various challenges associated with the triplet‐sensitized photopharmacology in biological settings, including molecular design of the azobenzene photoswitch and photosensitizers, chromophores aggregation, spectral overlap of photosensitizers and photoswitches, photobleaching, and singlet oxygen generation and presents modular design principles to develop practically functional azo‐based photopharmaceuticals.

## 2 Results and Discussion

While the red‐light‐based *trans*‐to‐*cis* active azobenzenes are known [16, 17, 25, 26, 27, 28, 29, 30, 31], overlap of the triplet energies of *cis* and *trans* isomers is the likely impediment for their triplet‐sensitized back‐conversion (*cis*‐to‐*trans*) in the same solution. This is due to the interference of photosensitizer's absorption with the forward‐switching with redlight [24]. Therefore, this study took off with the synthesis of a new *tetra*‐*o*‐methoxy azobenzene‐derivative having separate triplet energies of *cis* and *trans* isomers. In addition, we identified triplet photosensitizers having Q‐band absorption >660 nm in the far‐red region and triplet‐energies closer to *cis*‐isomer to avoid interference with the *trans*‐to‐*cis* isomerization with 625 nm by redlight. The feasibility of bidirectional switching of azobenzene with red/far‐red light was tested in the deaerated organic solvents to prove the concept. Finally, it is worth emphasizing that the proposed designs were tested in a biorelevant aqueous medium for potential photopharmacological applications, including the effect of singlet‐oxygen generation during triplet‐sensitization experiments.

### 2.1 Azobenzene and Photosensitizers

**FIGURE 2:**
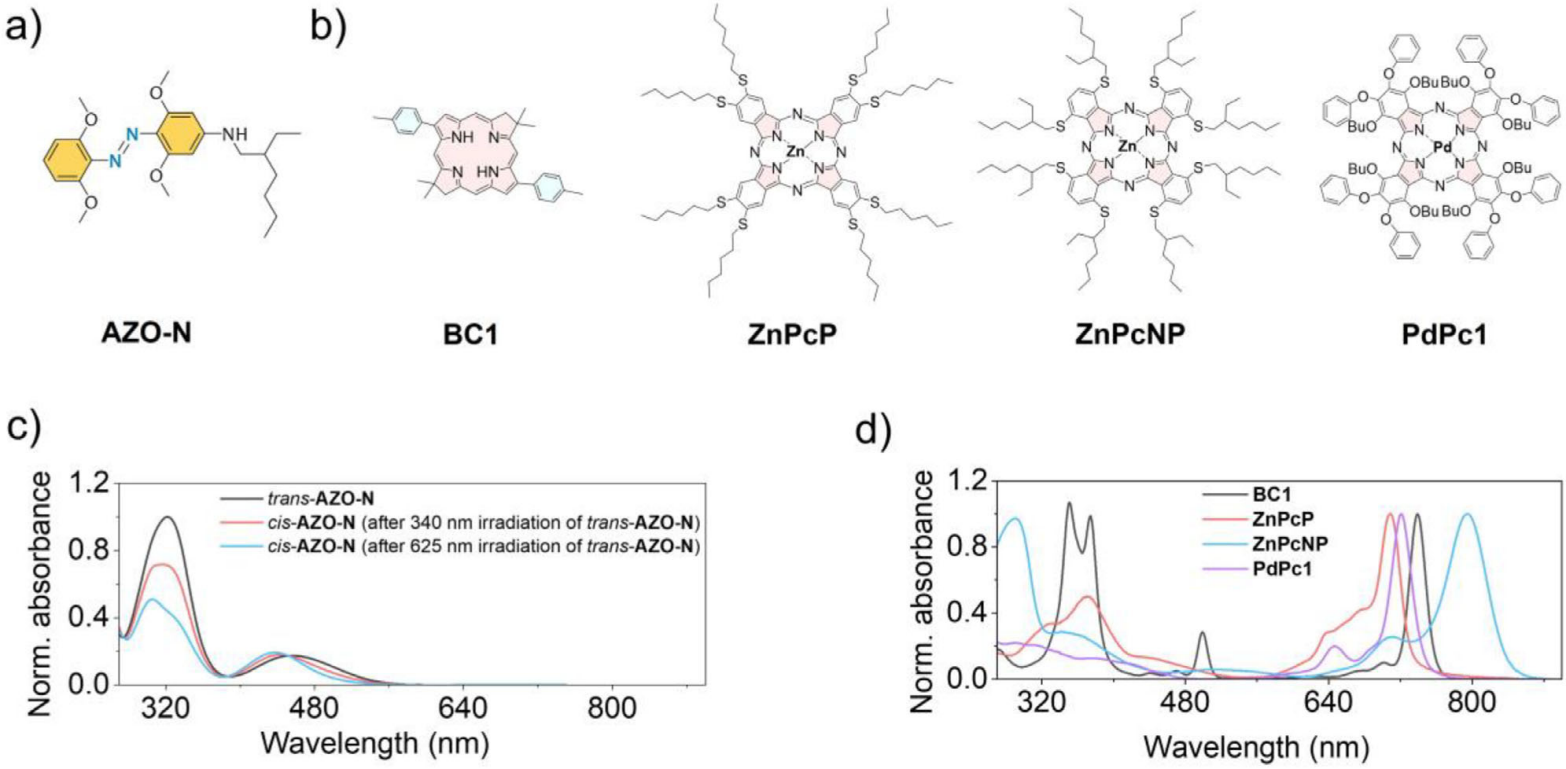
(a,b) Molecular structures of chromophores. a) *trans*‐**AZO‐N**, and (b) photosensitizers, **BC1**, **ZnPcP**, **ZnPcNP**, and **PdPc1**. (c,d) Normalized absorption spectra of chromophores. (c) *trans* and *cis*‐**AZO‐N** (λ_Norm._ = 318 nm), and (d) photosensitizers, **BC1** (λ_Norm._ = 739 nm), **ZnPcP** (λ_Norm._ = 708.5 nm), and **ZnPcNP** (λ_Norm._ = 795 nm) in DMSO, and **PdPc1** (λ_Norm._ = 721 nm) in cyclohexane.

A new lipophilic tetra‐*ortho*‐methoxy azobenzene derivative (**AZO‐N**) has been synthesized in a two‐step reaction with a yield of 25% (Scheme S1; Figure 2a). The synthesis and characterization of **AZO‐N**, which include ^1^H NMR, ^13^C{^1^H} NMR, HR‐MS and DSC, are available in the Supporting Information (Figures S1–S7). The DSC thermogram of **AZO‐N** shows the melting temperature at 91°C (Figure S7). The UV–vis spectrum of *trans*‐**AZO‐N** (dark) in dimethylsulfoxide (DMSO) exhibits π‐ π* absorption at 318 nm (ε = 14 000 M^−1^ cm^−1^), and n‐π* absorption maximum at 448 nm (ε = 2900 M^−1^ cm^−1^), with tail ending beyond 610 nm (ε = 0.84 M^−1^ cm^−1^) as shown in Figures S9a,b and S10. A low ε in the red‐region is typical for all *ortho*‐substituted azobenzenes [16, 17]. Excitation with 340 or 625 nm light induces *trans*‐to‐*cis* isomerization, which is indicated by the separation of the n‐π* absorption band with a blue shift of 12 or 18 nm, respectively. Additionally, there is a decrease in the π‐π* absorption with the change in band‐shape (Figure 2c).

The *ortho*‐substitution with a bulky electron donor like *o*‐methoxy increases the repulsive interactions with the lone‐pair on nitrogen atoms in the *trans*‐form, which destabilizes the n‐molecular orbital, followed by a red‐shift of n‐π* absorption due to the decrease in energy gap between n and π* orbitals [15, 27]. This effect also accounts for the slightly smaller vertical singlet–triplet energy gap of the *trans* isomer (T_1_ = 2.07 eV) compared to the *cis* isomer (T_1_ = 2.10 eV). However, the trend is reversed for the adiabatic singlet–triplet energy gaps, which are smaller for the *cis* isomer (T_1_ = 0.99 eV) than for the *trans* isomer (T_1_ = 1.24 eV) (Figure S8). This difference arises from the larger relaxation energy of the *cis* isomer (1.11 eV) relative to the *trans* isomer (0.83 eV). Upon excitation, the N═N bond elongates by 0.053 Å in cis‐**AZO‐N** and by 0.044 Å in trans‐**AZO‐N**. The greater structural rigidity of the trans form results in a smaller relaxation energy and consequently a higher adiabatic triplet energy. In contrast to Woolley's molecule [26], the **AZO‐N** exhibited a relatively small separation between the n‐π* absorption bands of its *cis* and *trans* isomers, in addition to a red‐shifted action spectrum tail (560 to ≥ 600 nm) of *trans*‐to‐*cis* isomerization.

Four different photosensitizers (Figure 2b), one bacteriochlorin [46] and three phthalocyanines [24, 39, 47] having Q‐band absorption in the far‐red or near infrared region, were investigated to shift the action spectrum of *cis*‐to‐*trans* isomerization to the far‐red region (730 nm) via triplet sensitization in DMSO/cyclohexane and aqueous phosphate buffer solutions (Figure 2d; Figures S11 and S12). The absorption spectrum of **BC1** in DMSO shows a Q_y_ band (S_0_ – S_1_) in the far‐red region (739 nm), the Q_x_ band (S_0_‐S_2_) in the blue‐green region (499 nm), and nominal B_x_ (S_0_‐S_3_), and B_y_ (S_0_ – S_4_) bands in the blue‐violet (Soret) region at 374 and 355.5 nm, respectively (Figure 2d) [46]. **ZnPcP** shows a Q‐band maximum at 708.5 nm and a UV‐A absorption band at 370.5 nm [24]. **ZnPcNP** shows a Q‐band maximum in the NIR region at 795 nm, and UV‐B absorption band at 290.5 nm [39]. In contemplation of possible biological applications, absorption spectra of photosensitizers in an aqueous buffer solution were also recorded (Figures S11 and S12). Compared to DMSO, the Q‐band absorption of **BC1** in buffer solution shows a blue shift of only 4 nm, while a considerable blue shift of 51 nm of the Q‐band was observed for **ZnPcP** due to H‐aggregation [24, 48, 49], making it ineffective for *cis*‐to‐*trans* photoswitching via triplet sensitization with 730 nm light. Therefore, **ZnPcNP** along with **BC1** was used for aqueous triplet‐sensitization studies. **ZnPcNP** showed a relatively small blue shift (18 nm) of the Q‐band, thus making it suitable for triplet sensitization with 730 nm light (Figure S11). Pd‐octabutoxy‐octaphenoxy phthalocyanine (**PdPc1**) [47] having Q‐band absorption at 721 nm was used as a fourth photosensitizer. Due to its higher photostability and non‐aggregating nature in the aqueous micellar solution (Figure S12), **PdPc1** was used to avail correct information about triplet‐energy transfer using time‐resolved emission decay experiments.

### 2.2 Triplet Energies of Photosensitizers and **AZO‐N**

The triplet energies of the photosensitizers were estimated from the wavelength of the maximum of the time‐resolved phosphorescence spectra. The triplet energy of **BC1**, **ZnPcP**, **ZnPcNP**, and **PdPc1** was thus estimated to be 1.1 eV (1110 nm), 1.1 eV, 1.0 eV (1220 nm), and 1.1 eV, respectively. The phosphorescence lifetime calculated from single‐exponential fitting of the time‐resolved decay profiles of **BC1**, **ZnPcP**, **ZnPcNP**, and **PdPc1** were respectively 94, 178, 38, and 3.5 µs in argon‐saturated non‐polar solvents (Figure S13a–d). For comparison, we also calculated adiabatic relative energies of the triplet state of **BC1** and **ZnPcP** using the M06‐2X [50] functional with the 6–311G(d,p) basis set [51] and found values of 1.05 and 1.16 eV, respectively (Figure S8), which are in close proximity to the experimental triplet energies (Figure S13). This prompted the use of the M06‐2X/6–311G(d,p) method to also calculate the triplet energies of **AZO‐N**, which are not possible to measure experimentally. The triplet energies of *cis* and *trans* **AZO‐N** were calculated to be 0.99 and 1.24 eV, respectively (Figure S8). Hence, *cis*‐**AZO‐N** has a suitable triplet energy for triplet sensitization via an exothermic route with the photosensitizers under study.

### 2.3 Photoswitching Studies

Photoswitching studies were carried out in deaerated DMSO/toluene, and aerated 2% Pluronic F‐127 (**PF‐127**) micellar solution in phosphate buffer (10 mm) at pH 7.4. **PF‐127** micelles were used because of their significant oxygen impermeability, thereby protecting the triplet state of chromophores from quenching [39, 52]. The emission spectra of all LEDs used in this work (365, 455, 625, and 730 nm) are provided in Figure S14. Various photophysical parameters of **AZO‐N** in the dark, photostationary state, and during *trans‐cis*‐*trans* isomerization in DMSO are summarized in Table 1.

**TABLE 1:** Summary of various photophysical parameters of **AZO‐N** in the dark, photostationary state, and during *trans‐cis*‐*trans* isomerization in DMSO.

AZO‐N	λ_*max*_	ε (*M* ^−1^cm^−1^)	*trans*	*cis*	T_1_ (eV)	*t*_1/2_
*trans*(π − π*)^#^	318 nm	14 000	52%	48%	1.24	—
*trans*(*n* − π*)^#^	448 nm	2900	52%	48%	1.24	—
*trans*(*n* − π*)^#^	448 nm	0.84 (610 nm)	52%	48%	1.24	—
*trans*(π − π*)^&^	322 nm	—	79%	21%	—	—
*trans*(*n* − π*)^&^	456 nm	—	79%	21%	—	—
*cis*(π − π*)^$^	316 nm	—	20%	64%	—	—
*cis*(*n* − π*)^$^	443 nm	—	20%	64%	—	—
*cis*(π − π*)^£^	305 nm	—	12%	88%	0.99	—
*cis*(*n* − π*)^£^	435 nm	—	12%	88%	0.99	—
*trans* to *cis* ^$^	—	—	—	—	—	24 s
*trans* to *cis* ^£^	—	—	—	—	—	18 min
*cis* to *trans* ^&^	—	—	—	—	—	7 s
*cis* to *trans* ^¥^	—	—	—	—	—	8–14 min
*cis* to *trans* (Δ)	—	—	—	—	—	27 days

# – dark‐state; & – after 455 nm excitation; $ – after 340 nm excitation; £ – after 625 nm excitation; ¥ – after 730 nm excitation; Δ – at room temperature

#### 2.3.1 Sensitizer‐Free Photoswitching of **AZO‐N** in the UV–vis Region

First, we investigated the ***trans*** ↔ ***cis*** photoswitching of **AZO‐N** in DMSO at its absorption wavelengths in the UV–vis region (340 or 455 nm) as shown in Figure S15a,b. In the dark, **AZO‐N** exists chiefly as the thermodynamically stable state, having % *trans* = 52%, and % *cis* = 48% (Figure S16, black line, and Figures S17 and S18 (^1^H NMR) and Table S1). Upon irradiation at 455 nm, the dark state changes to the PSS with an increase in % *trans*‐**AZO‐N** up to 79% (Figure S16 red line and Figures S17 and S18, and Table S1). This state of **AZO‐N** has been used to study the ***trans*** ↔ ***cis*** photoswitching. The absorption profiles of **AZO‐N** show reversible ***trans*** ↔ ***cis*** photoswitching upon excitation with 340 or 455 nm light (Figure S15a,b). The thermal half‐life of *cis*‐**AZO‐N** in DMSO at room‐temperature was found to be 23 days (Figure S19). Together, these features confirm the relevance and suitability of the use of such AZO derivatives in photopharmacological studies [8].

**FIGURE 3:**
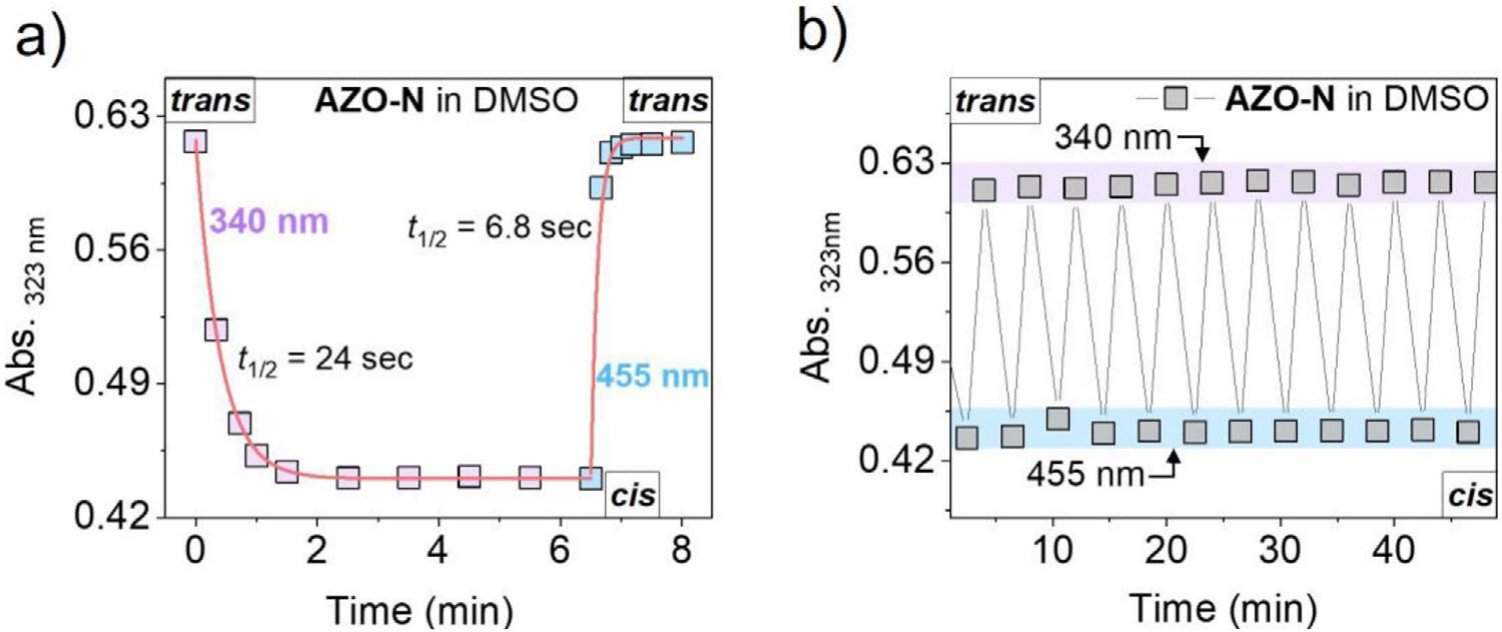
(a) Plot showing kinetics of ***trans*** ↔ ***cis*** photoswitching of **AZO‐N** dissolved in DMSO upon excitation with 340 nm (0.76 mW cm^−2^) or 455 nm (3.05 mW cm^−2^) light. (b) Cyclic ***trans*** ↔ ***cis*** photoswitching of **AZO‐N** upon periodic excitation with 340 nm (2.5 min) or 455 nm (1.5 min) light. **AZO‐N** = 36 µm.

The half‐lives (*t*_1/2_) of *trans‐to‐cis* and *cis‐to‐trans* photoswitching were determined to be 24 and 6.8 s, respectively, through the single‐exponential fitting of the photo‐kinetics profile (Figure 3a). It is important to note that the half‐life of photoswitching depends on molecular concentration and the light intensity, and can therefore be controlled according to specific requirements [24]. This is because at higher concentration, chromophores can undergo π–π stacking, leading to chromophore aggregation (J or H), which affects their extinction coefficient, and hence light absorptivity [53, 54]. The photostability of **AZO‐N** in DMSO was confirmed by measuring photoswitching across 12 successive cycles of periodic excitation with 340 or 455 nm light for 48 min without any measurable degradation (Figure 3b; Figure S20).

Next, *trans‐to‐cis* photoswitching of **AZO‐N** was investigated upon excitation with red light in aerated DMSO. **AZO‐N** shows *trans*‐to‐*cis* photoswitching upon 625 nm excitation (*I*_ex_ = 154 mW cm^−2^), an action spectrum of *trans*‐to‐*cis* isomerization outside the UV‐region. The half‐life of *trans*‐to‐*cis* photoswitching was found to be 18 min (Figure S21). The slow kinetics is attributed to the low absorption coefficient of **AZO‐N** in the red region. However, this is an attractive feature, as slow photoisomerization kinetics is highly desired for some photopharmacological studies [8], to avoid possible alteration of the biological environment. The average photoisomerization quantum yield of *trans*‐to‐*cis* isomerization upon 625 nm LED irradiation was calculated using our custom‐built automation setup [55] and was found to be QY = 1.03±0.08% for the analysis wavelength of 530 nm (Figures S22–S25). The ^1^H NMR spectra revealed 88% of *cis*‐**AZO‐N** in the PSS upon 40–50 min of irradiation with 625 nm LED (Figures S26 and S27 and Table S2). Further analysis in toluene‐*d*_8_ (Figures S28–S33 and Tables S3 and S4) revealed that a maximum 65% of *cis*‐**AZO‐N** could be reached upon 340 nm irradiation of *trans*‐**AZO‐N**, which is lower than upon irradiation at 625 nm. These results corroborate the amount of absorbance change due to photoswitching upon 340 or 625 nm irradiation of *trans*‐**AZO‐N** (Figure 2c; Figure S16).

#### 2.3.2 BiDirectional Photoswitching of **AZO‐N** in the Red/Far‐Red Region

**FIGURE 4:**
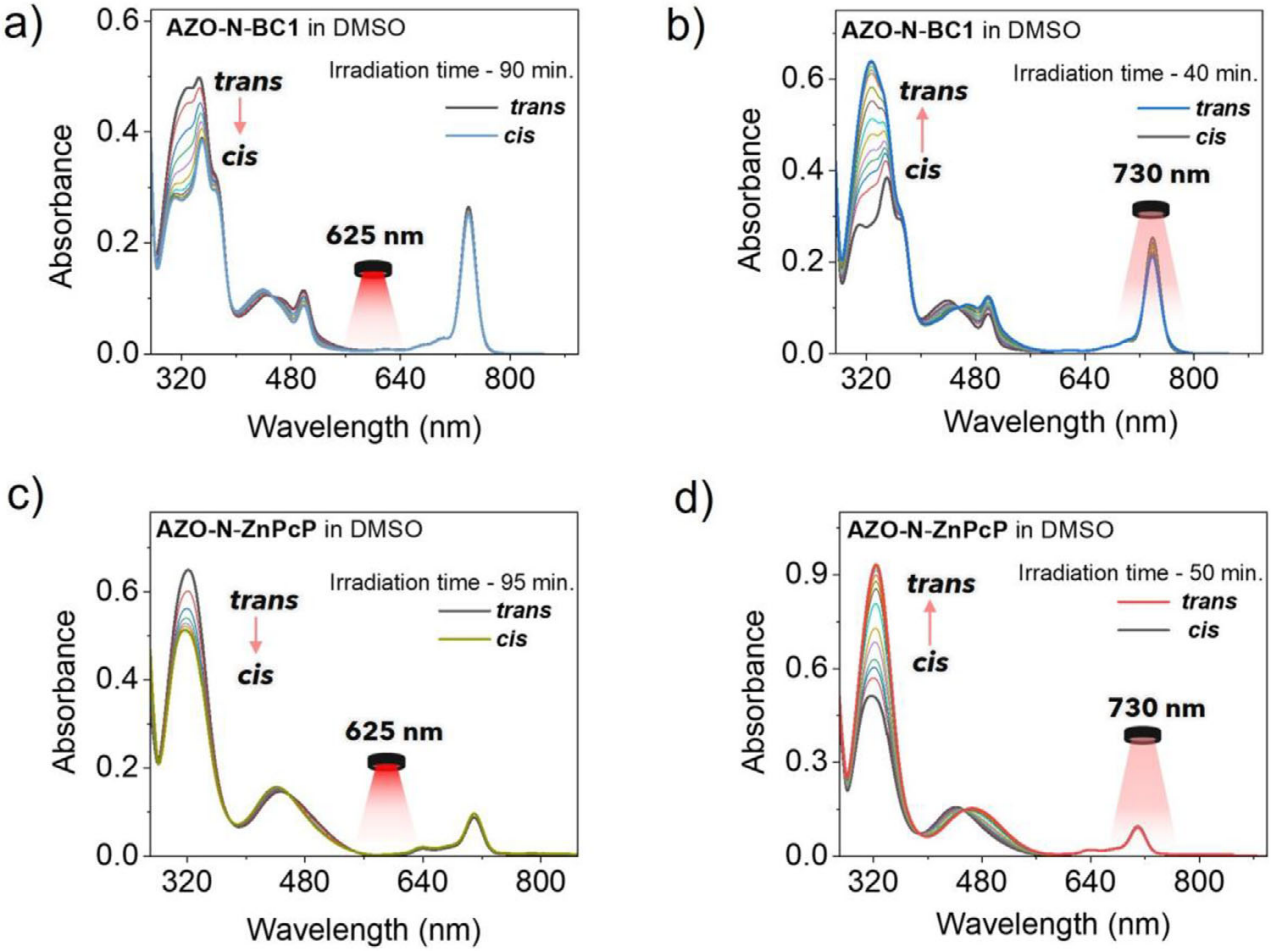
Absorption profiles of (a,b) degassed **AZO‐N**‐**BC1**‐DMSO solution and (c,d) degassed **AZO‐N**‐**ZnPcP**‐DMSO solution showing ***trans*** ↔ ***cis*** photoswitching of **AZO‐N** upon 625 nm (154 mW cm^−2^) and 730 nm (116 mW cm^−2^) LED excitation.

To achieve bidirectional photoswitching within the phototherapeutic window, a triplet‐sensitization method was utilized to shift the action spectrum of *cis*‐to‐*trans* photoswitching from the blue to the far‐red region. For this, photosensitizers (**BC1**, **ZnPcP**, or **ZnPcNP**) were mixed with **AZO‐N** in DMSO/toluene or **PF‐127** (2%) PBS solution. Figure 4a–d shows absorption profiles indicating ***trans*** ↔ ***cis*** photoswitching of **AZO‐N** in degassed **AZO‐N**‐**BC1**‐DMSO and **AZO‐N**‐**ZnPcP**‐DMSO solutions upon excitation with either 625 or 730 nm light. **AZO‐N** showed bidirectional ***trans*** ↔ ***cis*** photoswitching in both solutions upon 625 or 730 nm excitation (Figure 4a–d). While *trans*‐to‐*cis* photoswitching occurs via singlet absorption of 625 nm light by *trans*‐**AZO‐N**, *cis*‐to‐*trans* photoswitching occurs through triplet‐sensitization of the *cis*‐**AZO‐N** by **BC1** or **ZnPcP** upon 730 nm excitation. The photo‐kinetic profiles (Figure S34) upon illumination at 625 or 730 nm revealed respective half‐lives of 15 min or 8 min for ***trans*** ↔ ***cis*** photoswitching in **AZO‐N**‐**BC1**‐DMSO and 26.5 or 13.4 min in **AZO‐N**‐**ZnPcP**‐DMSO solution.

**FIGURE 5:**
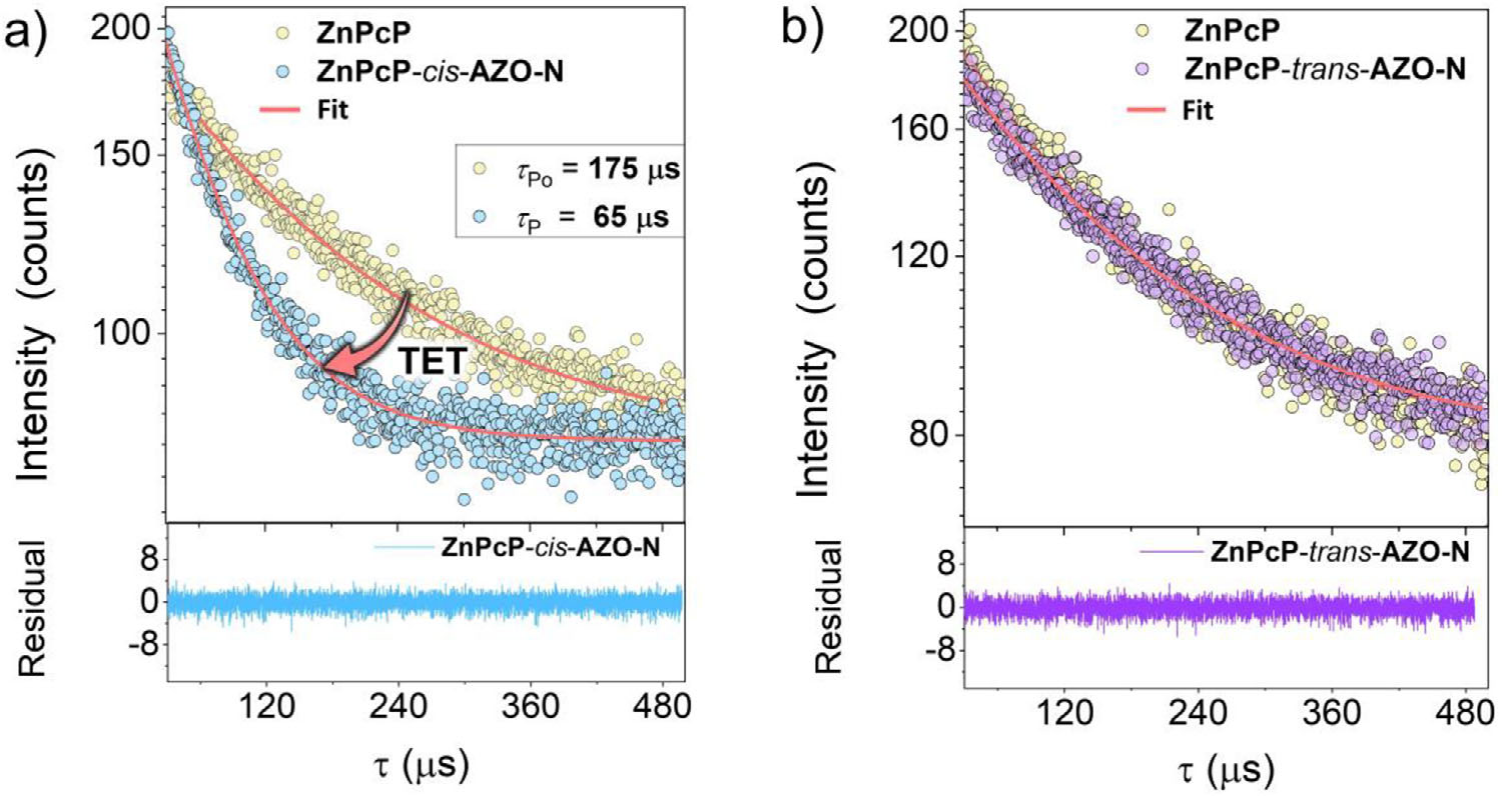
Time‐resolved phosphorescence decay profiles of **ZnPcP** in (a) **ZnPcP**‐*cis*‐**AZO‐N** and (b) **ZnPcP**‐*trans*‐**AZO‐N** solutions in argon saturated toluene. λ_ex_ = 664 nm pulsed laser. λ_em_ = 1110 nm. The decays could be adequately fitted by single‐exponential functions (red lines), as judged by the random distribution of residuals. **AZO‐N** = 36 µm; **ZnPcP** = 5 µm.

The involvement of triplet sensitization in *cis*‐to‐*trans* photoswitching was confirmed from the quenching of **ZnPcP** phosphorescence lifetime by *cis*‐**AZO‐N** (Figure 5a). Interestingly, unlike the non‐derivatized azobenzene, *trans*‐**AZO‐N** does not quench the triplet state of **ZnPcP**, as revealed by the overlapped decay profiles of **ZnPcP** and **ZnPcP**‐*trans*‐**AZO** solution (Figure 5b). This observation is consistent with the triplet energy of ZnPcP being higher than that of *cis*‐**AZO‐N**, but lower than that of *trans*‐**AZO‐N**, the triplet‐energy transfer therefore being exothermic, (Figure 1). The efficiency of triplet energy transfer (TET) was measured from the quantum yield of TET (ϕ_*TET*_). A ϕ_*TET*_ = 63 ± 1 % was calculated from the difference in phosphorescence lifetimes of **ZnPcP** in the absence (τ_*Po*_ = 175 ± 12 µs) and presence of *cis*‐**AZO‐N** (τ_*P*_ = 65 ± 7 µs) using Equation (1) [24, 56].

(1) $$\phi_{TET}=1-\frac{\tau_{P}}{\tau_{Po}}$$

The rate constant for quenching has been calculated using Equation (2) [52].

(2) $$k=\frac{1-\frac{\tau_{P}}{\tau_{Po}}\;}{\left[cis-AZO-N\right]}\;$$

The concentration of *cis*‐**AZO‐N** used in the quenching experiment is 36 µm. The resulting value of *k* = 1.7 ± 0.2  ×  10^10^ M^−1^ s^−1^ is consistent with a diffusional triplet‐triplet energy‐transfer rate constant [57]. This indicates that energy transfer from **ZnPcP** to *cis*‐**AZO‐N** is exothermic, in agreement with the calculated triplet energy of *cis*‐**AZO‐N** being lower than that of **ZnPcP**.

**FIGURE 6:**
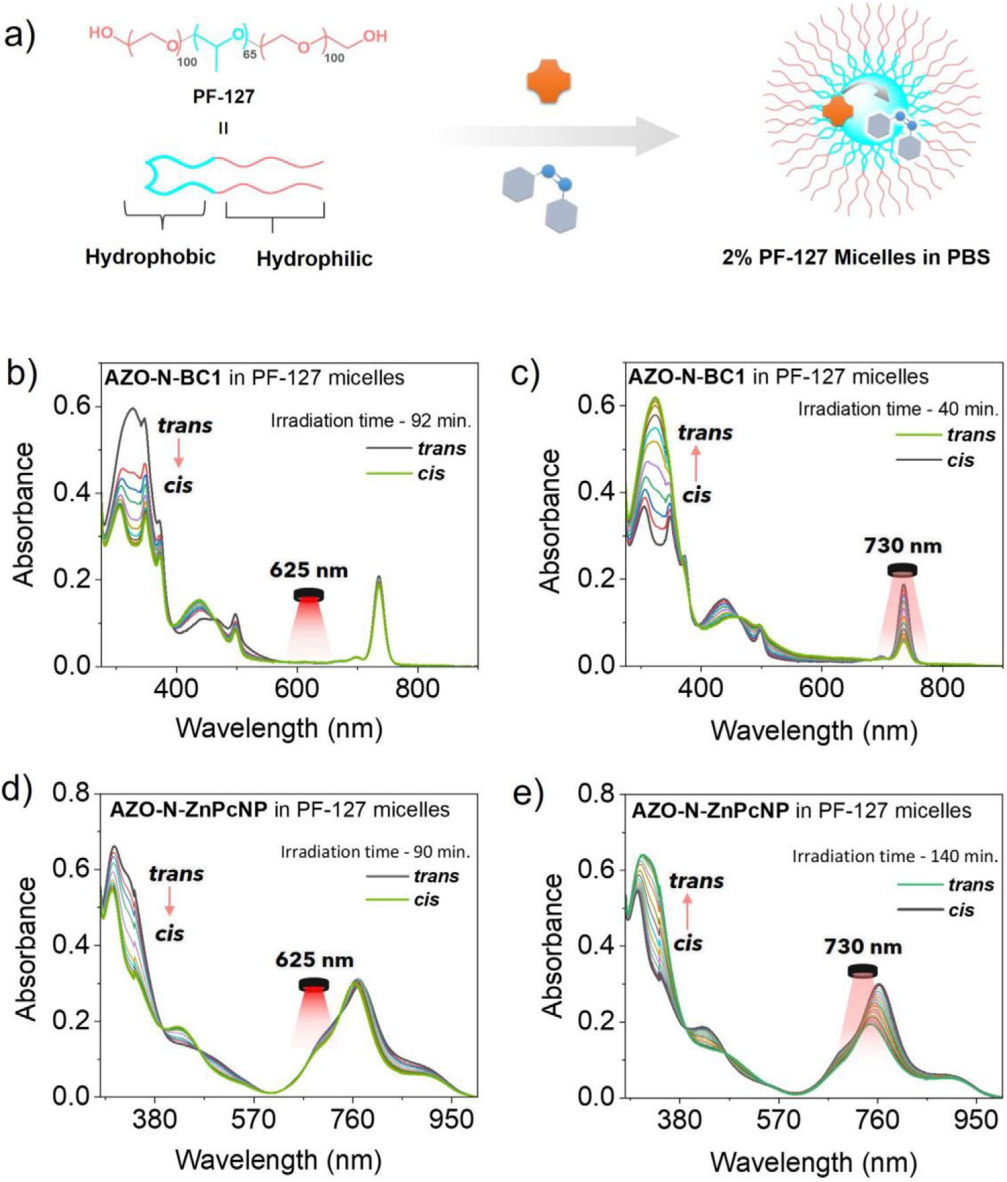
(a) Molecular structure of **PF‐127** and illustration of its micelles hosting photosensitizer and **AZO‐N** in the hydrophobic core region. Absorption profiles in aerated aqueous solution of: (b,c) **AZO‐N**‐**BC1** in **PF‐127** micelles and (d,e) **AZO‐N**‐**ZnPcNP** in **PF‐127** micelles showing ***trans*** ↔ ***cis*** photoswitching of **AZO‐N** upon 625 nm (154 mW cm^−2^) and 730 nm (116 mW cm^−2^) LED excitation.

Following experiments in deaerated organic solvents, bidirectional switching was investigated in an aqueous aerated environment using **PF‐127** (2%) phosphate buffer solution (10 mm, pH 7.4) (Figure 6a). The 2% **PF‐127** in PBS form micelles of the size of 5 nm. The size increased to 21 nm with the incorporation of **AZO‐N** and **ZnPcNP** in the micelles core (Figure S35). **PF‐127** was used because it is also used both as a solubilizing agent and a potential delivery agent for the photopharmacological drugs in vivo. Pluronic micelles are well‐known delivery agents for sustained drug release in vivo [58, 59]. Figure 6b–e shows absorption profiles indicating ***trans*** ↔ ***cis*** photoswitching of **AZO‐N** in **AZO‐N**‐**BC1**‐**PF‐127** and **AZO‐N**‐**ZnPcNP**‐**PF‐127** buffer solutions upon excitation with 625 or 730 nm light. **ZnPcNP** was chosen instead of **ZnPcP** because of the aggregation of the latter in **PF‐127** solution, which shifted the absorbance maximum to the *trans*‐to‐*cis* isomerization region (620–630 nm). **AZO‐N** showed bi‐directional ***trans*** ↔ ***cis*** photoswitching in both solutions upon 625 or 730 nm excitation due to singlet absorption or triplet sensitization of **AZO‐N** by the photosensitizers (Figure 6b–e).

Hence, the action spectrum of both ***trans*** ↔ ***cis*** isomerizations were shifted outside the UV‐blue region at excitation power densities within the limits of skin tolerance [39] (154 mW cm^−2^ of 625 nm, and 116 mW cm^−2^ of 730 nm light) in aqueous solution. The photo‐kinetic profiles (Figure S36) upon illumination at 625 or 730 nm revealed half‐lives of 16.9 or 16.5 min for ***trans*** ↔ ***cis*** photoswitching in **AZO‐N**‐**BC1**‐**PF‐127** and 20 or 34 min in **AZO‐N**‐**ZnPcNP**‐**PF‐127** solution, respectively. Durandin et al.’s work reported the bidirectional switching, but that was entirely based on triplet‐sensitization with two different photosensitizers (green and far‐red absorbing) in deaerated organic solvents (DMSO or chloroform)with diazocine as a photoswitch having opposite action spectrum to that of azobenzene [37]. However, our work shows bidirectional photoswitching of azobenzene derivative with more tissue penetrable red/far‐red light in the biological relevant PBS‐micellar media in aerated environments.

Triplet‐sensitization was achieved even in the presence of air, which is attributed to the location of the chromophores in the core of **PF‐127** cross‐linked micelles. Pluronic micelles are well‐known oxygen protectors and their oxygen blocking ability increases with an increase in concentration [52, 60]. Moreover, heating further increases the oxygen blocking ability due to cross‐linking between micelles chains, evidenced by a long‐lifetime of the photosensitizer (23 µ*s*), equivalent to that in the degassed DMF [52]. Further to show the oxygen protection effect of **PF‐127** micelles, we measured the kinetics of singlet oxygen production and decay using **BC1** as photosensitizer in **PF‐127** micelles at 1275 nm (Figure S37, black line). The signal rises with a lifetime of 1.2 µs and decays with a lifetime of 3.6 µs, which correspond to the triplet photosensitizer decay and singlet oxygen decay, respectively. The triplet lifetime is 4–5 times longer than the lifetimes typically observed in non‐aqueous solvents, e.g., THF, consistent with a lower accessibility of oxygen to the excited photosensitizer in the micelles. In the presence of *cis*‐**AZO‐N**, the intensity of the singlet oxygen signal decreased by 95% (Figure S37a), which sets an upper limit value for the singlet oxygen quantum yield in the presence of *cis*‐**AZO‐N** of $\Phi_{\Delta }\leq 0.05$ (Figure S37a). However, kinetic analysis reveals that both signals rise and decay with the same lifetimes, 1.1 and 3.6 µs, respectively. This indicates that **AZO‐N** quenches the production of singlet oxygen by static quenching, which, of course, is consistent with **BC1**, and **AZO‐N** is located in close proximity. It was also confirmed by quenching of the phosphorescence intensity of **BC1** at 1110 nm in the presence of *cis*‐**AZO‐N** (Figure S37b).

**FIGURE 7:**
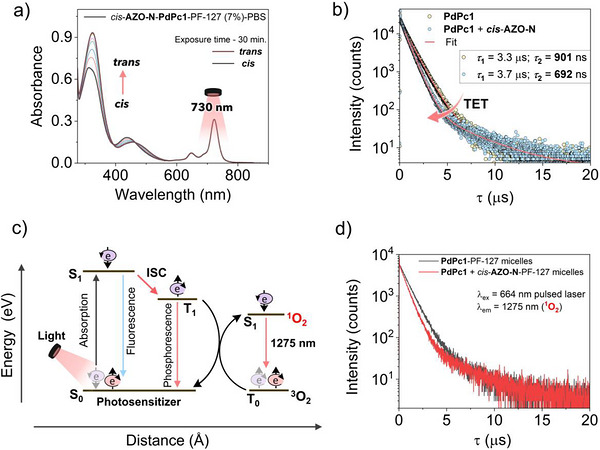
(a) Absorption profiles of **AZO‐N**‐**PdPc1‐PF‐127** micelles solution showing *cis* → *trans* photoswitching of **AZO‐N** upon 730 nm (116 mW cm^−2^) LED excitation. (b) Time‐resolved phosphorescence decay profiles of **PdPc1** in the absence and presence of *cis*‐**AZO‐N** in air‐saturated **PF‐127**‐PBS solution (λ_ex_ = 664 nm pulsed laser, λ_em_ = 1110 nm). The red line indicates double‐exponential fitting of decay profiles. (c) Illustration of the triplet‐sensitization reaction of molecular oxygen by the photosensitizer leading to the generation of singlet oxygen. (d) Time‐resolved emission‐decay profiles at 1275 nm in the absence and presence of **AZO‐N** in air‐saturated **PdPc1‐PF‐127**‐PBS solution. λ_ex_ = 664 nm pulsed laser. **PdPc1** = 1.2 µm. **AZO‐N** = 64.6 µm. The only noticeable change is the shortening of the fast decay component, i.e., quenching of the **PdPc1** phosphorescence by *cis*‐**AZO‐N**.

However, photobleaching or aggregation of sensitizers (**BC1** and **ZnPcNP**) during TET (Figure 6c,e) was a major issue for accurate measurements. This prompted the use of a more photostable photosensitizer, palladium phthalocyanine (**PdPc1**), which has the same triplet energy as **ZnPcP**. Unlike **ZnPcP**, **PdPc1** disperses monomerically in the **PF‐127** solution, confirmed from a similar absorption profile as that in cyclohexane (Figure S12) [47]. The **AZO‐N**‐**PdPc1**‐**PF‐127**‐**PBS** solution shows ***trans*** ↔ ***cis*** photoswitching in both solutions upon 625 or 730 nm excitation (Figure S38; Figure 7a). As can be seen from Figure 7a, no photobleaching of **PdPc1** absorbance was observed around 730 nm, indicating its photostability. The time‐resolved phosphorescence measurements (Figure 7b) showed quenching of the phosphorescence of **PdPc1**, whose triplet lifetime decreased from 901 to 692 ns in the presence of *cis*‐**AZO‐N**, indicating triplet‐energy transfer.

These results provided important information about the energy‐transfer mechanism in aqueous environments due to the co‐localization of **AZO‐N** and **PdPc1** in the core of the micelles.

Further photoisomerization quantum yields (*QY*_*trans*↔*cis*_) in air‐saturated aqueous solution were determined for the **AZO‐N**‐**PdPc1‐ PF‐127** dissolved in PBS solution (10 mm, pH 7.4) upon 625 nm/730 nm LED excitation (Figures S39 and S40). Measurements were performed by recording absorbance changes in our in‐house setup [55]. The *QY*_*trans* → *cis*_ was calculated to be 0.21% and *QY*_*cis* → *trans*_ via triplet‐sensitization was calculated to be 0.031% (Figures S39 and S40). While the emission wavelength of 625 nm LED is broad (625±20 nm), *trans*‐to‐*cis* photoswitching was also performed using a 633 nm red laser (*I*_ex_ = 54 mW cm^−2^). The **AZO‐N**‐**PdPc1‐ PF‐127**‐PBS solution showed *trans*‐to‐*cis* photoswitching, thus demonstrating the conversion by deep‐redlight (Figure S41).

## 3 Singlet‐Oxygen Generation During Triplet‐Sensitized Photoswitching

Another important factor while exploring TSP for photopharmacology is the amount of ^1^O_2_ generated during a secondary triplet‐sensitization reaction with ground‐state (triplet) oxygen (Figure 7c) [61]. The emission of ^1^O_2_, both in the presence or absence of **AZO‐N**, is undetectable compared to the phosphorescence of **PdPc1** (Figure 7d), indicating that the production of ^1^O_2_ in this system could be a residual process as **AZO‐N** quenches the phosphorescence of **PdPc1**.

**FIGURE 8:**
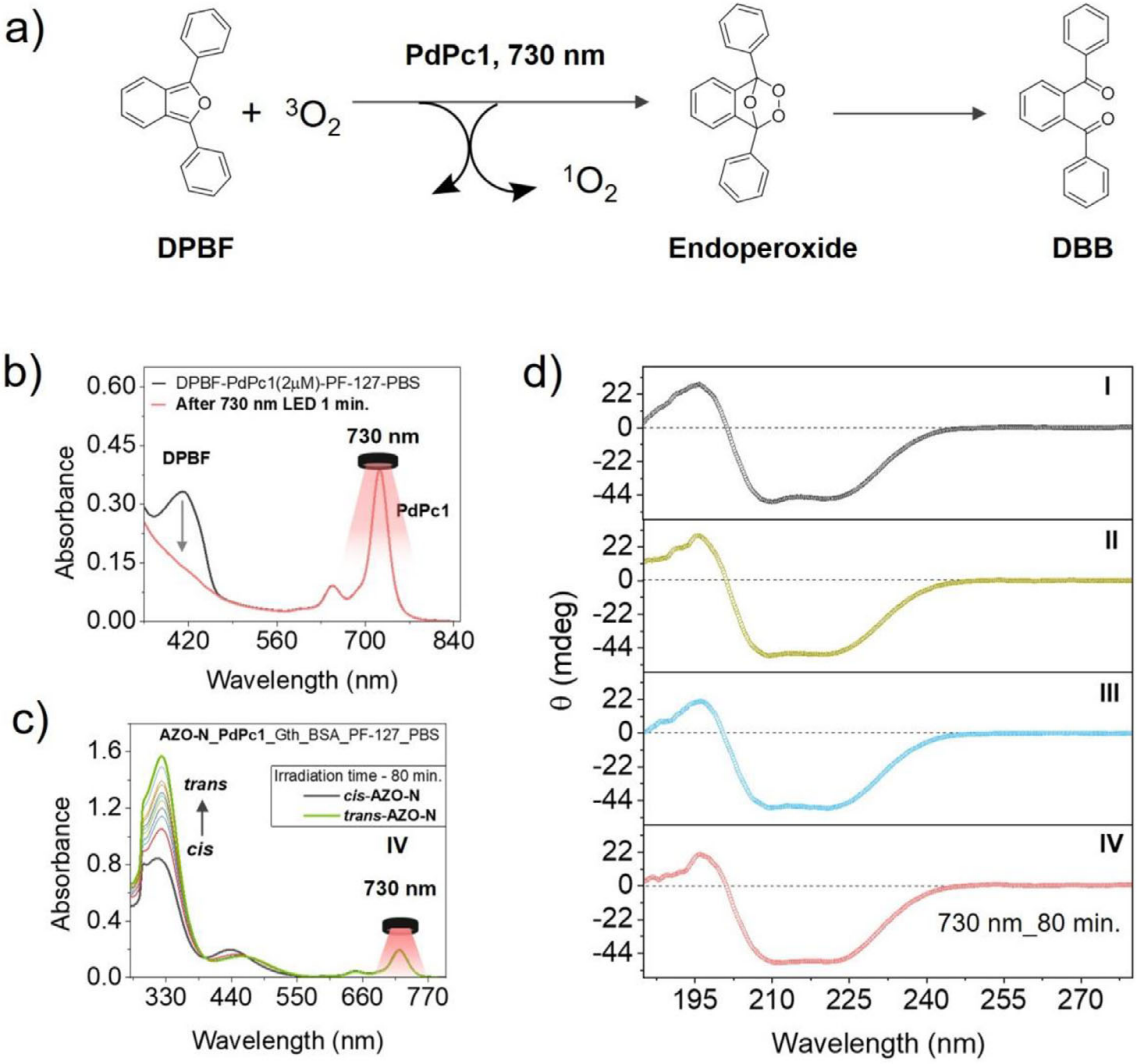
(a) Schematic of the reaction of **DPBF** with a ^1^O_2_ generated during photosensitization reaction. (b) Absorption spectra showing quenching of **DPBF** absorbance in the presence of **PdPc1** upon excitation with 730 nm in **PF‐127** micelles solution in PBS. (c) Absorption spectra showing triplet‐sensitized photoswitching of *cis*‐**AZO‐N** in the presence of ^1^O_2_ quenchers, glutathione (1 mm), and bovine serum albumin, BSA (500 µm) in **PF‐127**‐PBS solution (10 mm, pH 7.4). (d) Circular dichroism spectra of BSA (5 µm). I → in PBS; II→ **PF‐127** (2%)‐PBS; III and IV →**AZO‐N**‐**PdPc1**‐glutathione‐**PF‐127**‐PBS, before irradiation (III), and after irradiation with 730 nm light (IV). **DPBF** = 50 µm, **AZO‐N** = 76 µm, **PdPc1** = 1 µm, Glutathione = 1 mM, BSA = 500 or 5 µm.

However, to be sure, we also investigated ^1^O_2_ generation via chemical method using 1,3‐diphenylisobenzofuran (**DPBF**) as probe (Figure 8a,b; Figure S42) [62, 63]. First, we investigated the **DPBF** quenching in toluene (Figure S39). The **DPBF** showed a decrease in absorbance in the presence of **PdPc1** upon excitation with 730 nm light within a minute (Figure S42b). This is due to the formation of endoperoxide followed by decomposition to 1,2‐dibenzoylbenzene (**DBB**), shown in Figure 8a [62].

A minimal change in absorbance in the absence of **PdPc1** confirmed the role of singlet‐oxygen for decreasing the **DPBF** absorbance (Figure S42c). The **DPBF‐PdPc1‐PF‐127**‐PBS solution shows similar quenching of **DPBF** upon excitation with 730 nm light, confirming ^1^O_2_ generation (Figure 8b). While the generated ^1^O_2_ does not affect the triplet‐sensitized *cis‐to‐trans* photoswitching of **AZO‐N** in **PF‐127** solution (Figures 6 and 7), it can create oxidative stress during in vivo studies [63].

To address this issue, TSP experiments were performed in the presence of compounds present in the animal body to address the oxidative stress (glutathione and serum albumins) [64, 65, 66]. The **AZO‐N** showed efficient triplet‐sensitized *cis‐to‐trans* photoswitching in the solution comprising **AZO‐N**‐**PdPc1‐glutathione‐BSA‐PF‐127**‐PBS (Figure 8c).

Following this, the change in secondary structure of BSA due to possible interaction with ^1^O_2_ was investigated (Figure 8d; Figure S43). The concentrations of BSA and glutathione were kept within the blood plasma unit during triplet‐sensitization experiments. Interestingly, no significant change in the fingerprint all‐*α* CD spectra of BSA was observed after irradiation with 730 nm (IV), compared to non‐irradiated solutions (I‐III). The bands corresponding to *α*‐helical structure at ‐*θ*_210nm_ and ‐*θ*_220nm_ and cross‐*β* structure at +*θ*_196nm_ did not show any shifts [67, 68] (Figure 8d). The accuracy of CD spectra was confirmed from HT[V] below 750 (Figure S42). While this evidence shows a minimum oxidative effect of ^1^O_2_ generated during TSP, the living cell contains numerous other biomolecules, which may get affected. In our previous work, a minimal change in the heart rate of frog's tadpole was observed upon photoexcitation with 730 nm light in the presence of photosensitizer, **ZnPcNP**, for 30 min [39]. Therefore, more studies in animal models are required before drawing a concrete conclusion about the phototoxicity [8, 69] of the TSP process, which will be part of our future research.

## 4 Conclusions

The combination of the molecular engineering of an azobenzene chromophore and triplet‐sensitized photoswitching processes has afforded advancements in addressing several key challenges for the use of **AZO**‐based photopharmaceuticals. 1) A new, synthetic, lipophilic azobenzene derivative shows *trans*‐to‐*cis* isomerization upon excitation with red light (625±20 nm LED, or 633 nm laser), thereby effectively shifting the action spectrum outside the harmful UV region. 2) A successful *cis*‐to‐*trans* isomerization occurs in the same aqueous solution through triplet sensitization of *cis*‐**AZO‐N** from various photosensitizers present in a catalytic amount; excitation with far‐red light (730±20nm LED or 730 nm laser) also shifts the action spectrum of *cis*‐to‐*trans* isomerization outside the blue region. 3) The shift in the action spectrum of **AZO‐N** into the more tissue penetrable phototherapeutic window is achieved at low excitation densities (<200 mW cm^−2^) within biologically relevant aqueous systems. 4) The *cis*‐form of synthetic **AZO‐N** has a long half‐life of 23 days, which is highly desirable for photoactivable drugs during in vivo applications. Taken together, this proof‐of‐principle study provides key insights for designing **AZO**‐based photo‐pharmaceuticals with suitable molecular designs for bidirectional photoswitching within the red and NIR region at excitation intensities within the tolerance limits of animal tissue. While the generation of singlet oxygen remains an issue for in vivo applications of TSP, preliminary experiments performed in the presence of biomolecules present in vivo to address oxidative stress (serum albumin and glutathione) showed no interference with TSP, in addition to minimal change in the serum albumin structure. Additionally, we identified challenges associated with the photosensitizers for the application of triplet‐sensitized process for in vivo photopharmacology like aggregation, photobleaching and singlet oxygen generation, which needs to be addressed with a suitable molecular design, which is a work in progress in our lab. Although *trans‐to‐cis* isomerization has been achieved with redlight in this and other reported works, a low extinction coefficient at the tail end of the *n* − π* absorption bands remain an issue, which prolongs the *trans‐to‐cis* photo‐kinetics (≥ 30 min). While it may be useful in some pharmacological applications, many applications prefer fast molecular switching, hence our future efforts will be dedicated to develop strategies for chemical engineering of azobenzene‐based photopharmaceuticals to address this challenge. Moreover, for practical photopharmacological applications, we are now targeting to synthesize antiarrhythmic drugs such as sodium‐ or potassium‐ion channel blockers (bupivacaine and amiodarone) [70] with such design principles with some early success and detailed photopharmacological results upon excitation with red and far‐red light will be published soon.

## 5 Experimental Section/Methods

### 5.1 Materials

All solvents and reagents were used as received. All solvents used in this work were purchased from Sigma Aldrich. Pluronic F‐127, NaH_2_PO_4_, and Na_2_HPO_4_ were purchased from Sigma Aldrich. The azobenzene, (*E*)‐4‐((2,6‐dimethoxyphenyl)diazenyl)‐*N*‐(2‐ethylhexyl)‐3,5‐dimethoxyaniline (**AZO‐N**), was synthesized in the Kasper Moth‐Poulsen lab (II) as described below. 8,8,18,18‐Tetramethyl‐2,12‐di‐*p*‐tolyl‐bacteriochlorin (**BC1**), Zn‐2,3,9,10,16,17,23,24‐octakis(hexylthio)phthalocyanine (**ZnPcP**), Zn(II)‐1,4,8,11,15,18,22,25‐octakis((2‐ethylhexyl)thio)phthalocyanine (**ZnPcNP**), and Pd(II)‐1,4,8,11,15,18,22,25‐octabutoxy‐2,3,9,10,16,17,23,24‐octaphenoxyphthalocyanine (**PdPc1**) were synthesized as described in the literature [39, 47, 71, 72]. 1, 3‐diphenylisobenzofuran, bovine serum albumin, and glutathione were purchased from Sigma Aldrich.

### 5.2 Synthesis of **AZO‐N**

A new lipophilic tetra‐ortho‐methoxy azobenzene derivative (**AZO‐N**) has been synthesized in a two‐step reaction with a yield of 25% (see Scheme S1).

**Step 1**: Synthesis of (E)‐1,2‐bis(2,6‐dimethoxyphenyl)diazene (3, **mAzo**‐**NH_2_**).
A solution of 2,6‐dimethoxyaniline (0.459 g, 3.00 mmol) in a mixture of 0.56 mL of H_2_O and 0.73 mL HCl (37 wt %) was cooled to 0°C–5°C, followed by the slow addition of aqueous NaNO_2_ (0.207 g, 3.00 mmol in 2 mL of H_2_O). The resulting solution was stirred for 20 min at 0°C–5°C. The diazonium salt was then added slowly to an aqueous suspension of 3.5‐dimethoxyaniline (0.459 g, 3.00 mmol in 20 mL of H_2_O) at 0°C–5°C. The pH of the mixture was adjusted to 8–9 by adding saturated sodium bicarbonate solution, and then the mixture was stirred overnight. The red solid was filtered and purified by chromatography [silica, methanol/ethyl acetate (1:1)] to obtain the title compound (0.495 g, 52%) [73], followed by analysis by ^1^H NMR spectroscopy, ^13^C{^1^H} NMR spectroscopy, and HR‐MS (Figures S1 and S2)
^1^H NMR (300 MHz, CDCl_3_) δ 7.23 (t, *J* = 8.4 Hz, 1H), 6.68 (d, *J* = 8.5 Hz, 2H), 5.95 (dd, *J* = 27.0, 2.5 Hz, 2H), 3.96 (d, *J* = 4.4 Hz, 9H), 3.85 (s, 3H).
HR‐MS observed 318.1475, calculated 318.1449 [M + H)^+^], M = C_16_H_18_N_2_O_4_.
**Step 2**: Synthesis of 4 (E)‐4‐((2,6‐dimethoxyphenyl) diazenyl)‐N‐(2‐ethylhexyl)‐3,5‐dimethoxyaniline (4, trans‐**AZO‐N**).
A crude mixture of **mAzo‐NH_2_** (10.15 g, 32.0 mmol), 2‐ethylhexyl bromide (6.18 g, 32.0 mmol), and KHCO_3_ (4.4 g, 32.0 mmol) in 50 mL of acetonitrile was refluxed under nitrogen for 12 h. After evaporation of the volatile constituents, the resulting residue was extracted with CH_2_Cl_2_. The organic extract was washed with brine and then dried with anhydrous Na_2_SO_4_. The resulting liquid was purified by column chromatography [silica, ethyl acetate/hexanes (1:1)] to afford an orange solid (3.5 g, 25%). The solid was recrystallized [H_2_O: acetone] (1:1), followed by analysis by ^1^H NMR spectroscopy, ^13^C{^1^H} NMR spectroscopy, HR‐MS, and differential scanning calorimetry (Figures S3–S7).
*^1^H NMR* (300 MHz, CDCl_3_) δ 10.87 (s, 1H), 7.15 (t, *J* = 8.4 Hz, 1H), 6.67 (d, *J* = 8.4 Hz, 2H), 6.01–5.71 (m, 2H), 3.94 (s, 3H), 3.87 (d, *J* = 4.3 Hz, 9H), 3.17 (td, *J* = 5.6, 1.8 Hz, 2H), 1.69 (h, *J* = 6.0 Hz, 1H), 1.54–1.15 (m, 9H), 1.06–0.79 (m, 6H).
*^13^C{^1^H} NMR* (75 MHz, CDCl_3_) δ 153.17, 127.50, 105.36, 87.15, 86.74, 77.24, 56.47, 56.28, 55.19, 46.15, 38.67, 31.30, 29.01, 24.48, 23.04, 14.07, 10.90.
*HR‐MS* observed 430.2708, calculated 430.2701 [M + H)^+^], M = C_24_H_35_N_3_O_4_.
*Melting point* (T_m_) = 91 °C.

### 5.3 Preparation of samples for spectroscopy

#### 5.3.1 Preparation of the **AZO‐N** Solution in DMSO

A sample of 38 µL of the **AZO‐N** stock solution in toluene (2.8 mm) was placed in a glass vial and treated with a continuous flow of N_2_ to evaporate the toluene. The residual mass was treated with 3 mL of DMSO, followed by stirring until complete dissolution, affording a concentration of 36 µm.

#### 5.3.2 Preparation of the Photosensitizers (**BC1, ZnPcP, ZnPcNP**, and **PdPc1**) in Solutions of DMSO or Cyclohexane

A sample of 10 µL of the stock solution of **BC1** in toluene (500 µm) was placed in a glass vial and treated with a continuous flow of N_2_ to evaporate the toluene. The residual mass was treated with 3 mL of DMSO, followed by stirring until complete dissolution, affording a concentration of 1.66 µm.

A sample of 28 µL of the stock solution of **ZnPcP** in toluene (530.6 µm) was placed in a glass vial and treated with a continuous flow of N_2_ to evaporate the toluene. The residual mass was treated with 3 mL of DMSO, followed by stirring until complete dissolution, affording a concentration of 5 µm.

A sample of 35 µL of the stock solution of **ZnPcNP** in toluene (433 µm) was placed in a glass vial and treated with a continuous flow of N_2_ to evaporate the toluene. The residual mass was treated with 3 mL of DMSO, followed by stirring until complete dissolution, affording a concentration of 5 µm.

A sample of 20 µL of the stock solution of **PdPc1** in cyclohexane (200 µm) was taken in a glass vial, followed by further dilution with cyclohexane to give a total volume of 3 mL, affording a concentration of 1.33 µm.

Preparation of **AZO‐N** and photosensitizers (**BC1, ZnPcP, ZnPcNP**, and **PdPc1**) solutions in 2% **PF‐127** micelles in buffer solution pH 7.4: The aqueous solutions of chromophores in **PF‐127** micelles were prepared by treating 0.06 g of **PF‐127** in a glass vial with a specified volume of the stock solution of chromophores to achieve the desired concentration. Subsequent heating of the **PF‐127**‐chromophores solution at 80°C in an oven for 5 min caused melting of the **PF‐127** (T_m_ = 57°C). The viscous homogeneous solution was then purged with N_2_ to remove the organic solvent (toluene or cyclohexane). The heating and N_2_ purge cycle was repeated 4 times for complete removal of toluene or cyclohexane. The resulting solid sample of **PF‐127**‐chromophores at room temperature was treated with 3 mL of phosphate buffer solution (10 mm, pH 7.4), followed by magnetic stirring in an ice bath for 10–15 min for complete dissolution of **PF‐127**. The clear solution so‐obtained was annealed at 80°C in an oven for 5 min to enhance the crosslinking between ethylene oxide chains, thereby creating a barrier for oxygen entry into the chromophore region. The stock solutions of chromophores used were as follows

1. 38 µL of the **AZO‐N** stock solution in toluene (2.8 mm). Final Conc. = 36 µm
2. 10 µL of the **BC1** stock solution in toluene (500 µm). Final Conc. = 1.66 µm
3. 28 µL of the **ZnPcP** stock solution in toluene (530.6 µm). Final Conc. = 5 µm
4. 35 µL of the **ZnPcNP** stock solution in toluene (433 µm). Final Conc. = 5 µm
5. 20 µL of the **PdPc1** stock solution in cyclohexane (171 µm). Final Conc. = 1.2 µm

#### 5.3.3 Preparation of **AZO‐N**‐**BC1** Solution in DMSO

Samples of 38 µL of the **AZO‐N** stock solution in toluene (2.8 mm) and 10 µL of the stock solution of **BC1** in toluene (500 µm) were placed in a glass vial and treated with a continuous flow of N_2_ to remove the toluene. The residual mass was treated with 3 mL of DMSO and stirred for complete dissolution to afford a solution of **AZO‐N** (36 µm) and **BC1** (1.66 µm).

#### 5.3.4 Preparation of **AZO‐N‐ZnPcP** Solution in DMSO

Samples of 38 µL of the **AZO‐N** stock solution in toluene (2.8 mm) and 28 µL of the stock solution of **ZnPcP** in toluene (530.6 µm) were placed in a glass vial and treated with a continuous flow of N_2_ to remove the toluene. The residual mass was treated with 3 mL of DMSO and stirred for complete dissolution to afford a solution of **AZO‐N** (36 µm) and **ZnPcP** (5 µm).

#### 5.3.5 Preparation of **AZO‐N‐ZnPcP** Solution in Toluene

Samples of 38 µL of the **AZO‐N** stock solution in toluene (2.8 mm) and 28 µL of the stock solution of **ZnPcP** in toluene (530.6 µm) were placed in a glass vial and treated with toluene to give a volume of 3 mL with stirring for complete dissolution to afford a solution of **AZO‐N** (36 µm) and **ZnPcP** (5 µm).

#### 5.3.6 Preparation of **AZO‐N‐BC1** Solution in **PF‐127** Micelles in Phosphate Buffer pH 7.4

Sample of 0.06 g of **PF‐127** in a glass vial was treated with 38 µL of the **AZO‐N** stock solution in toluene (2.8 mm) and 10 µL of the stock solution of **BC1** in toluene (500 µm). Subsequent heating of the **PF‐127**‐chromophores solution at 80°C in an oven for 5 min caused melting of the **PF‐127** (T_m_ = 57°C). The viscous homogeneous solution was then purged with N_2_ to remove the toluene. The heating and N_2_ purge cycle was repeated 4 times for complete removal of toluene. The resulting solid sample of **PF‐127**‐chromophores at room temperature was treated with 3 mL of phosphate buffer solution (10 mm, pH 7.4), followed by magnetic stirring in an ice bath for 10–15 min for complete dissolution of **PF‐127**. The clear solution so‐obtained was annealed at 80°C in an oven for 5 min to enhance the crosslinking between ethylene oxide chains, thereby creating a barrier for oxygen entry into the chromophore region. The final concentrations were **AZO‐N** = 36 µm, **BC1** = 1.66 µm, and **PF‐127** = 2%.

#### 5.3.7 Preparation of **AZO‐N‐ZnPcNP** Solution in **PF‐127** Micelles in Phosphate Buffer pH 7.4

A sample of 0.06 g of **PF‐127** in a glass vial was treated with 38 µL of the **AZO‐N** stock solution in toluene (2.8 mm) and 35 µL of the **ZnPcNP** stock solution in toluene (433 µm). Subsequent heating of the **PF‐127**‐chromophores solution at 80°C in an oven for 5 min caused melting of the **PF‐127** (T_m_ = 57°C). The viscous homogeneous solution was then purged with N_2_ to remove the toluene. The heating and N_2_ purge cycle was repeated 4 times for complete removal of toluene. The resulting solid sample of **PF‐127**‐chromophores at room temperature was treated with 3 mL of phosphate buffer solution (10 mm, pH 7.4), followed by magnetic stirring in an ice bath for 10–15 min for complete dissolution of **PF‐127**. The clear solution so‐obtained was annealed at 80°C in an oven for 5 min to enhance the crosslinking between ethylene oxide chains, thereby creating a barrier for oxygen entry into the chromophore region. The final concentrations were **AZO‐N** = 36 µm, **ZnPcNP** = 5 µm, and **PF‐127** = 2%.

#### 5.3.8 Preparation of **AZO‐N‐PdPc1** Solution in **PF‐127** Micelles in Phosphate Buffer pH 7.4

A sample of 0.21 g of **PF‐127** in a glass vial, was treated with 50 µL of the **AZO‐N** stock solution in toluene (3.88 mm) and 20 µL of the stock solution of **PdPc1** in cyclohexane (171 µm). Subsequent heating of the **PF‐127**‐chromophores solution at 80°C in an oven for 5 min caused melting of the **PF‐127** (T_m_ = 57°C). The viscous homogeneous solution was then purged with N_2_ to remove the cyclohexane. The heating and N_2_ purge cycle was repeated 4 times for complete removal of cyclohexane. The resulting solid sample of **PF‐127**‐chromophores at room temperature was treated with 3 mL of phosphate buffer solution (10 mm, pH 7.4), followed by magnetic stirring in an ice bath for 10–15 min for complete dissolution of **PF‐127**. The clear solution so‐obtained was annealed at 80°C in an oven for 5 min to enhance the crosslinking between ethylene oxide chains, thereby creating a barrier for oxygen entry into the chromophore region. The final concentrations were **AZO‐N** = 64.6 µm, **PdPc1** = 1.2 µm, and **PF‐127** = 7%.

#### 5.3.9 Preparation of **DPBF**‐Toluene

38.56 µL of **DPBF** in toluene (3.89 mm) was diluted to 3 mL in toluene, resulting in the final concentration of 50 µm.

#### 5.3.10 Preparation of **DPBF‐AZO‐N‐PdPc1**‐Toluene solution

30 µL of **PdPc1** in toluene (207 µm), and 38.56 µL of **DPBF** in toluene (3.89 mm) and 45 µL of **AZO‐N** (2.55 mm) were diluted to 3 mL in toluene, resulting in final concentrations, **AZO‐N** = 38 µm, **DPBF** = 50 µm, P**dPc1** = 2 µm.

#### 5.3.11 Preparation of **DPBF‐PdPc1**‐**PF‐127** Micelles in Phosphate Buffer pH 7.4

15 µL of **PdPc1** in toluene (207 µm), and 38.56 µL of **DPBF** in toluene (3.89 mm) were added in a glass vial containing 0.06 g of **PF‐127**. The vial containing these residues was kept at 80 °C for 5 min in a hot oven, followed by purging with N_2_ to evaporate toluene. The remaining solid residue was diluted with 3 mL of phosphate buffer solution (pH 7.4), followed by magnetic stirring in an ice bath for 10–15 min for complete solubilization of **PF‐127**. Final concentrations were **DPBF** = 50 µm, **P** **dPc1** = 1 µm, **PF‐127** = 2%.

#### 5.3.12 Preparation of **AZO‐N‐PdPc1‐BSA**‐Glutathione‐**PF‐127** Micelles in Phosphate Buffer pH 7.4

15 µL of **PdPc1** in toluene (207 µm), 90 µL of **AZO‐N** (2.55 mm) were added in a glass vial containing 0.06 g of **PF‐127**. The vial containing these residues was kept at 80 °C for 5 min in a hot oven, followed by purging with N_2_ to evaporate toluene. The remaining residue was diluted with 2.83 mL of phosphate buffer solution and 170 µL of glutathione (17.6 mm), followed by magnetic stirring in the ice bath. To the resulting solution, added 100 mg of **BSA**, followed by magnetic stirring at room temperature for complete solubilization of BSA. Final concentrations were **P** **dPc1** = 1 µm, **PF‐127** = 2%, **BSA** = 500 µm, Glutathione = 1 mm, **AZO‐N** = 76 µm

### 5.4 Preparation of BSA Samples for Circular Dichroism Spectra Measurements

Three stock solutions of **BSA** (500 µm) were prepared either in phosphate buffer solution (I), **PF‐127**+Glutathione+ phosphate buffer solution (II), and **AZO‐N**+**PdPc1**+**PF‐127**+Glutathione + phosphate buffer solution (III). For this 100 mg of **BSA** was dissolved in phosphate buffer solutions containing different components in various amounts as indicated in section 5.3.12. These samples were diluted 100 times with PBS before CD measurements. Sample IV is sample III after 730 nm irradiation for 80 min. Final concentration of **BSA** in all of the sample solutions was 5 µm. The measurements were carried out in 0.1 cm pathlength quartz cuvette.

### 5.5 Optical Measurements

UV–vis measurements of the chromophore solutions were done using a UV–vis–NIR spectrophotometer (Jasco V‐780 with operational range of 190–3300 nm). Measurements were carried out in the dark room to avoid the interference of day light during photoswitching experiments. Samples were excited with 340, 455, 625, and 730 nm, LEDs Thorlabs integrated with collimator. LED emission power was measured with standard Photodiode Power Sensor Thor‐Labs S120VC. The emission spectra of LEDs used in these measurements are provided as Figure S14. Solution state spectra were recorded in a quartz cuvette of 1 cm pathlength. The absorption spectra of *cis*‐**AZO‐N** in Figure 2c were normalized with respect to absorbance of *trans*‐**AZO‐N** at 318 nm (highest absorbance), so that photoswitched spectra of *cis*‐**AZO‐N** are not altered with respect to *trans*‐**AZO‐N**.

Time‐resolved phosphorescence measurements were carried out using a modified Fluotime 200 fluorescence lifetime system from Picoquant [74]. A 664‐nm pulsed laser working at 2 kHz repetition rate (PULSELAS‐A‐660‐50 from ALPHALAS GmbH, Göttingen, Germany) was used for excitation, and a near‐IR sensitive photomultiplier (H10330C‐45‐C3 from Hamamatsu Photonics, Japan) was used for detection in photon counting mode. Spectral resolution of the emission was achieved using bandpass filters (1110, 1220, 1275, and 1320 nm). The system was controlled by the Picoquant software TimeHarp 260 version 3.2.0.0, and the phosphorescence decay kinetics were analyzed using the Picoquant EasyTau 2 version 2.2 software. All time‐resolved measurements were carried out in a 1 cm pathlength quartz cuvette at 295 K. Circular dichroism spectra were recorded using Jasco‐815‐1505 instrument in the far‐UV region (300–180 nm) with a scanning speed of 200 nm min^−1^, bandwidth of 1 nm with continuous N_2_ flow. BSA samples were diluted to 5 µm so that HT [V] comes down below 800 to ascertain the accuracy of measurements. All chromophores solution in organic solvents were degassed with a flow of N_2_ or argon for 30 min each, whereas the measurements of the aqueous solutions of chromophores in **PF‐127** were carried out in air‐equilibrated conditions.

### 5.6 Other Measurements

#### 5.6.1 ^1^H NMR and ^13^C {^1^H} NMR Spectroscopy

The ^1^H NMR and ^13^C {^1^H} NMR spectra were acquired using a Bruker 300 MHz NMR spectrometer including a Nanobay AVANCE nanoNEO console, ultra‐shielded ASCEND magnet, and a BBFO probe head. The ^1^H NMR spectra for the measurement of % cis and trans isomers in the photostationary state were recorded using a Bruker NMR Ascend 400 of 400 MHz equipped with two probes for liquid and solid samples.

#### 5.6.2 Differential Scanning Calorimetry (DSC)

DSC of **AZO‐N** was performed with a NETZSCH‐STA 449 F1 Jupiter, which allows for simultaneous TGA and differential scanning calorimetry/differential thermal analysis. Samples were analyzed with a scanning rate of 2°C min^−1^ from 25°C to 130°C under N_2_ flow. HR‐MS spectra were acquired using an Agilent LC/MSD TOF mass spectrometer by electrospray ionization time‐of‐flight (ESI‐TOF) reflection experiments. Dynamic light scattering measurements were performed in triplicate using Zetasizer Nano ZS, Malvern using 1 cm pathlength quartz cuvette.

#### 5.6.3 Computational Calculation of the Triplet Energies of Photosensitizers and cis or trans **AZO‐N**

Full geometry optimizations have been carried out with the M06‐2X density functional [50], with the 6–311G(d,p) basis set [51] and the Gaussian 16 program [75]. We used this method based on calculations performed in the systems of Figure S5. For all species, we have analyzed the lowest‐lying closed‐shell singlet ground state (S_0_) and the lowest‐lying triplet excited states (T_1_). For the latter, the geometry optimizations were performed within the unrestricted methodology, while for the former the restricted formalism was used. All optimized stationary points were verified by performing a vibrational analysis calculation to be energy minima (no imaginary frequencies). Except otherwise noted, T_1_ energies are adiabatic, i.e., energy differences between the optimized S_0_ and T_1_ structures in the gas‐phase. The calculation of the **ZnPc** system was performed in a model system in which we changed the hexyl by methyl groups.

## Conflicts of Interest

The authors declare no conflicts of interest.

## Supporting information




**Supporting File**: advs75155‐sup‐0001‐SuppMat.docx.

## Data Availability

The data that supports the findings of this study are available in the supplementary material of this article.
