# Supplementary material for: Bidirectional Photoswitching of a Tailored Azobenzene with Red and Far‐Red Light Involving Triplet Sensitization in an Aqueous System
Source: Adv Sci (Weinh). 2026 Apr 23;13(36):e75155. doi: 10.1002/advs.75155 (PMC13317749; doi:10.1002/advs.75155)
Supplement: Supplementary file 1 — Supporting File: advs75155‐sup‐0001‐SuppMat.docx. [file ADVS-13-e75155-s001.docx]

Supporting Information

Bidirectional Photoswitching of a Tailored Azobenzene with Red and Far-red Light involving Triplet Sensitization in an Aqueous System

*Mila Miroshnichenko,^a^ Helen Hölzel,^b^ Edvinas Orentas,^c^ Karolis Kazlauskas,^d^ Pedro Ferreira,^b^ Fabienne Dumoulin,^e,f^ Carles Alcaide,^g^ Miquel Sola,^g^ Roger Bresolí-Obach,^h^ Santi Nonell,^h,i,^ Pankaj Bharmoria^a,b*^, Kasper Moth-Poulsen^a,b,i,j*^*

**Table of Contents**

**Topic Page**

1. Experimental section (organic synthesis and characterization) 1-5

2. Computational calculations 6-20

3. Supporting figures 20-45

4. Supporting Tables 26, 32, 35, 38

5. Supporting references 45

**1. Experimental section**

**Synthesis of AZO-N**

**Scheme S1.** Illustration of the synthesis scheme of *trans*-**AZO-N**

**Figure S1.** ^1^H NMR spectrum of **mAzo-NH_2_**

^1^H NMR (300 MHz, CDCl_3_) δ 7.23 (t, *J* = 8.4 Hz, 1H), 6.68 (d, *J* = 8.5 Hz, 2H), 5.95 (dd, *J* = 27.0, 2.5 Hz, 2H), 3.96 (d, *J* = 4.4 Hz, 9H), 3.85 (s, 3H).


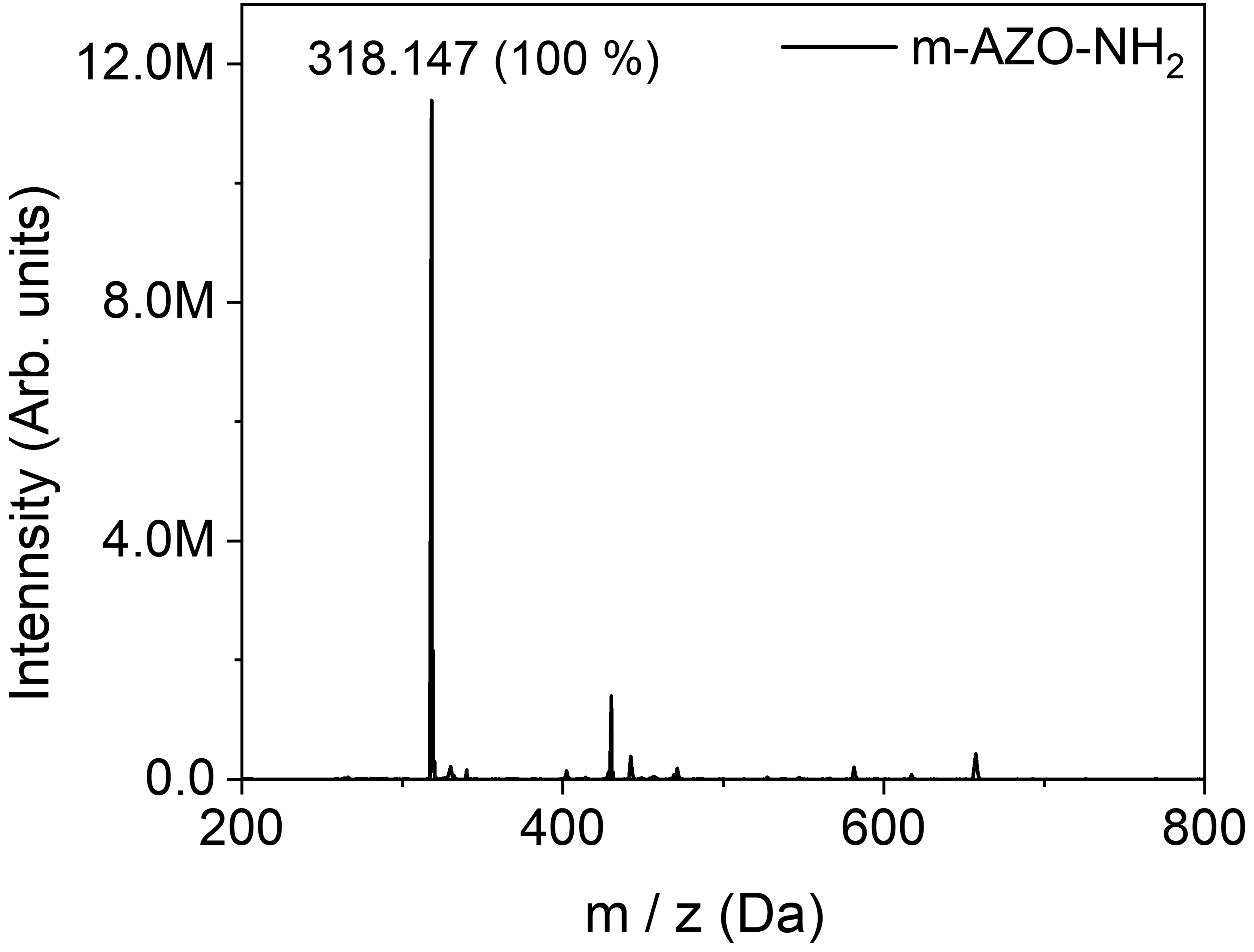


**Figure S2.** HR-MS spectra of **mAzo-NH_2_**

HR-MS observed 318.1475, calculated 318.1449 [M + H)^+^], M = C_16_H_18_N_2_O_4_.

**Figure S3.** ^1^H NMR spectrum of ***trans-*AZO-N**

^1^H NMR (300 MHz, CDCl_3_) δ 10.87 (s, 1H), 7.15 (t, *J* = 8.4 Hz, 1H), 6.67 (d, *J* = 8.4 Hz, 2H), 6.01 – 5.71 (m, 2H), 3.94 (s, 3H), 3.87 (d, *J* = 4.3 Hz, 9H), 3.17 (td, *J* = 5.6, 1.8 Hz, 2H), 1.69 (h, *J* = 6.0 Hz, 1H), 1.54 – 1.15 (m, 9H), 1.06 – 0.79 (m, 6H).

**Figure S4.** ^13^ C{^1^H} NMR spectrum of *trans-***AZO-N**

^13^C{^1^H} NMR (75 MHz, CDCl_3_) δ 153.17, 127.50, 105.36, 87.15, 86.74, 77.24, 56.47, 56.28, 55.19, 46.15, 38.67, 31.30, 29.01, 24.48, 23.04, 14.07, 10.90.


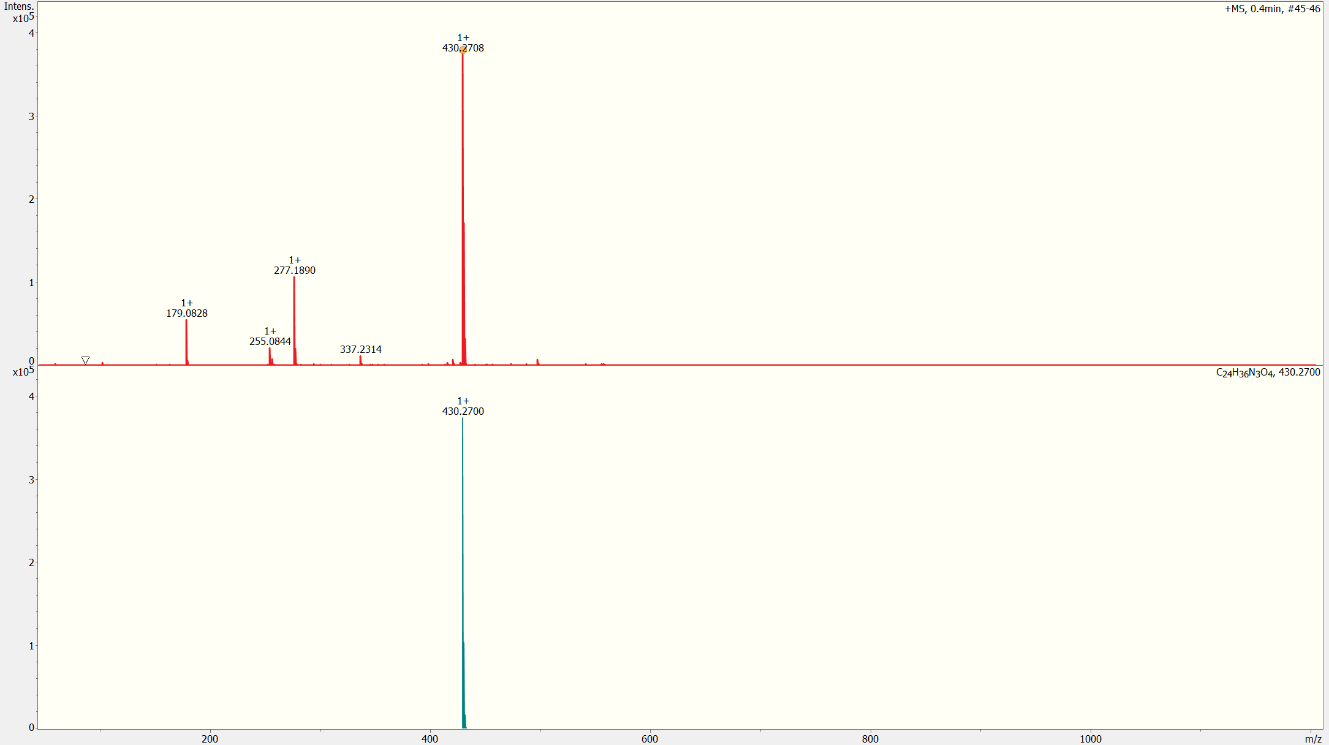


Experimental

Calculated

**Figure S5.** HR-MS spectrum of ***trans-*AZO-N**

HR-MS observed 430.2708, calculated 430.2701 [M + H)^+^], M = C_24_H_35_N_3_O_4_.


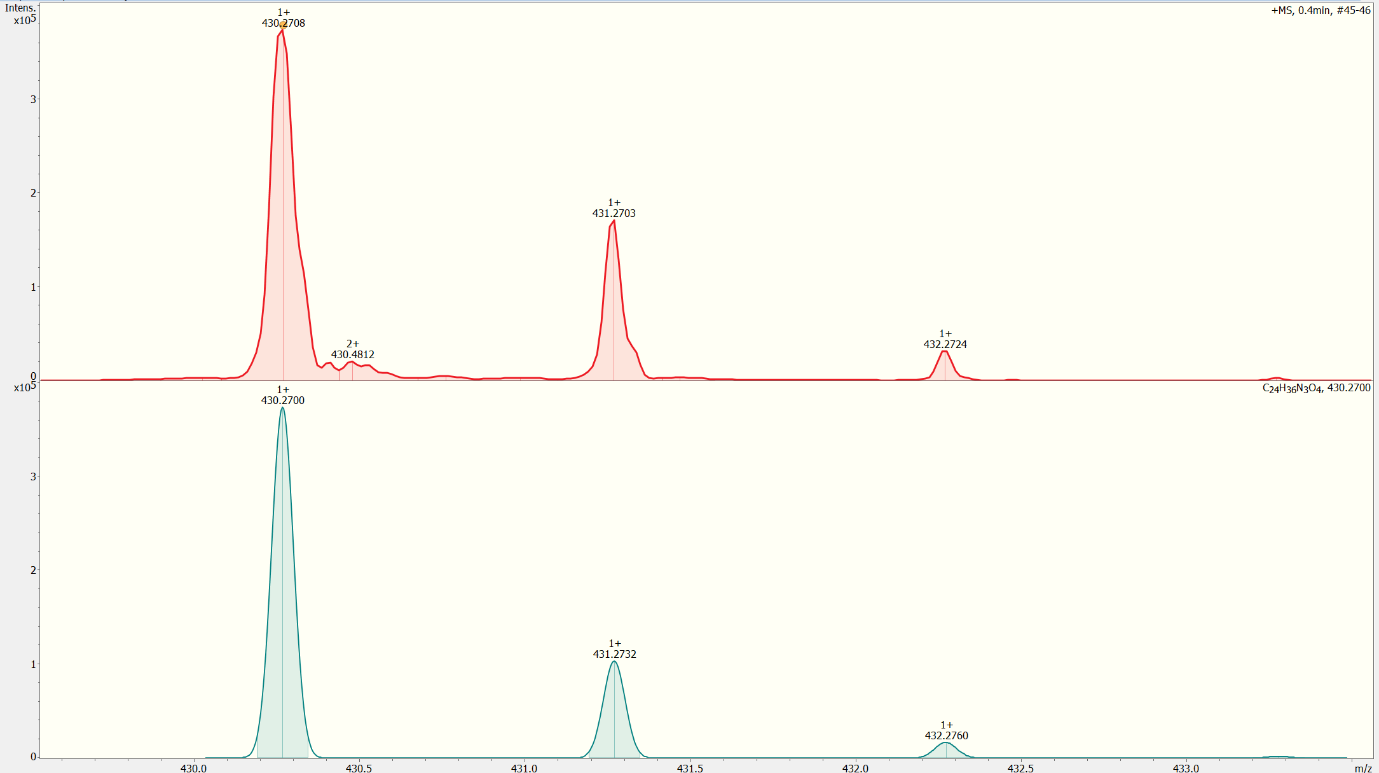


Experimental

Calculated

**Figure S6.** Enlarged HR-MS spectrum of ***trans-*AZO-N**

HR-MS observed 430.2708, calculated 430.2701 [M + H)^+^], M = C_24_H_35_N_3_O_4_.


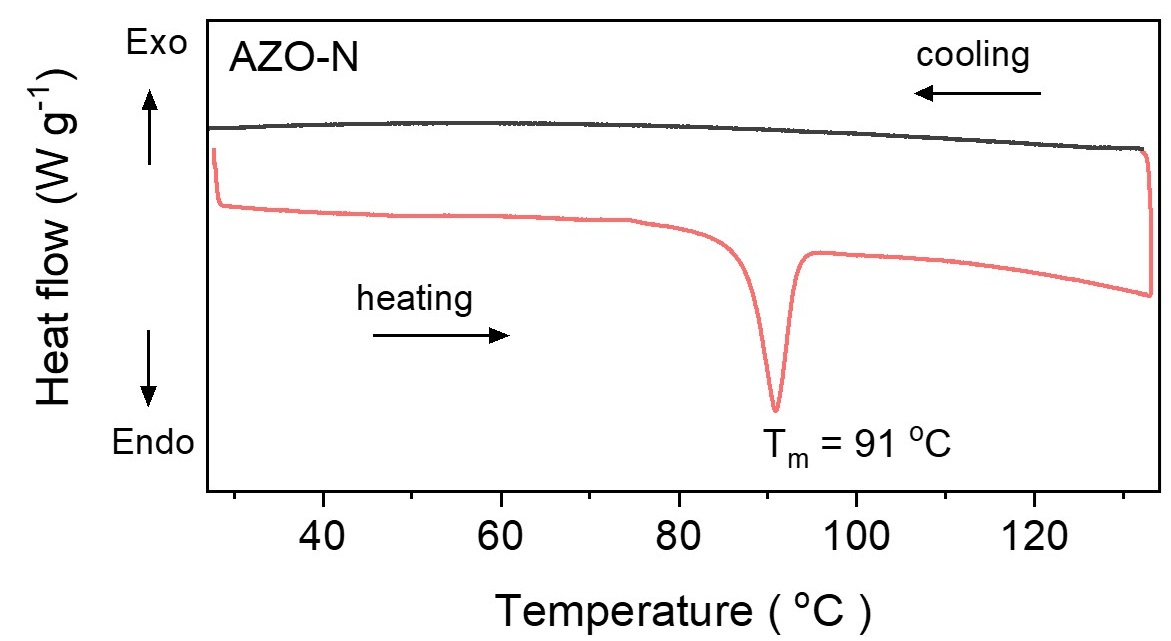


**Figure S7.** Differential scanning calorimetry thermogram of **AZO-N**


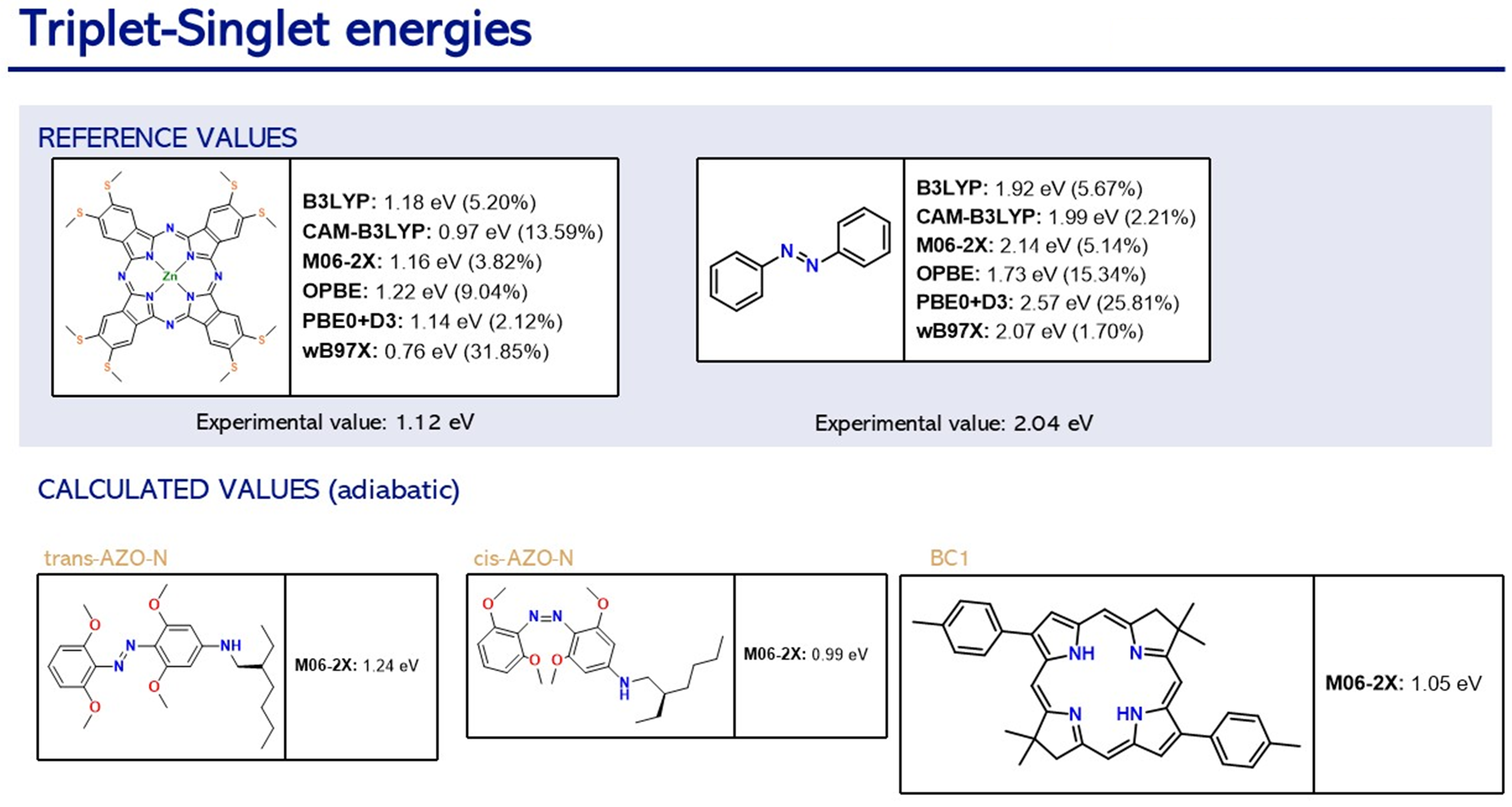


**Figure S8.** Calculated T_1_ energy values obtained with different functionals in conjunction with the 6-311G(d,p) basis set for **ZnPc, BC1, E-azobenzene and** *trans*-**AZO-N** or *cis*-**AZO-N** systems. In parenthesis, the percentage of error with respect to experimental values obtained from ref.1, 2

**2. Cartesian coordinates of all species studied**

**Bacteriochlorin (BC1)**

**Singlet (S_0_)**

6 -2.227837000 2.111427000 0.064451000

7 -1.942920000 0.778252000 0.018711000

6 -3.102417000 0.017335000 -0.002856000

6 0.957535000 4.277557000 0.164515000

6 2.306406000 3.632839000 0.139033000

6 2.136337000 2.294277000 0.087337000

7 0.762380000 1.919723000 0.074741000

6 0.079003000 3.031878000 0.114907000

6 -0.957526000 -4.277595000 0.164555000

6 -2.306399000 -3.632873000 0.139101000

6 -2.136327000 -2.294315000 0.087368000

7 -0.762371000 -1.919768000 0.074667000

6 -0.078994000 -3.031924000 0.114833000

6 4.213813000 -0.982003000 -0.016179000

6 3.679529000 -2.220903000 0.040798000

6 2.227845000 -2.111471000 0.064292000

7 1.942923000 -0.778295000 0.018583000

6 3.102416000 -0.017375000 -0.002952000

6 -4.213809000 0.981967000 -0.016059000

6 -3.679520000 2.220862000 0.040969000

6 -1.343298000 3.150665000 0.113101000

6 1.343308000 -3.150709000 0.112947000

6 3.208457000 1.327410000 0.044314000

6 -3.208451000 -1.327450000 0.044369000

1 -1.010446000 0.389543000 0.052933000

1 3.248333000 4.163409000 0.159868000

1 -3.248328000 -4.163436000 0.160009000

1 1.010446000 -0.389599000 0.052863000

1 -1.765975000 4.146357000 0.149287000

1 1.765988000 -4.146401000 0.149092000

1 4.211579000 1.735038000 0.067955000

1 -4.211569000 -1.735087000 0.067983000

6 0.717704000 5.066892000 1.462228000

1 0.862705000 4.427225000 2.334430000

1 1.417146000 5.904125000 1.523713000

1 -0.298852000 5.467393000 1.489848000

6 0.719151000 5.169901000 -1.064666000

1 -0.296799000 5.572767000 -1.060141000

1 1.419529000 6.008612000 -1.057885000

1 0.863172000 4.603088000 -1.986061000

6 -0.719214000 -5.170087000 -1.064513000

1 -0.863215000 -4.603399000 -1.985987000

1 0.296711000 -5.573017000 -1.059949000

1 -1.419638000 -6.008760000 -1.057610000

6 -0.717617000 -5.066850000 1.462302000

1 -0.862630000 -4.427185000 2.334499000

1 -1.417002000 -5.904131000 1.523809000

1 0.298966000 -5.467286000 1.489883000

1 -4.212677000 3.160266000 0.030079000

1 4.212687000 -3.160305000 0.029871000

6 5.642549000 -0.637940000 -0.080978000

6 6.128446000 0.272146000 -1.024911000

6 6.553992000 -1.256637000 0.777758000

6 7.485658000 0.552584000 -1.099856000

6 7.909725000 -0.969553000 0.697419000

6 8.398047000 -0.058227000 -0.238620000

1 5.438898000 0.737025000 -1.720154000

1 6.188109000 -1.955729000 1.521328000

1 7.845731000 1.252001000 -1.847143000

1 8.601170000 -1.458070000 1.375935000

6 -5.642548000 0.637934000 -0.080904000

6 -6.554018000 1.256694000 0.777778000

6 -6.128433000 -0.272147000 -1.024831000

6 -7.909748000 0.969683000 0.697362000

6 -7.485665000 -0.552526000 -1.099848000

6 -8.398067000 0.058353000 -0.238699000

1 -6.188125000 1.955778000 1.521351000

1 -5.438883000 -0.737086000 -1.720031000

1 -8.601223000 1.458242000 1.375817000

1 -7.845711000 -1.251946000 -1.847147000

6 -9.864954000 -0.278166000 -0.301947000

1 -10.475866000 0.552671000 0.053689000

1 -10.170314000 -0.517296000 -1.321759000

1 -10.087969000 -1.146993000 0.323612000

6 9.864915000 0.278410000 -0.301769000

1 10.170222000 0.518080000 -1.321466000

1 10.087899000 1.146916000 0.324247000

1 10.475885000 -0.552578000 0.053423000

**Triplet (T_1_)**

6 2.262219000 -2.110124000 0.099929000

7 1.979096000 -0.768403000 0.083068000

6 3.131341000 -0.039844000 0.026998000

6 -0.962808000 -4.299278000 0.186539000

6 -2.299655000 -3.637822000 0.147901000

6 -2.117434000 -2.276461000 0.091129000

7 -0.782008000 -1.915216000 0.088417000

6 -0.071217000 -3.058450000 0.138284000

6 0.942596000 4.302044000 0.196213000

6 2.294626000 3.675125000 0.190603000

6 2.144294000 2.281330000 0.121632000

7 0.774723000 1.928529000 0.087618000

6 0.084422000 3.039203000 0.125092000

6 -4.208180000 0.981417000 -0.038075000

6 -3.669199000 2.220294000 0.019666000

6 -2.221389000 2.104888000 0.049480000

7 -1.939651000 0.770534000 0.007406000

6 -3.099729000 0.018270000 -0.018437000

6 4.209822000 -0.954014000 -0.012899000

6 3.653897000 -2.238483000 0.048908000

6 1.305184000 -3.154289000 0.141424000

6 -1.333894000 3.144156000 0.104942000

6 -3.201399000 -1.330568000 0.033978000

6 3.194311000 1.380042000 0.085182000

1 1.052344000 -0.366402000 0.134882000

1 -3.249395000 -4.155622000 0.161861000

1 3.232015000 4.211447000 0.230080000

1 -1.008586000 0.375758000 0.032221000

1 1.729379000 -4.152389000 0.173074000

1 -1.762174000 4.137878000 0.138616000

1 -4.201289000 -1.745340000 0.053545000

1 4.191394000 1.799792000 0.130204000

6 -0.756655000 -5.095322000 1.487432000

1 -0.893399000 -4.453080000 2.358898000

1 -1.472701000 -5.919186000 1.542909000

1 0.251490000 -5.515718000 1.522043000

6 -0.740888000 -5.208537000 -1.034920000

1 0.266420000 -5.631959000 -1.018643000

1 -1.457836000 -6.033451000 -1.026095000

1 -0.864209000 -4.646793000 -1.962230000

6 0.713937000 5.201496000 -1.031500000

1 0.875909000 4.643174000 -1.955134000

1 -0.304161000 5.598854000 -1.039565000

1 1.408375000 6.044827000 -1.007984000

6 0.671092000 5.086940000 1.492023000

1 0.802321000 4.446789000 2.366095000

1 1.365517000 5.927108000 1.568673000

1 -0.346982000 5.484360000 1.501351000

1 4.189858000 -3.175093000 0.013966000

1 -4.198941000 3.161532000 0.007519000

6 -5.636820000 0.638379000 -0.105154000

6 -6.120347000 -0.276933000 -1.045217000

6 -6.550108000 1.262201000 0.747972000

6 -7.477322000 -0.558153000 -1.121507000

6 -7.905475000 0.974395000 0.666137000

6 -8.391615000 0.057444000 -0.265722000

1 -5.429649000 -0.744712000 -1.737466000

1 -6.186296000 1.965534000 1.488563000

1 -7.835690000 -1.261391000 -1.865963000

1 -8.598511000 1.466746000 1.340170000

6 5.639980000 -0.629266000 -0.101509000

6 6.570891000 -1.322136000 0.679813000

6 6.121737000 0.340442000 -0.987505000

6 7.927716000 -1.047329000 0.585323000

6 7.480252000 0.616665000 -1.071333000

6 8.406696000 -0.068143000 -0.285640000

1 6.216870000 -2.073520000 1.377068000

1 5.424504000 0.857152000 -1.637532000

1 8.629379000 -1.598162000 1.203585000

1 7.829716000 1.367789000 -1.772474000

6 9.876825000 0.254213000 -0.357263000

1 10.484511000 -0.630975000 -0.162229000

1 10.147067000 0.644902000 -1.339552000

1 10.144881000 1.010375000 0.386166000

6 -9.858283000 -0.279311000 -0.330283000

1 -10.160487000 -0.528490000 -1.348570000

1 -10.083226000 -1.141814000 0.303255000

1 -10.470067000 0.555065000 0.015314000

**Zn-Phthalocyanine (ZnPc)**

**Singlet (S_0_)**

7 -1.854297000 0.820885000 -0.049840000

7 0.766587000 1.857324000 -0.039591000

7 1.806491000 -0.763758000 -0.017574000

7 -0.818145000 -1.801987000 -0.027505000

7 -1.264110000 3.162362000 -0.059856000

7 3.108536000 1.270132000 -0.024717000

7 1.214902000 -3.105359000 -0.005574000

7 -3.159580000 -1.209939000 -0.043434000

6 -2.125764000 2.154625000 -0.058304000

6 2.102451000 2.130091000 -0.035485000

6 2.078704000 -2.100354000 -0.008074000

6 -2.151465000 -2.074461000 -0.032634000

6 -3.015903000 0.105660000 -0.051832000

6 0.054573000 3.019325000 -0.052455000

6 2.965026000 -0.050614000 -0.016241000

6 -0.102974000 -2.963214000 -0.013859000

6 -3.577421000 2.329166000 -0.066266000

6 2.276140000 3.583089000 -0.046086000

6 3.528595000 -2.272610000 -0.000214000

6 -2.325634000 -3.524112000 -0.022668000

6 -4.134280000 1.047216000 -0.063845000

6 0.993449000 4.136169000 -0.056977000

6 4.083547000 -0.988922000 -0.004188000

6 -1.042735000 -4.080606000 -0.009373000

6 -4.382836000 3.460707000 -0.085796000

6 3.415756000 4.374509000 -0.052992000

6 4.337995000 -3.402417000 0.019850000

6 -3.457104000 -4.331272000 -0.013747000

6 -5.511224000 0.864284000 -0.062114000

6 0.820969000 5.515225000 -0.064277000

6 5.460968000 -0.804077000 -0.007923000

6 -0.859715000 -5.458332000 -0.005870000

6 -5.764768000 3.292987000 -0.097257000

6 3.255769000 5.759844000 -0.071235000

6 5.718861000 -3.231796000 0.030220000

6 -3.288679000 -5.712303000 0.003390000

6 -6.331440000 1.989220000 -0.068560000

6 1.948521000 6.325139000 -0.064002000

6 6.283327000 -1.926255000 -0.000721000

6 -1.983630000 -6.278636000 -0.009467000

1 -3.950001000 4.453204000 -0.103886000

1 4.387749000 3.901070000 -0.054412000

1 3.907765000 -4.396046000 0.038715000

1 -4.450135000 -3.899299000 -0.008859000

1 -5.942023000 -0.129111000 -0.045081000

1 -0.167953000 5.956900000 -0.067345000

1 5.889842000 0.190012000 -0.027666000

1 0.133949000 -5.888803000 -0.011977000

30 -0.024509000 0.027611000 -0.034742000

16 -8.097546000 1.715678000 -0.069864000

16 -6.769684000 4.770749000 -0.121004000

6 -8.477174000 2.190215000 1.646024000

1 -9.544201000 2.017242000 1.782605000

1 -7.916622000 1.565147000 2.338771000

1 -8.255617000 3.243942000 1.803924000

6 -7.341156000 4.718029000 -1.848484000

1 -7.939051000 5.616020000 -2.000886000

1 -6.487923000 4.731134000 -2.524224000

1 -7.957743000 3.836872000 -2.015020000

16 -1.709674000 -8.044634000 -0.001076000

16 -4.765763000 -6.718453000 0.012515000

16 6.727472000 -4.707026000 0.054482000

16 8.048845000 -1.649091000 -0.002138000

16 4.623803000 6.880469000 -0.119384000

16 1.720659000 8.095657000 -0.088491000

6 2.002751000 8.444856000 1.677936000

1 1.258708000 7.933198000 2.285726000

1 3.008590000 8.147245000 1.970439000

1 1.895580000 9.522048000 1.800918000

6 6.034701000 5.754377000 -0.003360000

1 6.007706000 5.173177000 0.917983000

1 6.092399000 5.098102000 -0.871079000

1 6.912728000 6.398573000 0.008963000

6 -4.731708000 -7.287604000 1.741151000

1 -3.852316000 -7.903810000 1.918159000

1 -4.752102000 -6.433482000 2.415643000

1 -5.631189000 -7.885468000 1.884762000

6 -2.162656000 -8.428147000 -1.721931000

1 -3.214184000 -8.206295000 -1.893593000

1 -1.528619000 -7.869331000 -2.407896000

1 -1.988572000 -9.495560000 -1.854049000

6 8.428519000 -2.125774000 -1.717455000

1 9.494893000 -1.950146000 -1.855830000

1 8.209829000 -3.180511000 -1.872513000

1 7.864925000 -1.504392000 -2.411058000

6 7.300032000 -4.651382000 1.781434000

1 7.900271000 -5.547718000 1.934421000

1 7.914541000 -3.768548000 1.946949000

1 6.447263000 -4.665964000 2.457767000

**Triplet (T_1_)**

7 1.854298000 -0.820883000 -0.049840000

7 -0.766585000 -1.857325000 -0.039591000

7 -1.806492000 0.763756000 -0.017574000

7 0.818143000 1.801988000 -0.027505000

7 1.264113000 -3.162361000 -0.059856000

7 -3.108535000 -1.270135000 -0.024717000

7 -1.214905000 3.105358000 -0.005574000

7 3.159579000 1.209942000 -0.043434000

6 2.125766000 -2.154623000 -0.058304000

6 -2.102449000 -2.130093000 -0.035485000

6 -2.078706000 2.100352000 -0.008074000

6 2.151463000 2.074463000 -0.032634000

6 3.015903000 -0.105657000 -0.051832000

6 -0.054570000 -3.019325000 -0.052455000

6 -2.965026000 0.050611000 -0.016241000

6 0.102971000 2.963214000 -0.013859000

6 3.577423000 -2.329163000 -0.066266000

6 -2.276137000 -3.583091000 -0.046086000

6 -3.528597000 2.272607000 -0.000214000

6 2.325631000 3.524114000 -0.022668000

6 4.134281000 -1.047212000 -0.063845000

6 -0.993445000 -4.136170000 -0.056977000

6 -4.083548000 0.988918000 -0.004188000

6 1.042731000 4.080607000 -0.009373000

6 4.382839000 -3.460703000 -0.085796000

6 -3.415752000 -4.374512000 -0.052992000

6 -4.337998000 3.402413000 0.019850000

6 3.457100000 4.331275000 -0.013747000

6 5.511225000 -0.864279000 -0.062114000

6 -0.820964000 -5.515226000 -0.064277000

6 -5.460969000 0.804072000 -0.007923000

6 0.859710000 5.458333000 -0.005870000

6 5.764771000 -3.292982000 -0.097257000

6 -3.255764000 -5.759847000 -0.071235000

6 -5.718864000 3.231791000 0.030220000

6 3.288674000 5.712306000 0.003390000

6 6.331442000 -1.989214000 -0.068560000

6 -1.948515000 -6.325141000 -0.064002000

6 -6.283329000 1.926249000 -0.000721000

6 1.983624000 6.278638000 -0.009467000

1 3.950005000 -4.453200000 -0.103886000

1 -4.387745000 -3.901074000 -0.054412000

1 -3.907769000 4.396042000 0.038715000

1 4.450131000 3.899303000 -0.008859000

1 5.942023000 0.129117000 -0.045081000

1 0.167959000 -5.956900000 -0.067345000

1 -5.889842000 -0.190017000 -0.027666000

1 -0.133954000 5.888803000 -0.011977000

30 0.024509000 -0.027611000 -0.034742000

16 8.097548000 -1.715670000 -0.069864000

16 6.769689000 -4.770743000 -0.121004000

6 8.477176000 -2.190207000 1.646024000

1 9.544203000 -2.017233000 1.782605000

1 7.916624000 -1.565140000 2.338771000

1 8.255620000 -3.243934000 1.803924000

6 7.341160000 -4.718022000 -1.848484000

1 7.939056000 -5.616013000 -2.000886000

1 6.487927000 -4.731128000 -2.524224000

1 7.957747000 -3.836865000 -2.015020000

16 1.709667000 8.044636000 -0.001076000

16 4.765757000 6.718457000 0.012515000

16 -6.727476000 4.707020000 0.054482000

16 -8.048846000 1.649083000 -0.002138000

16 -4.623797000 -6.880473000 -0.119384000

16 -1.720651000 -8.095659000 -0.088491000

6 -2.002743000 -8.444858000 1.677936000

1 -1.258701000 -7.933199000 2.285726000

1 -3.008582000 -8.147248000 1.970439000

1 -1.895571000 -9.522050000 1.800918000

6 -6.034696000 -5.754383000 -0.003360000

1 -6.007701000 -5.173183000 0.917983000

1 -6.092394000 -5.098108000 -0.871079000

1 -6.912722000 -6.398579000 0.008963000

6 4.731701000 7.287608000 1.741151000

1 3.852309000 7.903814000 1.918159000

1 4.752096000 6.433486000 2.415643000

1 5.631182000 7.885473000 1.884762000

6 2.162648000 8.428149000 -1.721931000

1 3.214176000 8.206298000 -1.893593000

1 1.528612000 7.869332000 -2.407896000

1 1.988563000 9.495562000 -1.854049000

6 -8.428521000 2.125766000 -1.717455000

1 -9.494895000 1.950137000 -1.855830000

1 -8.209832000 3.180503000 -1.872513000

1 -7.864926000 1.504385000 -2.411058000

6 -7.300036000 4.651375000 1.781434000

1 -7.900276000 5.547711000 1.934421000

1 -7.914544000 3.768541000 1.946949000

1 -6.447267000 4.665958000 2.457767000

***Trans*-AZO-N**

**Singlet (S_0_)**

7 2.920246000 -0.614984000 0.172570000

7 2.215191000 0.298550000 -0.298871000

6 0.829266000 0.102461000 -0.355508000

6 0.090890000 -1.053703000 0.007489000

6 0.102287000 1.190708000 -0.889679000

6 -1.287430000 -1.108992000 -0.192362000

6 -1.260891000 1.140988000 -1.087900000

6 -1.973672000 -0.020118000 -0.752045000

1 -1.835112000 -1.999422000 0.073200000

1 -1.758429000 2.002595000 -1.516434000

6 4.309655000 -0.337741000 0.140544000

6 4.966606000 0.167071000 -0.997866000

6 5.082919000 -0.741585000 1.243444000

6 6.351846000 0.280756000 -1.020241000

6 6.468072000 -0.603758000 1.217234000

6 7.097672000 -0.098602000 0.088875000

1 6.825617000 0.641052000 -1.925503000

1 7.025429000 -0.911316000 2.092771000

1 8.177152000 -0.008688000 0.070650000

8 4.555750000 -1.302841000 2.364693000

6 3.351126000 -0.778356000 2.915361000

1 3.293035000 0.301880000 2.752460000

1 2.477456000 -1.258172000 2.475173000

1 3.400145000 -0.980285000 3.985411000

8 4.257332000 0.433325000 -2.134448000

6 3.989914000 1.811967000 -2.357233000

1 3.372699000 2.209275000 -1.549826000

1 4.924317000 2.377112000 -2.439464000

1 3.438849000 1.871996000 -3.294071000

8 0.745900000 2.335913000 -1.265168000

6 1.197699000 3.142907000 -0.183956000

1 0.357005000 3.412331000 0.463557000

1 1.964900000 2.624188000 0.395065000

1 1.617167000 4.044200000 -0.628675000

8 0.775120000 -2.079763000 0.543281000

6 0.087165000 -3.277638000 0.842273000

1 -0.676895000 -3.115992000 1.608812000

1 -0.373428000 -3.702225000 -0.054302000

1 0.843236000 -3.961470000 1.220784000

7 -3.323008000 -0.078838000 -0.982615000

1 -3.768173000 0.796258000 -1.209881000

6 -4.177962000 -1.112958000 -0.430549000

1 -3.960590000 -2.058941000 -0.937888000

1 -3.956771000 -1.263831000 0.637909000

6 -5.664229000 -0.760590000 -0.580193000

1 -5.781691000 -0.186336000 -1.510265000

6 -6.172702000 0.088262000 0.593345000

6 -6.504970000 -2.038651000 -0.708668000

1 -6.210250000 -2.733689000 0.087205000

1 -7.553743000 -1.789556000 -0.520834000

1 -7.220694000 0.345954000 0.402557000

1 -6.170930000 -0.538006000 1.495416000

6 -6.390041000 -2.718009000 -2.071774000

1 -5.357712000 -2.980957000 -2.313143000

1 -6.979783000 -3.635859000 -2.104247000

1 -6.755385000 -2.057312000 -2.861808000

6 -5.395295000 1.371967000 0.881152000

1 -4.353938000 1.138003000 1.130882000

1 -5.368736000 1.998316000 -0.021372000

6 -6.008032000 2.181999000 2.022490000

1 -6.046470000 1.557926000 2.921796000

1 -7.045544000 2.428926000 1.773390000

6 -5.228087000 3.461131000 2.315045000

1 -5.202684000 4.111056000 1.436421000

1 -5.675788000 4.025215000 3.135272000

1 -4.194914000 3.232517000 2.588856000

**Triplet (T_1_)**

7 3.094429000 0.962217000 0.431106000

7 2.174185000 1.266055000 -0.420515000

6 0.880350000 0.857609000 -0.393074000

6 0.402326000 -0.249718000 0.361004000

6 -0.035282000 1.483574000 -1.278966000

6 -0.904618000 -0.698502000 0.230578000

6 -1.332440000 1.039695000 -1.406798000

6 -1.786082000 -0.060517000 -0.657861000

1 -1.237723000 -1.555116000 0.797685000

1 -1.987639000 1.546985000 -2.105310000

6 3.996394000 -0.054216000 0.240655000

6 3.809946000 -1.149851000 -0.644982000

6 5.141427000 -0.072302000 1.078910000

6 4.705196000 -2.206089000 -0.669516000

6 6.026364000 -1.142909000 1.043646000

6 5.810338000 -2.207730000 0.178404000

1 4.505766000 -3.029295000 -1.345166000

1 6.880299000 -1.113972000 1.708448000

1 6.505897000 -3.037692000 0.159760000

8 5.400585000 0.902443000 1.986280000

6 5.304025000 2.268438000 1.586726000

1 5.498054000 2.372295000 0.516519000

1 4.318077000 2.669533000 1.813637000

1 6.069933000 2.800399000 2.151120000

8 2.686113000 -1.218997000 -1.421318000

6 2.803313000 -0.584790000 -2.692556000

1 3.086850000 0.464453000 -2.578621000

1 3.539670000 -1.108725000 -3.309945000

1 1.819152000 -0.641264000 -3.155601000

8 0.376431000 2.511692000 -2.075726000

6 0.682436000 3.722152000 -1.388725000

1 -0.197896000 4.074323000 -0.841880000

1 1.518962000 3.583534000 -0.702757000

1 0.949564000 4.447064000 -2.155482000

8 1.308506000 -0.830912000 1.168385000

6 1.055815000 -2.139950000 1.637676000

1 0.244261000 -2.151106000 2.371519000

1 0.815282000 -2.804132000 0.802514000

1 1.978247000 -2.466117000 2.113851000

7 -3.077563000 -0.506334000 -0.821783000

1 -3.689046000 0.130766000 -1.308329000

6 -3.723827000 -1.384581000 0.137600000

1 -3.334891000 -2.399964000 0.007253000

1 -3.476638000 -1.076650000 1.165837000

6 -5.249963000 -1.374311000 -0.024407000

1 -5.474329000 -1.220568000 -1.089666000

6 -5.901325000 -0.245091000 0.786764000

6 -5.842182000 -2.729012000 0.388336000

1 -5.422891000 -3.013102000 1.361352000

1 -6.918617000 -2.609399000 0.544714000

1 -6.979057000 -0.254894000 0.587591000

1 -5.784860000 -0.478973000 1.853370000

6 -5.606912000 -3.840737000 -0.632279000

1 -4.544147000 -3.995026000 -0.831869000

1 -6.017615000 -4.789506000 -0.282131000

1 -6.088999000 -3.597448000 -1.582363000

6 -5.365944000 1.162867000 0.529038000

1 -4.298278000 1.214560000 0.772123000

1 -5.456934000 1.403603000 -0.539318000

6 -6.108522000 2.226723000 1.335919000

1 -6.029721000 1.985432000 2.401399000

1 -7.174788000 2.188801000 1.088474000

6 -5.568594000 3.632118000 1.084044000

1 -5.663166000 3.901901000 0.028883000

1 -6.106564000 4.379701000 1.669879000

1 -4.510091000 3.694984000 1.349076000

***Cis*-AZO-N**

**Singlet (S_0_)**

7 3.569992000 0.535340000 0.984521000

7 2.528854000 0.853103000 1.575700000

6 1.245863000 0.603530000 0.997876000

6 0.306135000 -0.094697000 1.759650000

6 0.853768000 1.093905000 -0.258551000

6 -0.963582000 -0.363759000 1.271375000

6 -0.419122000 0.854113000 -0.741545000

6 -1.341781000 0.104048000 0.005267000

1 -1.625420000 -0.958399000 1.885948000

1 -0.683725000 1.257062000 -1.712404000

6 3.536469000 -0.236076000 -0.223101000

6 2.999414000 -1.529047000 -0.265570000

6 4.189176000 0.275709000 -1.348662000

6 3.090087000 -2.277845000 -1.434631000

6 4.265436000 -0.469358000 -2.515965000

6 3.711906000 -1.745165000 -2.556645000

1 2.655256000 -3.269699000 -1.440625000

1 4.745408000 -0.028314000 -3.380895000

1 3.767548000 -2.327692000 -3.468180000

8 4.690631000 1.550135000 -1.317100000

6 5.967508000 1.639369000 -0.688360000

1 5.910548000 1.300616000 0.348562000

1 6.702787000 1.043266000 -1.237483000

1 6.253520000 2.689404000 -0.718288000

8 2.350983000 -2.052139000 0.816939000

6 3.196156000 -2.454067000 1.891061000

1 3.809786000 -3.308257000 1.588344000

1 3.842070000 -1.633043000 2.213970000

1 2.533354000 -2.731928000 2.707585000

8 1.724343000 1.804628000 -1.035648000

6 2.014915000 3.117706000 -0.565717000

1 1.108286000 3.730623000 -0.580485000

1 2.427374000 3.094250000 0.446599000

1 2.759952000 3.522154000 -1.246789000

8 0.661611000 -0.612606000 2.976577000

6 0.711214000 0.361134000 4.017635000

1 1.451713000 1.131074000 3.791909000

1 -0.274621000 0.815925000 4.154872000

1 0.996030000 -0.171881000 4.923190000

7 -2.604309000 -0.121731000 -0.501939000

1 -2.691786000 -0.008851000 -1.500673000

6 -3.506089000 -1.087479000 0.097386000

1 -3.008137000 -2.061846000 0.196460000

1 -3.758614000 -0.756134000 1.113300000

6 -4.808063000 -1.229543000 -0.702233000

1 -4.567208000 -1.120864000 -1.770338000

6 -5.841319000 -0.159521000 -0.327139000

6 -5.400935000 -2.631642000 -0.499300000

1 -5.412791000 -2.855429000 0.574393000

1 -6.447321000 -2.620400000 -0.819101000

1 -6.727146000 -0.303766000 -0.956919000

1 -6.167846000 -0.344976000 0.705116000

6 -4.660241000 -3.731527000 -1.257669000

1 -3.605675000 -3.783143000 -0.977871000

1 -5.100730000 -4.710613000 -1.060438000

1 -4.709397000 -3.554264000 -2.335126000

6 -5.377853000 1.290258000 -0.452490000

1 -4.532130000 1.475508000 0.217031000

1 -5.002770000 1.468836000 -1.468553000

6 -6.496443000 2.284978000 -0.147905000

1 -6.875997000 2.099273000 0.862943000

1 -7.335631000 2.106983000 -0.829310000

6 -6.036098000 3.735752000 -0.265915000

1 -5.677504000 3.948351000 -1.276479000

1 -6.845317000 4.434094000 -0.043680000

1 -5.215295000 3.940297000 0.426189000

**Triplet (T_1_)**

7 -3.189550000 -0.633682000 0.662644000

7 -2.197208000 -0.009805000 1.205708000

6 -0.918220000 -0.040318000 0.757286000

6 0.071508000 0.613720000 1.535866000

6 -0.496970000 -0.591936000 -0.488887000

6 1.381013000 0.723978000 1.108261000

6 0.808822000 -0.486605000 -0.907380000

6 1.773763000 0.178475000 -0.125305000

1 2.074950000 1.255972000 1.744702000

1 1.073556000 -0.909321000 -1.870412000

6 -4.072267000 -0.023930000 -0.188034000

6 -3.930270000 1.280026000 -0.730814000

6 -5.185593000 -0.797807000 -0.608442000

6 -4.860776000 1.770404000 -1.634259000

6 -6.102111000 -0.294415000 -1.514739000

6 -5.941956000 0.989688000 -2.030145000

1 -4.703021000 2.763992000 -2.036574000

1 -6.926479000 -0.929087000 -1.815452000

1 -6.656363000 1.378393000 -2.745202000

8 -5.332946000 -2.081644000 -0.166128000

6 -5.755335000 -2.198403000 1.189005000

1 -5.020208000 -1.758318000 1.864940000

1 -6.727294000 -1.713070000 1.322825000

1 -5.849497000 -3.264112000 1.390010000

8 -2.842540000 2.042065000 -0.417206000

6 -2.998036000 2.836881000 0.755769000

1 -3.771964000 3.594735000 0.597556000

1 -3.251833000 2.213846000 1.616671000

1 -2.036317000 3.314413000 0.934385000

8 -1.408110000 -1.185286000 -1.311618000

6 -1.660040000 -2.557765000 -1.015323000

1 -0.768767000 -3.156122000 -1.229633000

1 -1.956648000 -2.683116000 0.028348000

1 -2.483382000 -2.865273000 -1.657291000

8 -0.269804000 1.221267000 2.709690000

6 -0.611211000 0.332122000 3.769865000

1 -1.496518000 -0.254003000 3.519694000

1 0.230981000 -0.332524000 3.986500000

1 -0.814403000 0.957227000 4.637540000

7 3.068979000 0.259280000 -0.568344000

1 3.227509000 0.026635000 -1.536359000

6 4.071180000 1.086985000 0.073398000

1 3.716790000 2.124514000 0.134397000

1 4.216720000 0.739708000 1.105261000

6 5.419862000 1.023147000 -0.655438000

1 5.221271000 0.940903000 -1.734468000

6 6.258636000 -0.185888000 -0.221362000

6 6.205475000 2.322384000 -0.424673000

1 6.195312000 2.552738000 0.647649000

1 7.253276000 2.149948000 -0.688430000

1 7.186062000 -0.185871000 -0.805907000

1 6.557641000 -0.038099000 0.825103000

6 5.680258000 3.513429000 -1.224009000

1 4.633147000 3.728483000 -0.999679000

1 6.254428000 4.415988000 -1.006961000

1 5.756453000 3.319154000 -2.296872000

6 5.587798000 -1.550937000 -0.361045000

1 4.693123000 -1.598484000 0.267942000

1 5.240734000 -1.685940000 -1.393924000

6 6.526488000 -2.699206000 0.004954000

1 6.882992000 -2.557168000 1.031080000

1 7.413126000 -2.661120000 -0.637253000

6 5.856087000 -4.064770000 -0.122401000

1 5.515193000 -4.236323000 -1.146787000

1 6.538792000 -4.874151000 0.143227000

1 4.983665000 -4.131857000 0.532523000

**Z(cis)-Azobenzene**

**Singlet (S_0_)**

6 -3.655010000 1.298691000 -0.000038000

6 -2.282554000 1.113369000 0.000002000

6 -1.765942000 -0.185220000 0.000030000

6 -2.622878000 -1.281617000 0.000022000

6 -3.999590000 -1.089506000 -0.000015000

6 -4.516234000 0.200488000 -0.000046000

1 -4.062158000 2.302931000 -0.000063000

1 -1.596580000 1.949956000 0.000013000

1 -2.187234000 -2.273597000 0.000050000

1 -4.665129000 -1.944186000 -0.000021000

1 -5.588694000 0.355535000 -0.000072000

7 -0.374904000 -0.493918000 0.000075000

7 0.374904000 0.493918000 0.000046000

6 1.765942000 0.185221000 0.000014000

6 2.622878000 1.281617000 0.000019000

6 2.282554000 -1.113369000 -0.000016000

6 3.999590000 1.089506000 -0.000009000

1 2.187234000 2.273597000 0.000043000

6 3.655010000 -1.298691000 -0.000036000

1 1.596580000 -1.949956000 -0.000018000

6 4.516235000 -0.200488000 -0.000036000

1 4.665129000 1.944186000 -0.000015000

1 4.062158000 -2.302931000 -0.000050000

1 5.588694000 -0.355535000 -0.000058000

**Triplet (T_1_)**

6 -3.655010000 -1.298691000 0.000038000

6 -2.282554000 -1.113369000 -0.000002000

6 -1.765942000 0.185220000 -0.000030000

6 -2.622878000 1.281617000 -0.000022000

6 -3.999590000 1.089506000 0.000015000

6 -4.516234000 -0.200488000 0.000046000

1 -4.062158000 -2.302931000 0.000063000

1 -1.596580000 -1.949956000 -0.000013000

1 -2.187234000 2.273597000 -0.000050000

1 -4.665129000 1.944186000 0.000021000

1 -5.588694000 -0.355535000 0.000072000

7 -0.374904000 0.493918000 -0.000075000

7 0.374904000 -0.493918000 -0.000046000

6 1.765942000 -0.185221000 -0.000014000

6 2.622878000 -1.281617000 -0.000019000

6 2.282554000 1.113369000 0.000016000

6 3.999590000 -1.089506000 0.000009000

1 2.187234000 -2.273597000 -0.000043000

6 3.655010000 1.298691000 0.000036000

1 1.596580000 1.949956000 0.000018000

6 4.516235000 0.200488000 0.000036000

1 4.665129000 -1.944186000 0.000015000

1 4.062158000 2.302931000 0.000050000

1 5.588694000 0.355535000 0.000058000

**3. Supporting Figures**


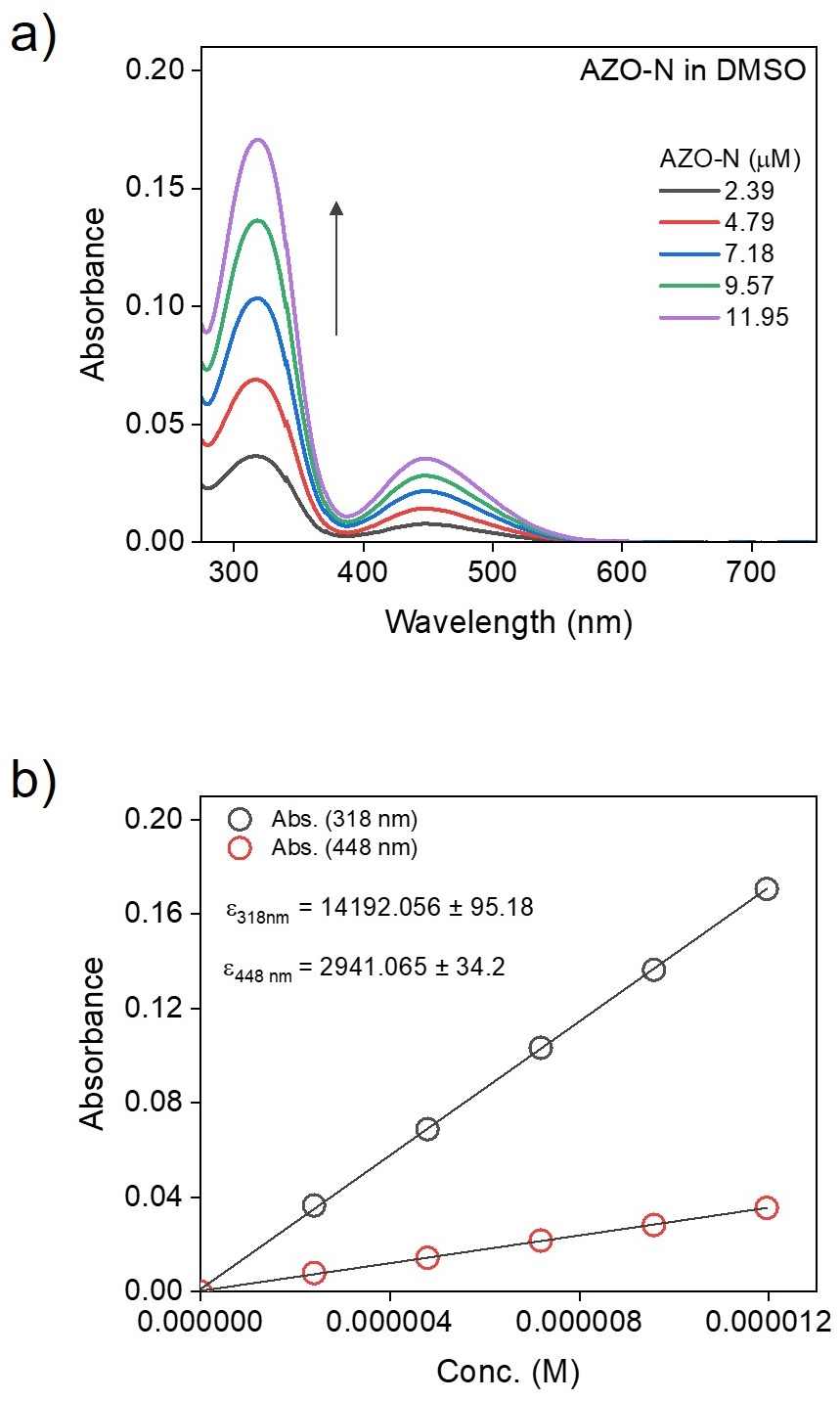


**Figure S9.** a) Concentration dependent absorption spectra of **AZO-N** in DMSO, and b) corresponding **AZO-N** concentration vs absorbance profiles at 318 and 448 nm for calculating the extinction coefficients. Spectra recorded in 1 cm pathlength quartz cuvette


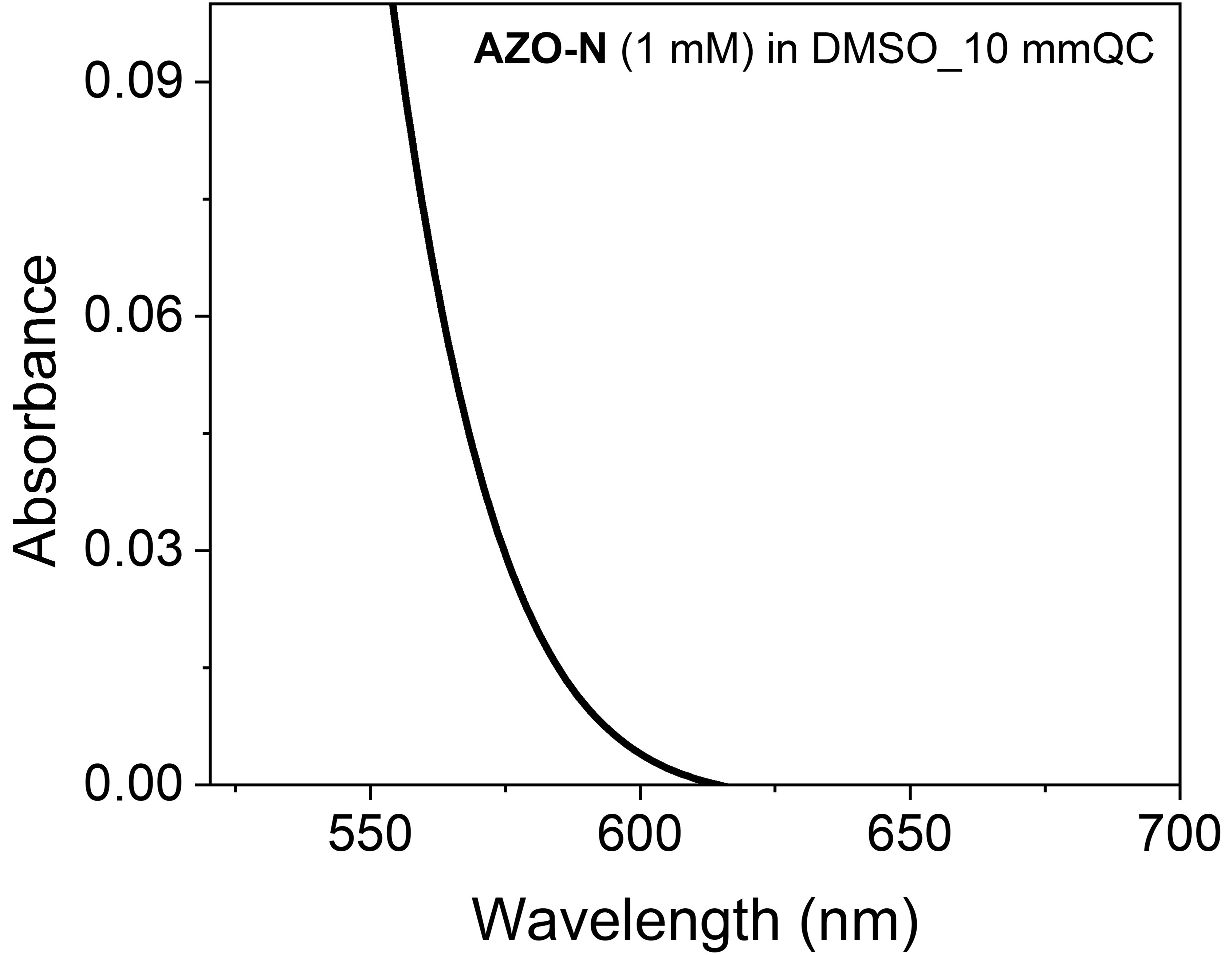


**Figure S10.** Absorption spectrum of **AZO-N** (1 mM) in DMSO in 1 cm quartz cuvette.


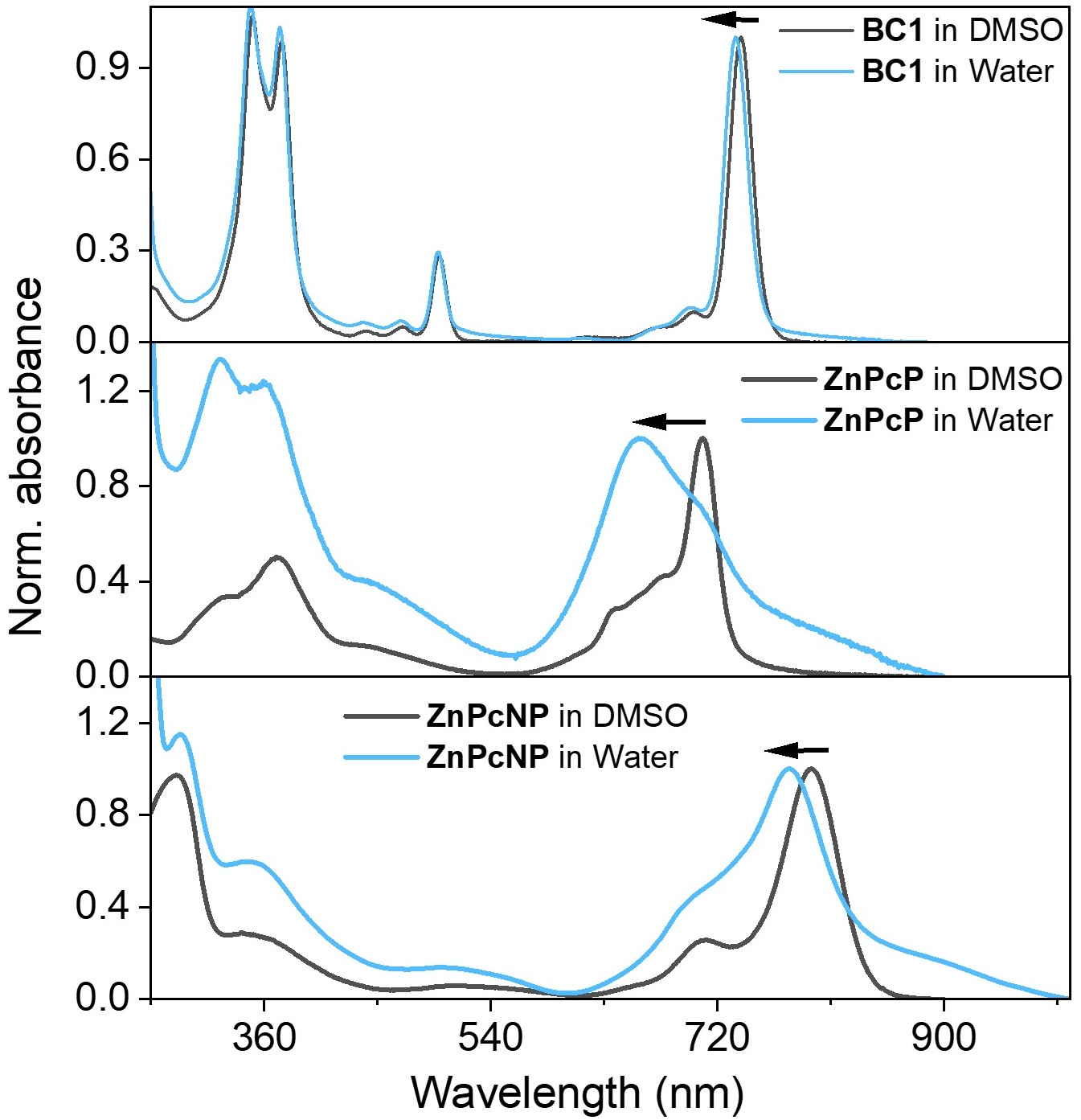


**Figure S11.** Comparative normalized absorption profiles of **BC1**, **ZnPcP**, and **ZnPcNP** in DMSO and PBS solution (10 mM, pH. 7.4).


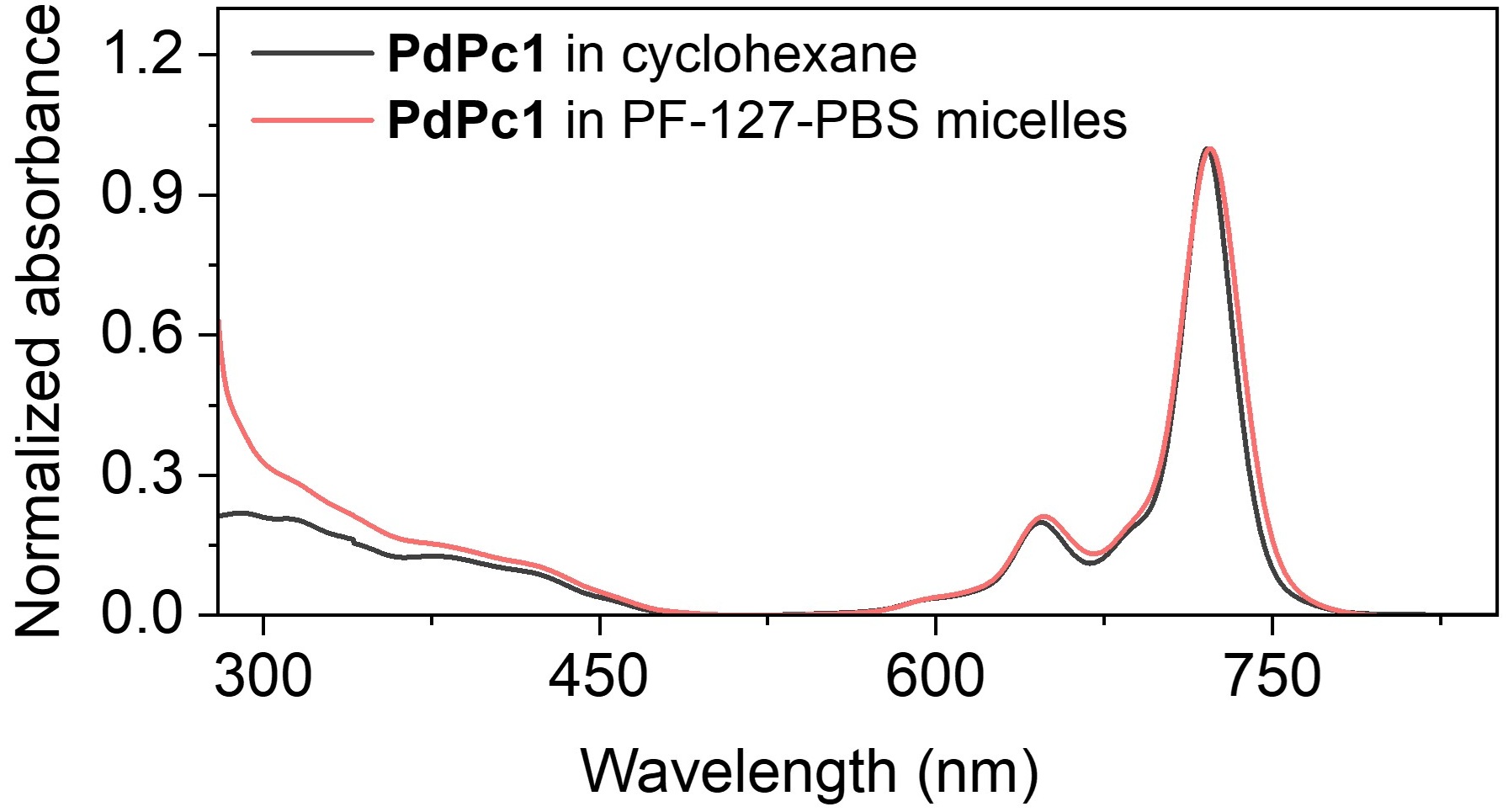


**Figure S12.** Comparative absorption spectra of **PdPc1** in cyclohexane and PF-127 (7%) in PBS (pH. 7.4). Conc. = 1.33 µM.


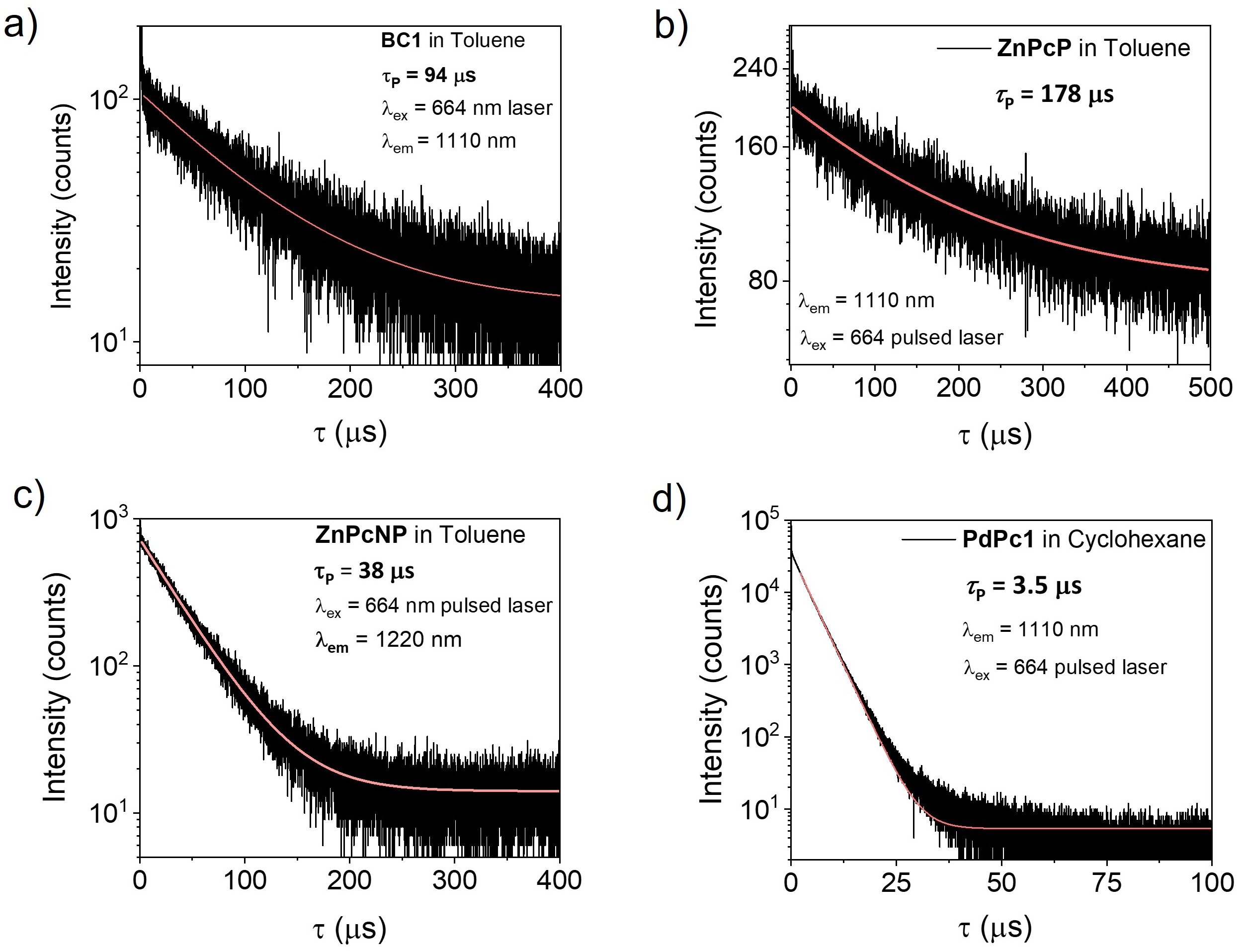


**Figure S13.** Time-resolved phosphorescence decay profiles of **BC1**, **ZnPcP**, **ZnPcNP** and **PdPc1** and in the argon-saturated toluene or cyclohexane. λ_ex_ = 664 nm pulsed laser. λ_em_ = 1110 nm or 1220 nm. The red line indicates single exponential fitting of decay profiles.


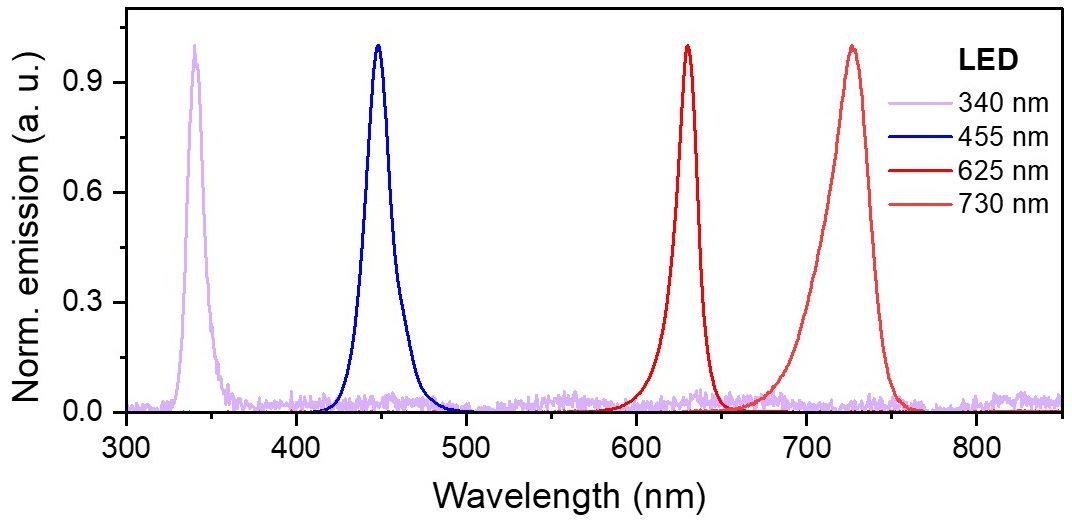


**Figure S14.** Normalized emission spectra of LEDs used in this work.


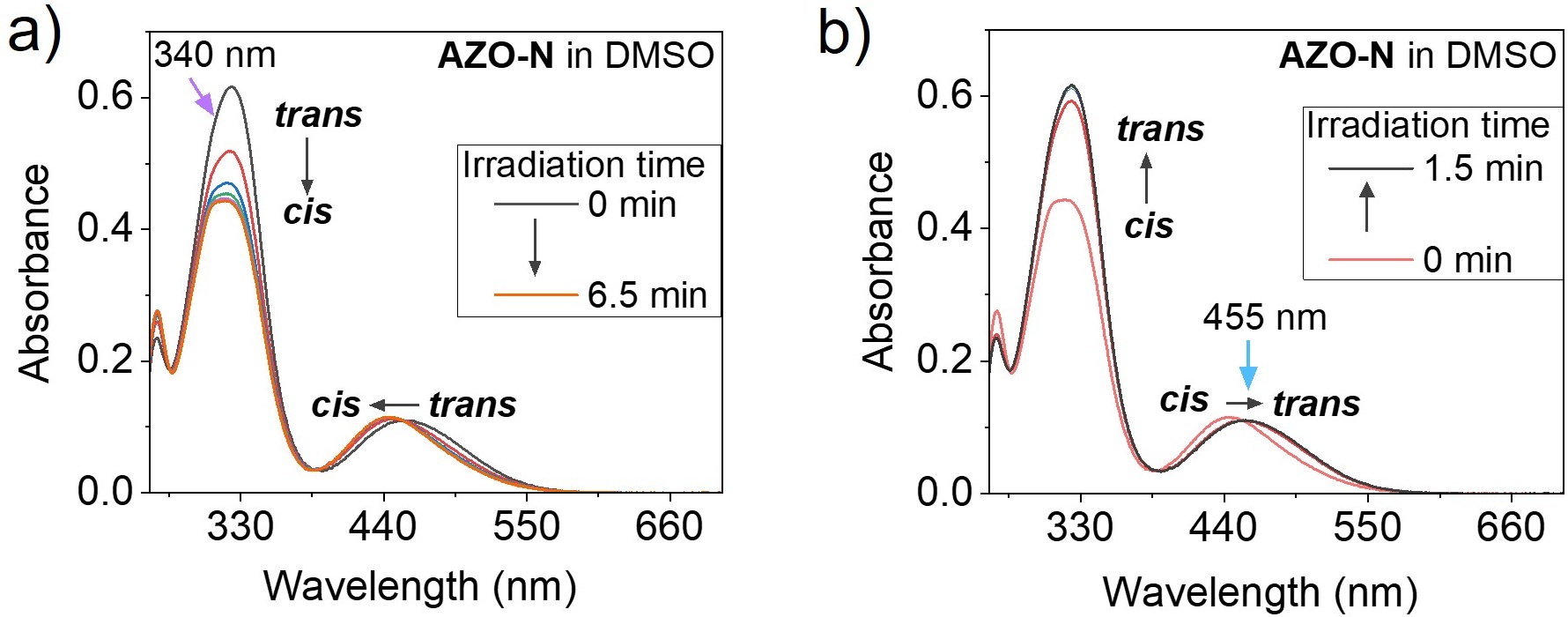


**Figure S15.** a, b) Absorption profiles showing of *trans*$\boldsymbol{\leftrightarrow}$*cis* photoswitching of **AZO-N** in DMSO upon 340 (0.76 mW cm^-2^) and 455 nm (3.05 mW cm^-2^) excitation, respectively. **AZO-N** = 36 µM; 1 cm pathlength quartz cuvette.


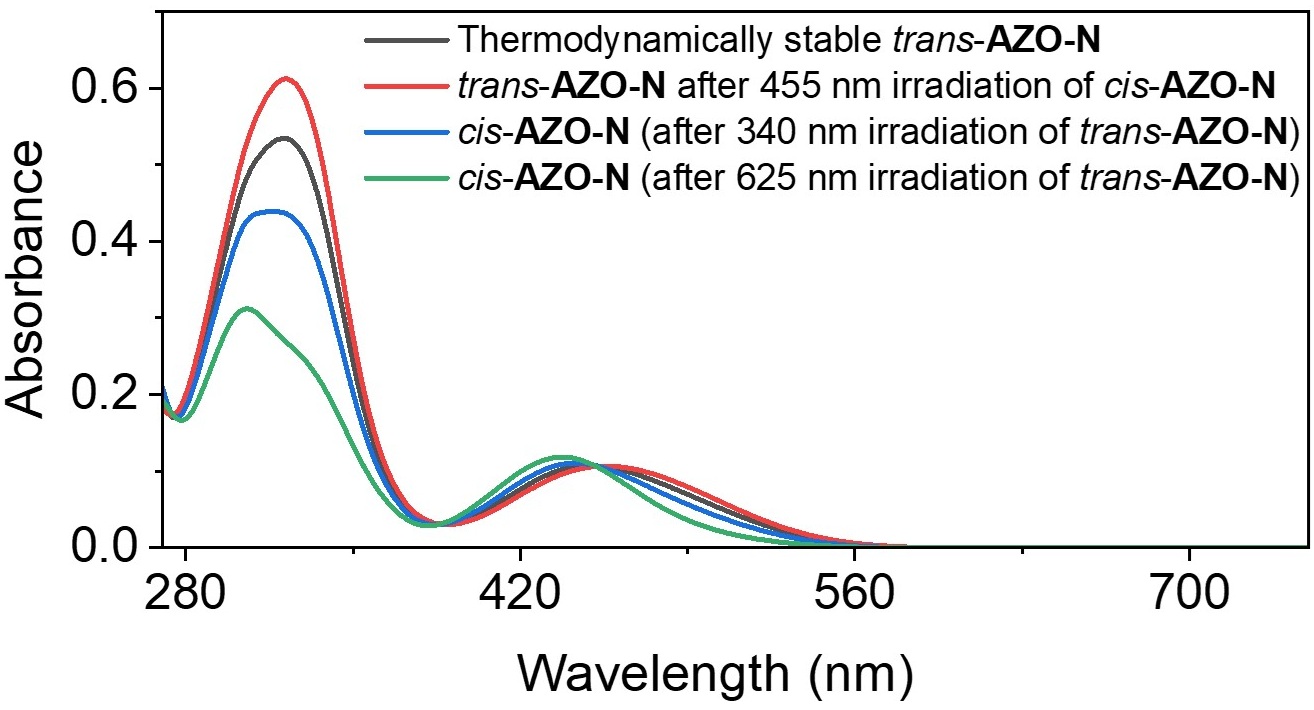


**Figure S16.** Comparative absorption spectra of different isomers of **AZO-N** in DMSO. **AZO-N** = 36 µM; 1 cm pathlength quartz cuvette.

**^1^H NMR experiments AZO-N to calculate % trans and % cis isomers upon photoisomerization**

**Irradiation of AZO-N (1mM) in DMSO-*d*_6_**

First, the NMR sample was irradiated with 455 nm LED, for 1, 2, 7, and 20 mins, followed by the recording of ^1^H NMR spectra. The relevant peaks of the **AZO-N** between 8.5-6 ppm, 4.5-3.5 ppm, and 2-0.5 ppm undergo a decrease/increase in intensity. It is to be noted that the **AZO-N** was already in a PSS state at the beginning of the irradiation after dissolving in the deuterated solvent. When irradiated after that in a second irradiation experiment with a 625 nm LED for 10, 20, 30, 40, and 51 mins. ^1^H NMR spectra were recorded. PSS was determined for both irradiation experiments. The peaks in the region of 8.0-5.5 ppm were used to calculate the PSS percentages. All other peaks seem to overlap with solvent residual peaks or were not unambiguously resolved. Irradiation with 455 nm LED from thermodynamically stable PSS

**
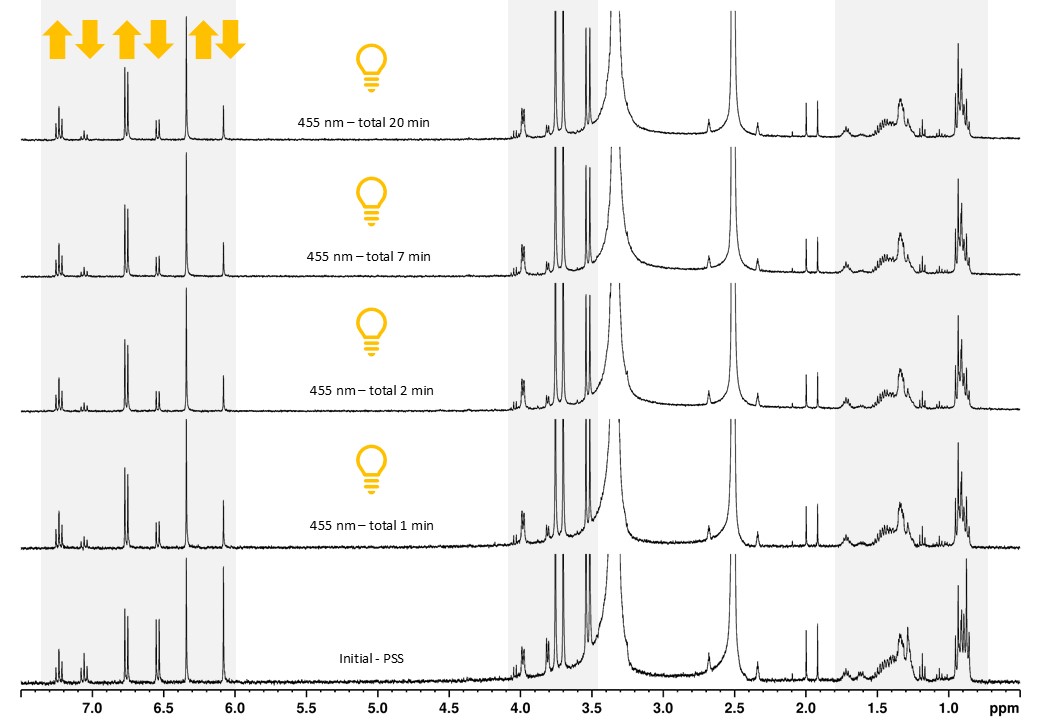
**

**Figure S17.** ^1^H NMR spectra of 455 nm LED irradiation of **AZO-N** (1mM) solution in DMSO-d_6_ at room temperature.


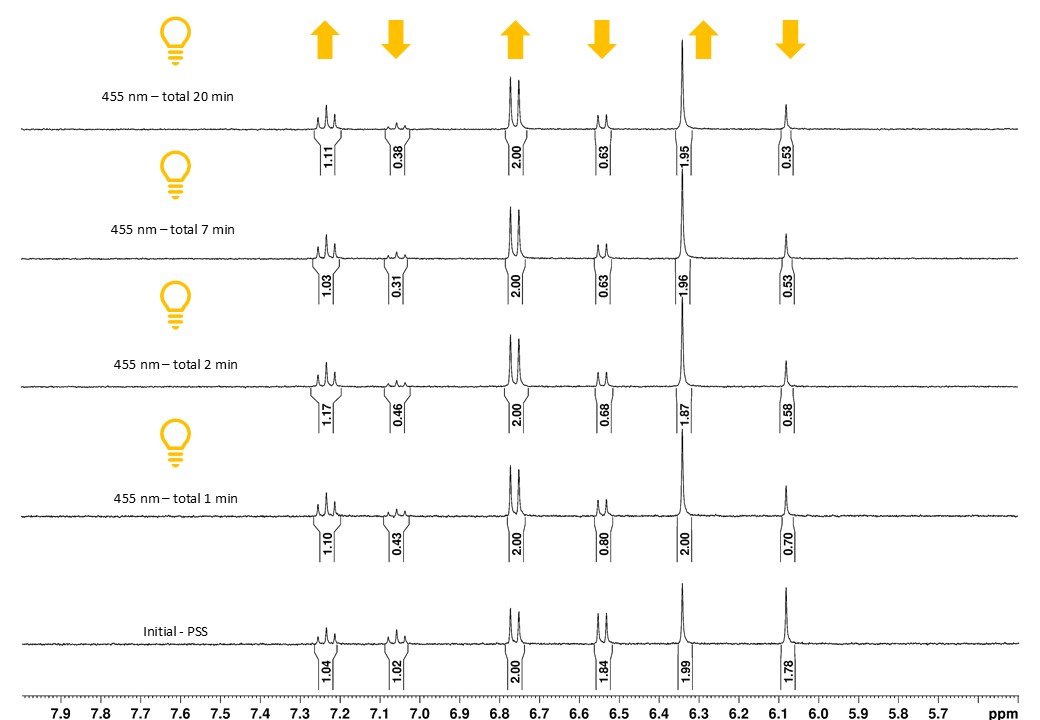


**Figure S18.** ^1^H NMR spectra (range 8.0-5.5ppm) of 455 nm LED irradiation of **AZO-N** (1mM) solution in DMSO-d_6_ at room temperature.

**Table S1. Summary of the change in *%* trans-AZO-N upon irradiation of PSS with 455 nm LED.** The *trans*-**AZO-N** can be reached in around 79% after irradiation with 455 nm after a total irradiation time of 7 min. Even after a total of 20 min of irradiation time, the amount of *trans*-**AZO-N** in the PSS did not change further.

| **Time of irradiation (min)** | **% of *trans*-isomer in PSS (7.3-7.0ppm)** | **% of *trans*-isomer in PSS (6.8-6.5ppm)** | **% of *trans*-isomer in PSS (6.4-6.0ppm)** |
| --- | --- | --- | --- |
| Initial PSS | 51 | 52 | 53 |
| 1 | 72 | 71 | 74 |
| 2 | 72 | 75 | 76 |
| 7 | 77 | 76 | 79 |
| 20 | 75 | 76 | 79 |


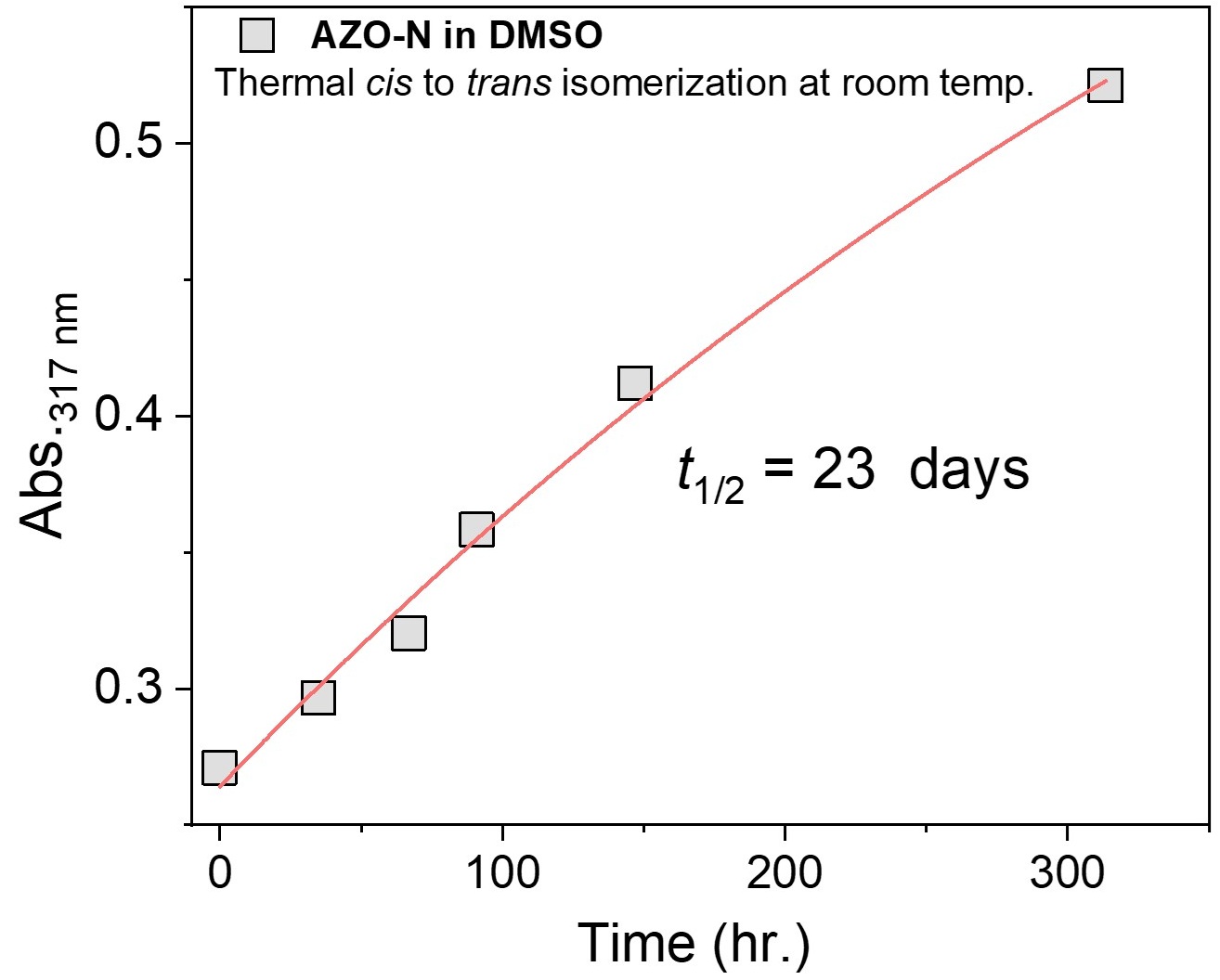


**Figure S19.** Plot showing thermal kinetics of *cis*-to-*trans* photoswitching of **AZO-N** in DMSO at room temperature.


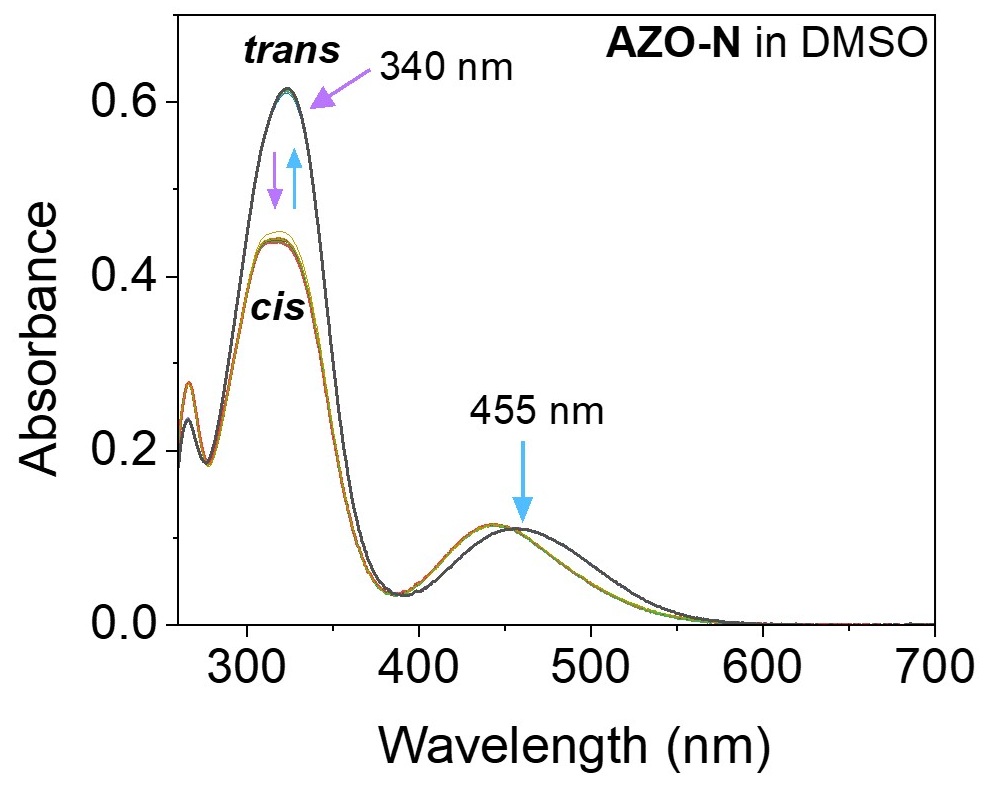


**Figure S20.** Absorption profiles showing ***trans***$\boldsymbol{\leftrightarrow}$***cis*** photoswitching of **AZO-N** in DMSO upon periodic excitation of with 340 (2.5 min, 0.76 mW cm^-2^) and 455 nm (1.5 min, 3.05 mW cm^-2^) light for 12 successive cycles. **AZO-N** = 36 µM; 1 cm pathlength quartz cuvette.


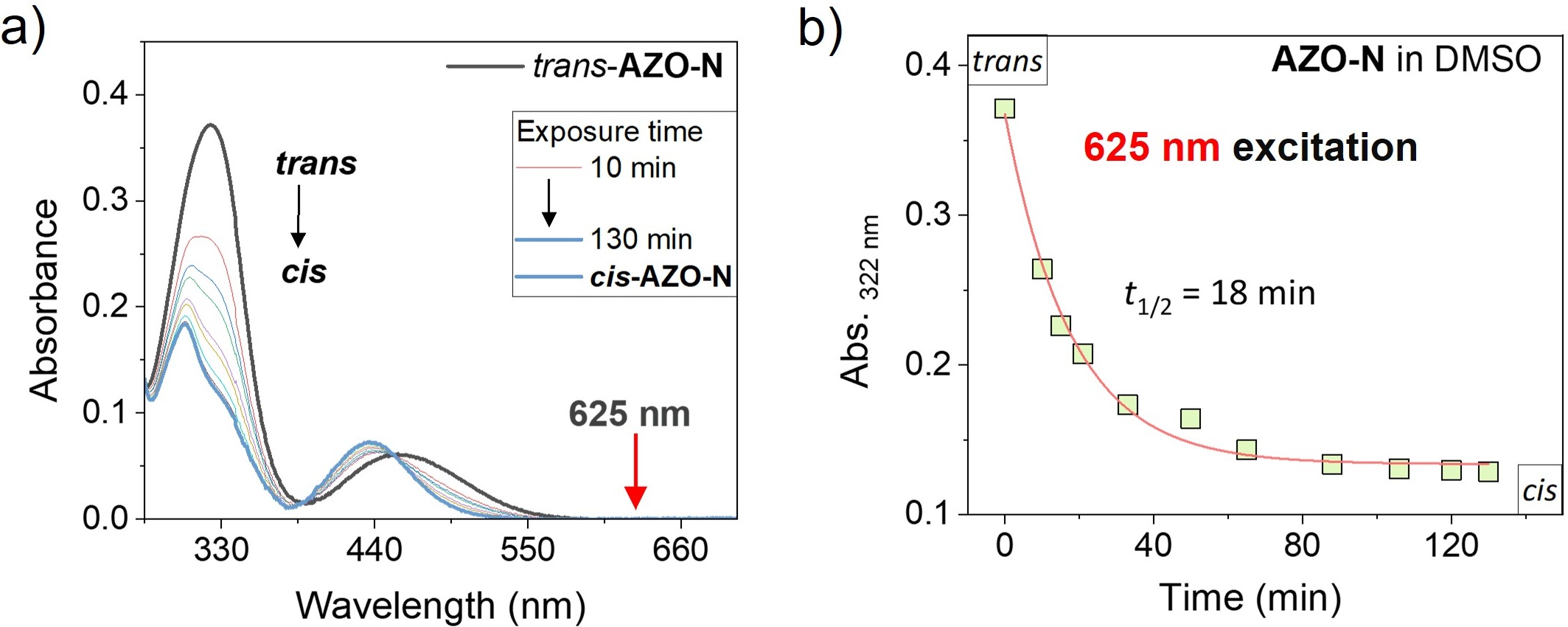


**Figure S21.** a) Absorption profiles showing *trans-*to*-cis* photoswitching of **AZO-N** in DMSO upon 625 nm excitation (154 mW cm^-2^). b) Corresponding photo-kinetics profile of *trans*-to-cis isomerization. **AZO-N** = 187 µM; 0.1 cm pathlength quartz cuvette.

**Calculation of the photoisomerization quantum yield of *trans*-to-*cis* isomerization upon 625 nm LED excitation**

**Lamp spectrum of 625nmThorlabs LED (M625L4)**

The lamp spectrum of the 625 Thorlabs LED (M625L4) shows a maximum at 636 nm and an extended tail up to 560 nm.


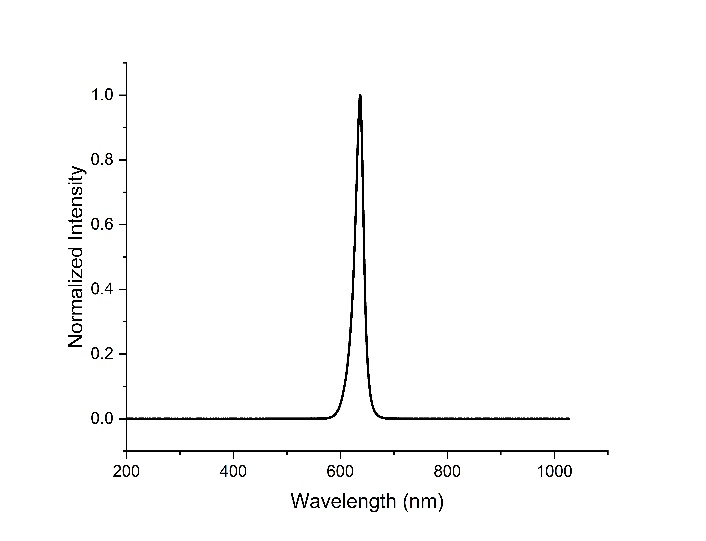

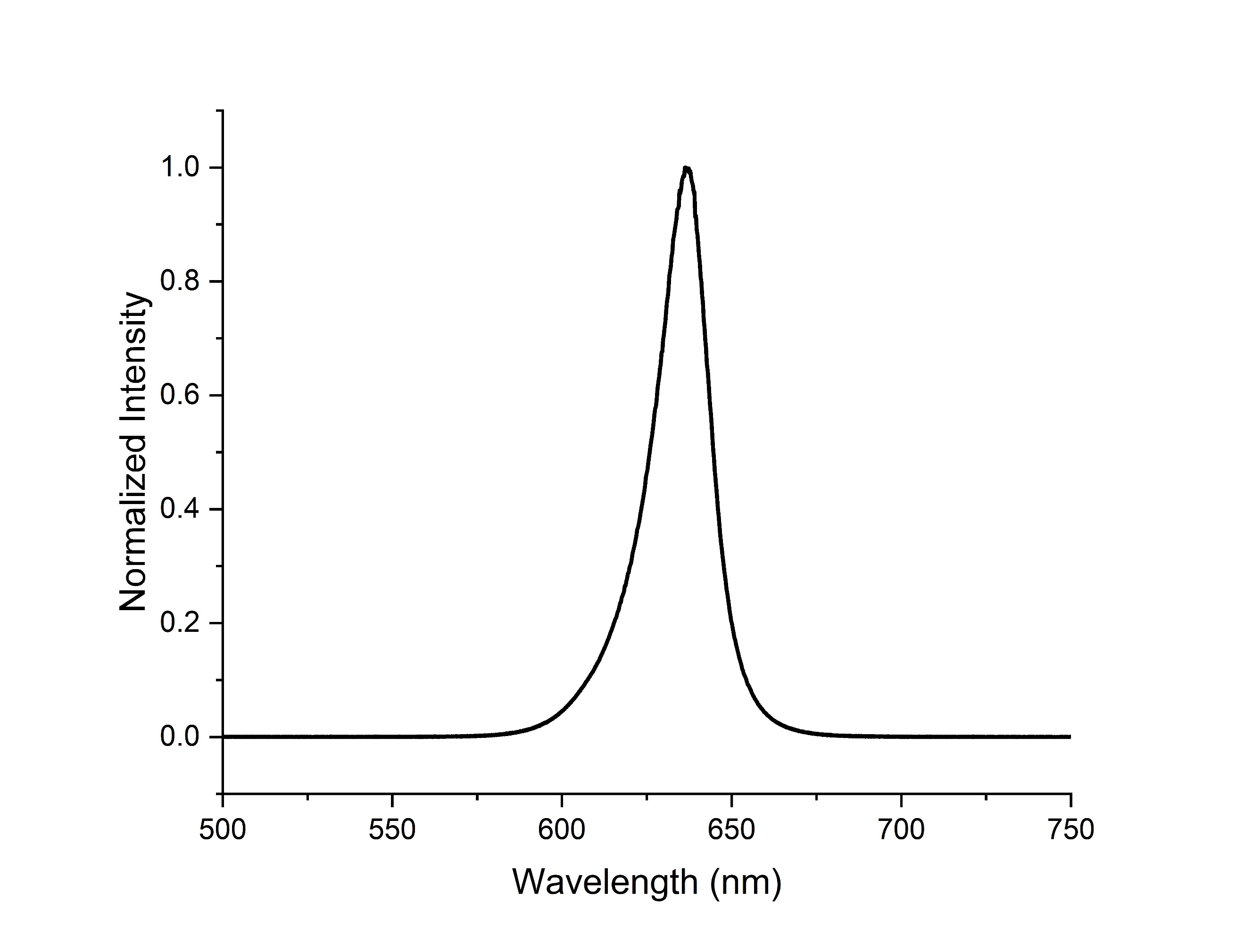


**Figure S22**. Left: Lamp spectrum of 625 nm Thorlabs LED (M625L4), right: Zoom.

**Irradiation of AZO-N at a lower concentration (18 μM)**

The setup and software used for the irradiation is described here.^[3]^ Irradiation was done with a 625 nm Thorlabs LED (M625L4) equipped with a SM1U collimator in a flow cuvette of 80μL (1 cm pathlength quartz cuvette). The distance from the LED to the cuvette was 5.5 cm. The LED with collimator was attached to the setup as shown below. Irradiation was carried out at 60, 180, 300, 600, 1200, 1800, 2400 and 3000s (50 min). The absorbance decrease was monitored after each time step by taking at least five UV/Vis spectra each time, from which the average was taken.


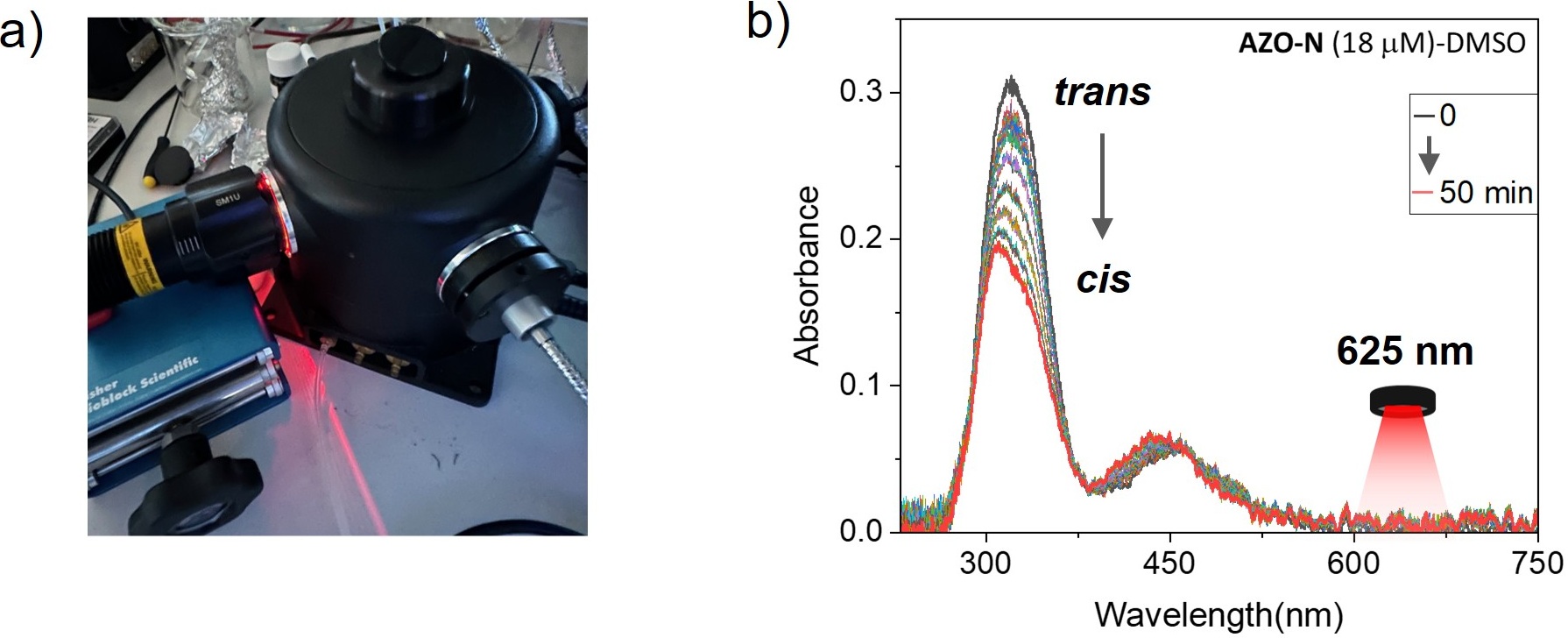


**Figure S23**. Left: Photo of the modified setup. Right: Change in absorption spectra of **AZO-N** in DMSO (concentration = 18 µM) upon irradiation at 625 nm.

**Photoisomerization Quantum Yield of AZO-N in DMSO at 1mM concentration**

Determination of the photon flux of the 625nm Thorlabs LED (M625L4) equipped with a SM1U collimator was carried out using a Power meter (PM100USB) with a S121C - Standard Photodiode Power Sensor (400-1100nm) and the software Thorlabs Optical Power Monitor, measuring the power received after a flow cuvette of 80 μL (1 cm pathlength quartz cuvette). The distance from the LED to the cuvette was 5.5 cm. The measurement was carried out 4$\times$ in complete darkness. The average power over 4 measurements received was 5.78 mW, which converts to a photon flux of 4.6$\times$10^+16^ s^-1^ considering the area of the irradiated window of the 80μL cuvette compared to the area of the sensor.

The setup and analysis software used for the determination of the Quantum yield are described here.^12^ The sample was irradiated at a distance of 5.5 cm. The sample was irradiated for 5, 10, 20, 40, 60, 80, 120, and 230 min. The absorbance decrease was monitored after each time step by taking at least five UV/Vis spectra each time, from which the average was taken. The quantum yield was determined at the analyzing wavelength of 530 nm.


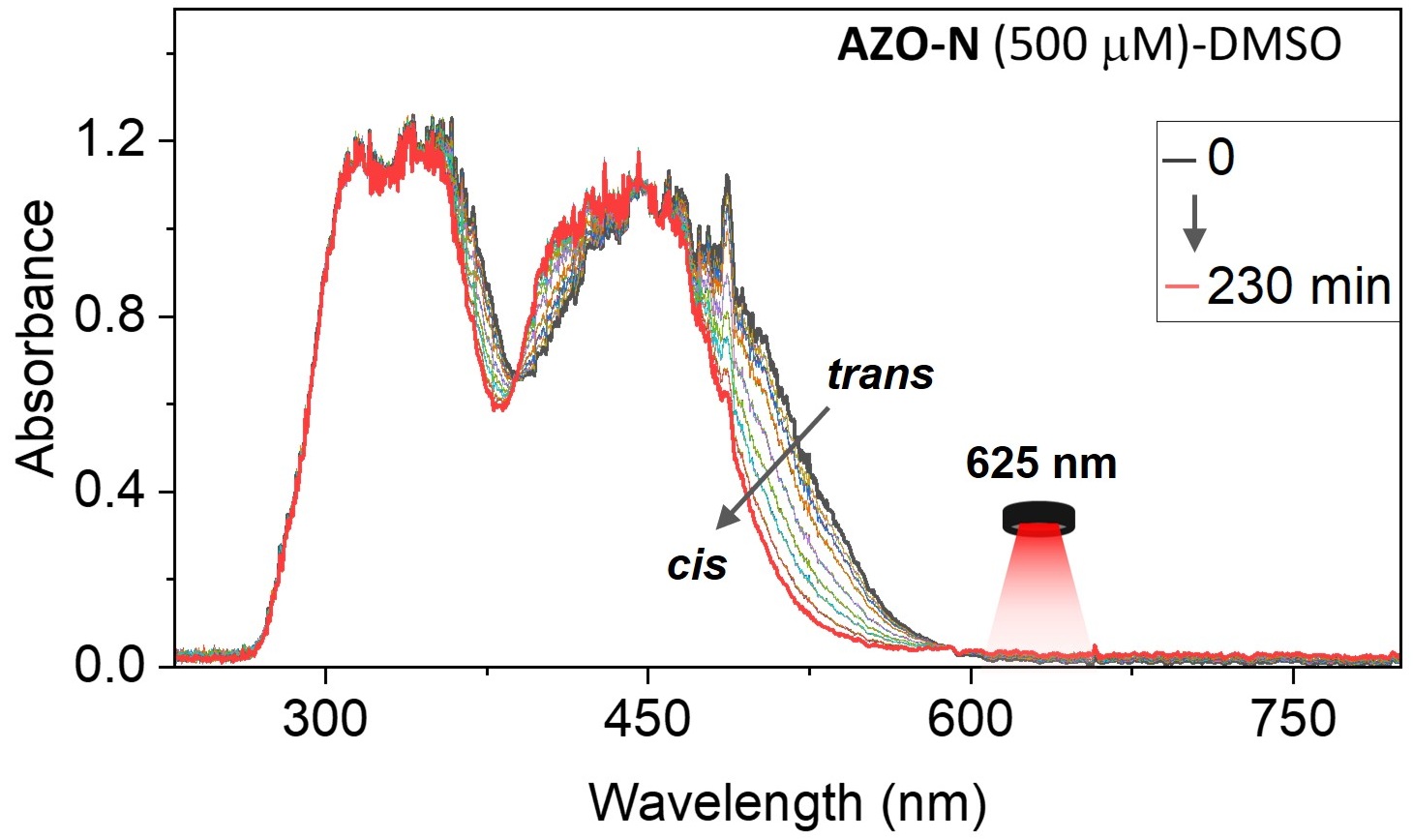


**Figure S24**. Spectral change of AZO-N in DMSO (concentration = 500 µM) upon irradiation at 625 nm. (Roughness of the spectra due to saturation of the detector at this concentration)

**
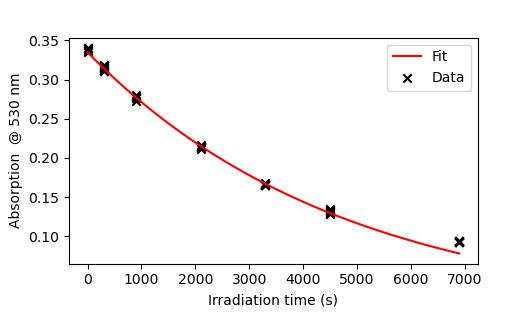
**

**Figure S25**. QY measurement for AZO-N in DMSO (concentration = 500 µM) upon 625 nm LED irradiation.

Average QY = 1.03±0.08 % for the analysis wavelength of 530 nm. When calculated with the extinction coefficient (585 M^-1^ cm^-1^) at 530 nm, then an Average QY = 1.3±0.1 % was obtained.

**Irradiation with 625 nm LED from the PSS with the highest amount of *trans*-AZO-N**


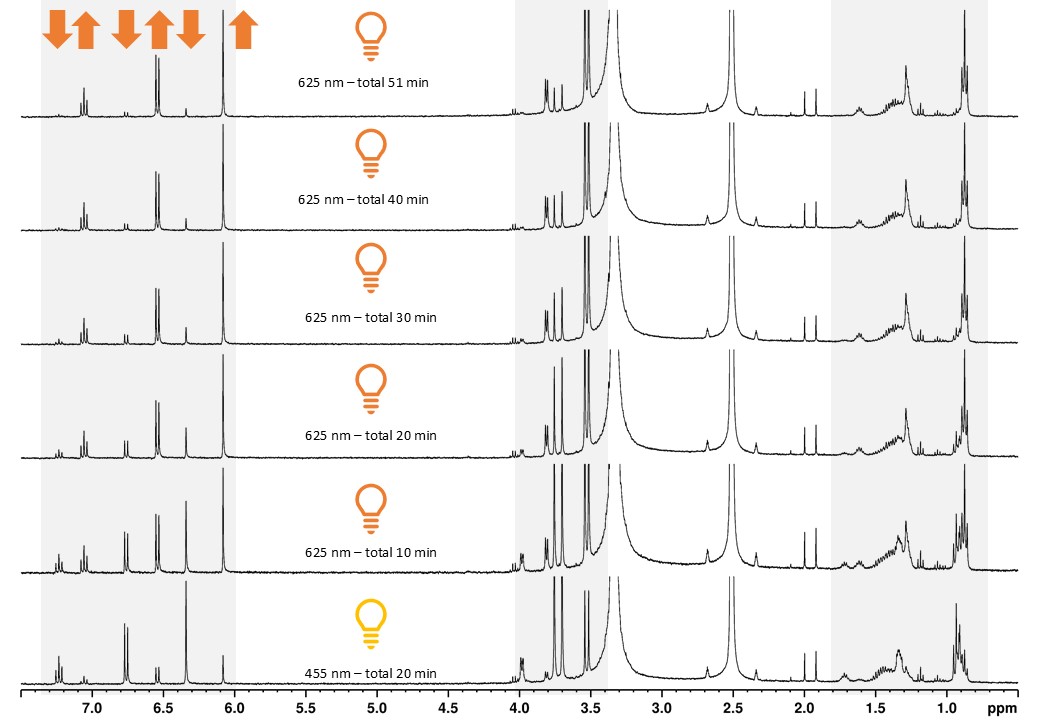


**Figure S26.** ^1^H NMR spectra (range 8.0-5.5ppm) of *trans*-**AZO-N** upon 625 nm LED irradiation in DMSO-d_6_ at room temperature.


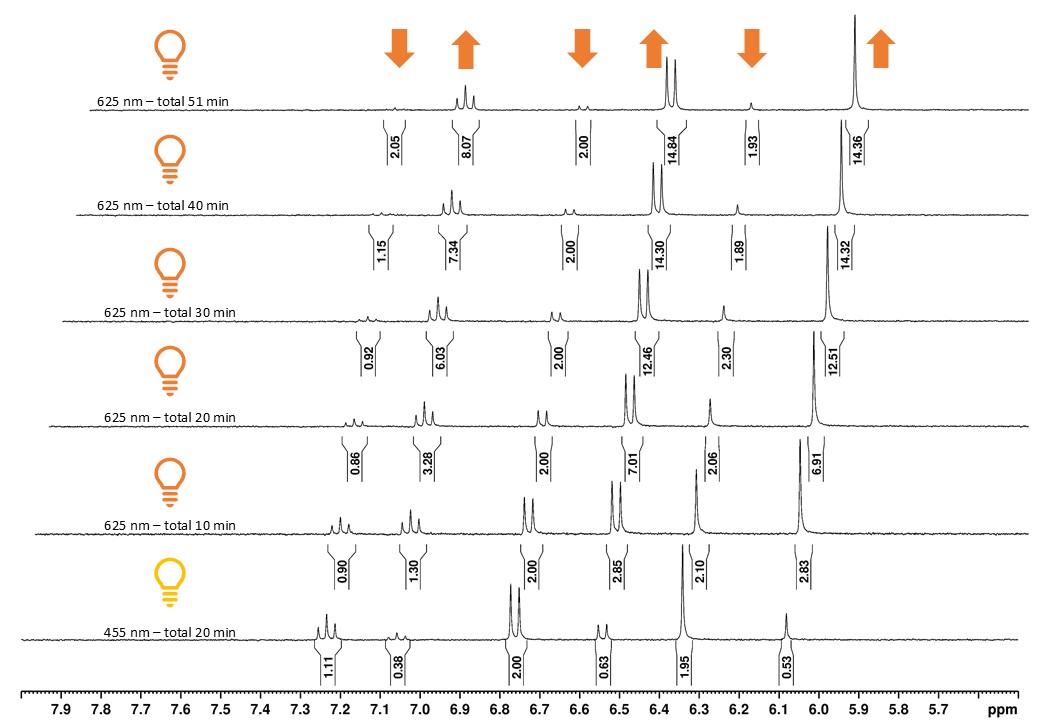


**Figure S27.** ^1^H NMR spectra (range 8.0-5.5ppm) of trans-AZO-N upon 625 nm LED irradiation in DMSO-d_6_ at room temperature.

**Table S2. Summary of the *%* of cis-AZO-N formed upon irradiation of *trans*-AZO-N with 625 nm LED.** The *cis*-**AZO-N** can be reached in around 88% after irradiation with 625 nm after a total irradiation time of 40 min. Even after a total of 51 min of irradiation time, the amount of *cis*-**AZO-N** in the PSS did not change further.

| **Time of irradiation (min)** | **% of *cis*-AZO-N in PSS (7.3-7.0ppm)** | **% of *cis*-AZO-N in PSS (6.8-6.5ppm)** | **% of *cis*-AZO-N in PSS (6.4-6.0ppm)** |
| --- | --- | --- | --- |
| *trans*-**AZO-N** after 455 nm irradiation | 25 | 24 | 21 |
| 10 | 59 | 59 | 57 |
| 20 | 79 | 78 | 77 |
| 30 | 87 | 86 | 85 |
| 40 | 87 | 88 | 88 |
| 51 | 87 | 88 | 88 |

**Irradiation of AZO-N (1 mM) in toluene-d_8_ with 455 and 340 nm LED irradiation**

First, the NMR sample was irradiated with 455 nm LED, for 2, 7, 15 and 45 mins. ^1^H NMR spectra were recorded. The relevant peaks of the Azo-N between 6.5-5.8 ppm, 4.0-3.0 ppm, and 1.75-0.5 ppm undergo a decrease/increase in intensity. It is to be noted that the Azo-N was already in a PSS state at the beginning of the irradiation after dissolving in the deuterated solvent. When irradiated after that in a second irradiation experiment with 340 nm for 2, 7, 15, and 20 mins. ^1^H NMR spectra were recorded. PSS was determined for both irradiation experiments. The peaks in the region of 3.5-3.0 ppm were used to calculate the PSS percentages. All other peaks seem to overlap with other peaks or were not unambiguously resolved.

**Irradiation with 455 nm LED from thermodynamically stable PSS**

**
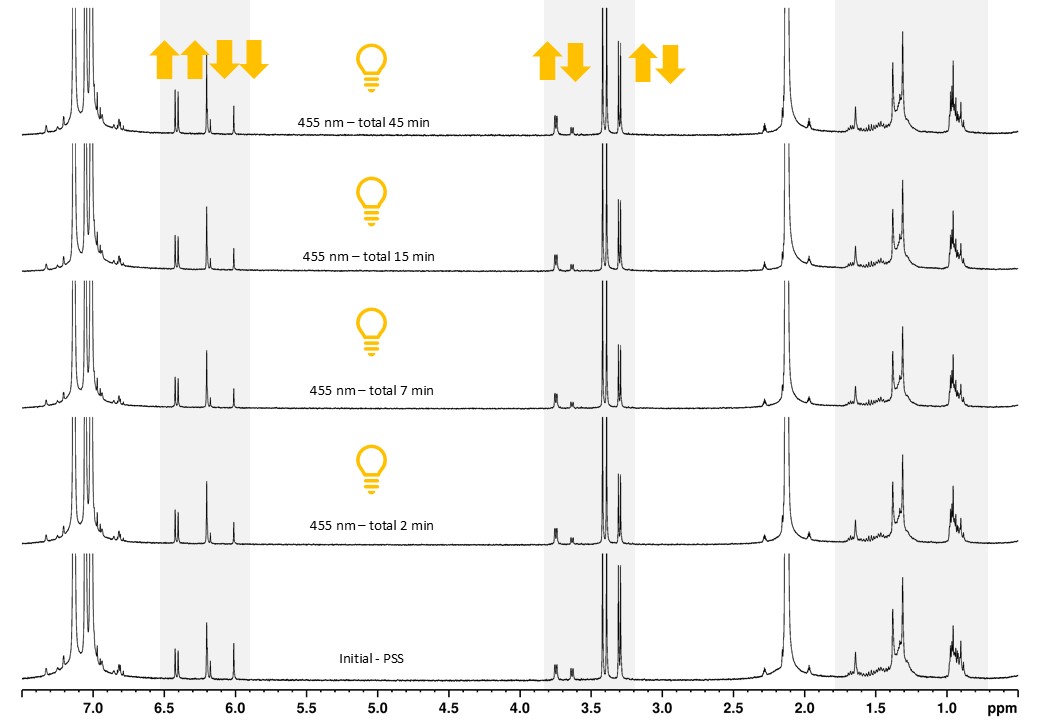
**

**Figure S28.** ^1^H NMR spectra of **AZO-N** (1 mM, thermodynamically stable state) upon irradiation with 455 nm LED in toluene-d_8_ at room temperature.


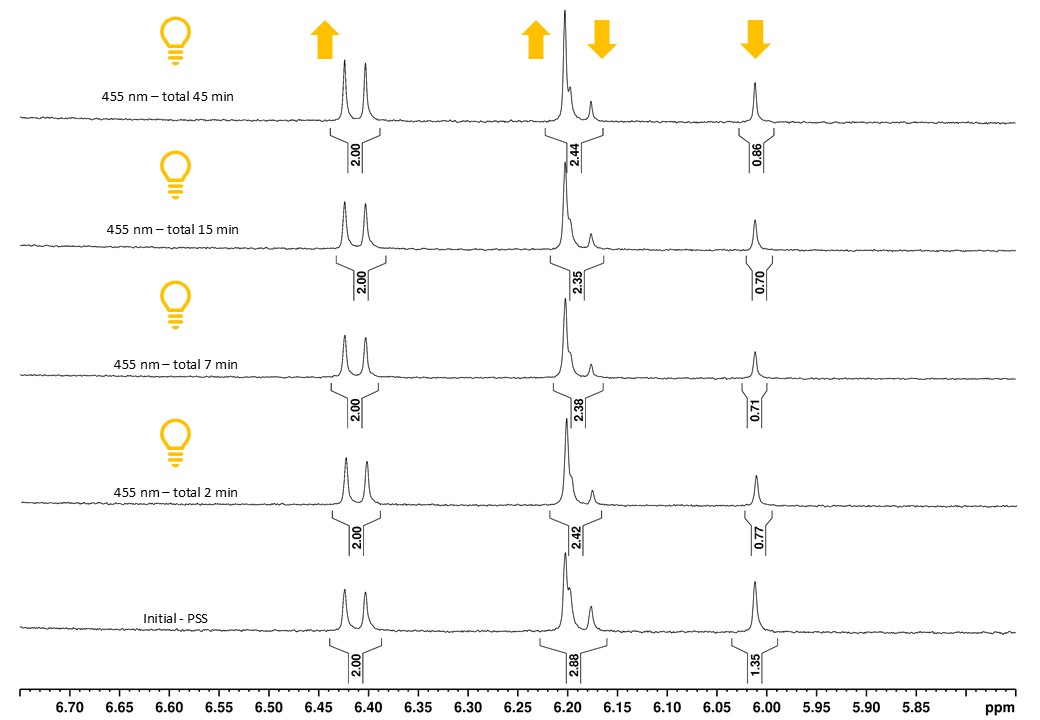


**Figure S29.** ^1^H NMR spectra **AZO-N** (1 mM, thermodynamically stable state) in the range 6.75-5.75 ppm upon 455 nm LED irradiation in toluene-d_8_ at room temperature.


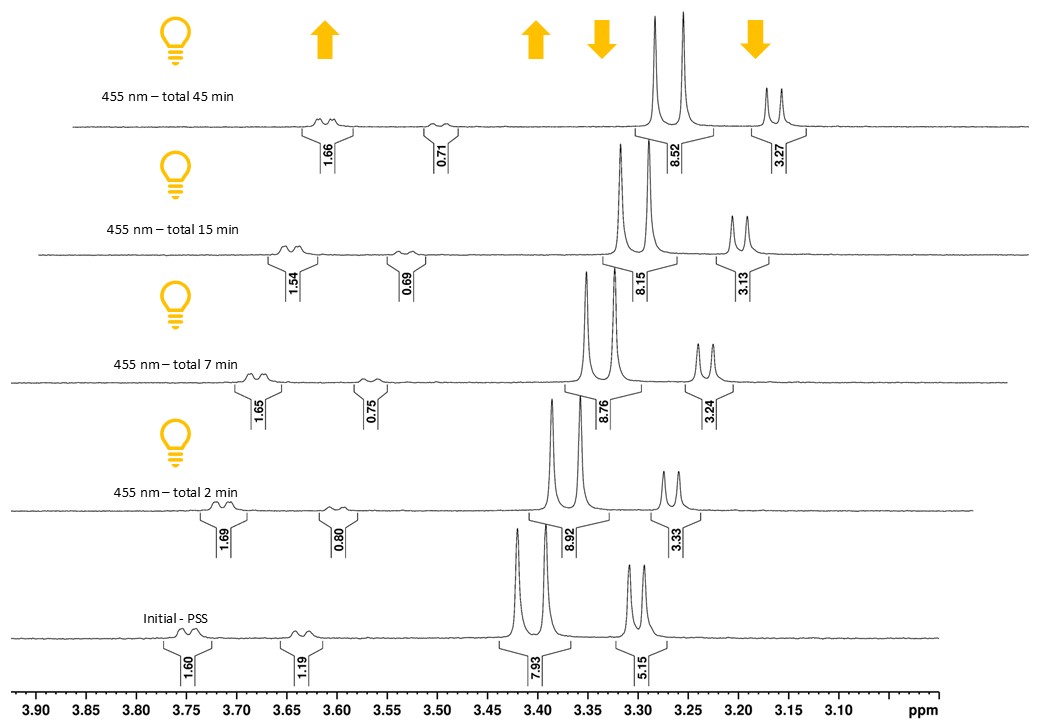


**Figure S30.** ^1^H NMR spectra (range 4.0-3.0 ppm) of 455 nm LED irradiation of Azo-N 1mM solution in toluene-d_8_ at room temperature. The *trans*-**AZO-N** can be reached in around 72% after irradiation with 455 nm after a total irradiation time of 15 min. Even after a total of 45 min of irradiation time, the amount of trans-AZO-N in the PSS did not change further.

**Table S3. Summary of the change in *%* trans-AZO-N upon irradiation of PSS with 455 nm LED.**

| **Time of irradiation (min)** | **% of *trans*-AZO-N in PSS (3.5-3.0ppm)** |
| --- | --- |
| Initial PSS | 60 |
| 2 | 73 |
| 7 | 73 |
| 15 | 72 |
| 45 | 72 |

**Irradiation of PSS with the highest amount of *trans*-AZO-N with 340 nm LED**


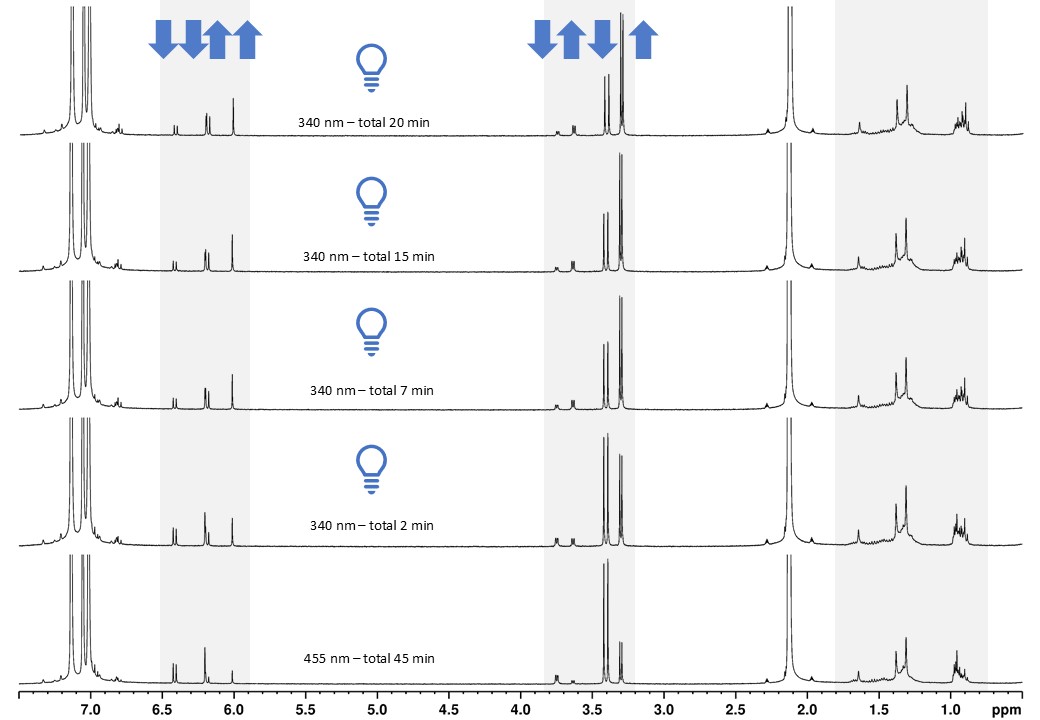


**Figure S31.** ^1^H NMR spectra of ***trans*-AZO-N** upon 340 nm LED irradiation in toluene-d_8_ at room temperature.


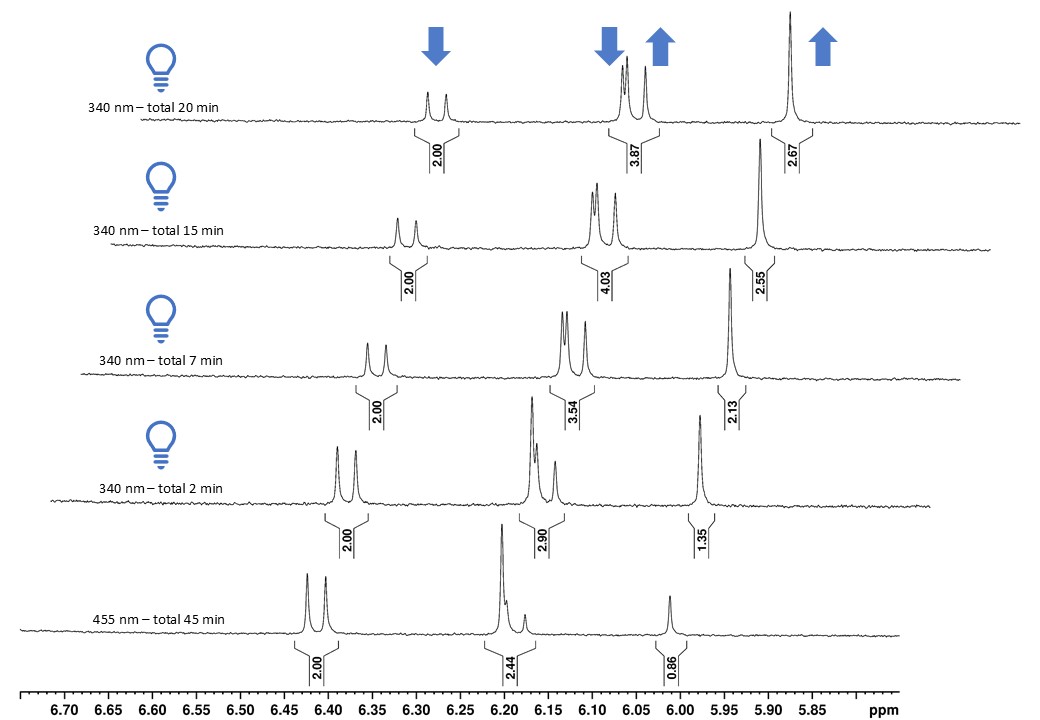


**Figure S32.** ^1^H NMR spectra of *trans*-AZO-N upon 340 nm LED irradiation in toluene-d8 at room temperature in the 6.7-5.7 ppm range.


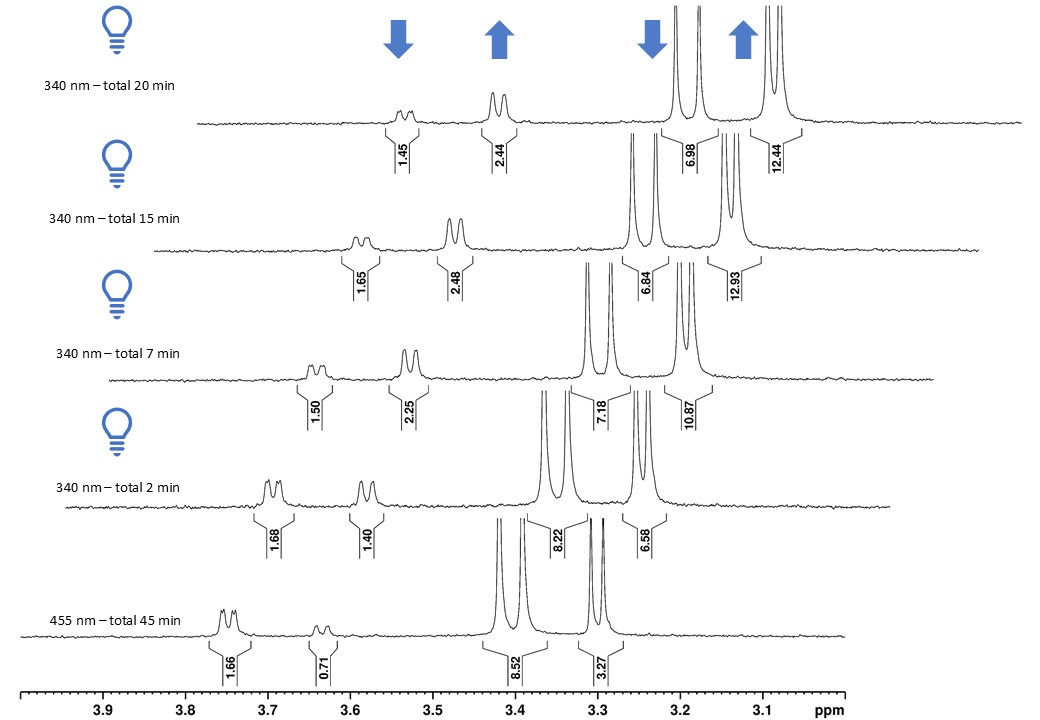


**Figure S33.** ^1^H NMR spectra of *trans*-AZO-N upon 340 nm LED irradiation in toluene-d8 at room temperature in the 3.5-3.0 ppm range.

**Table S4. Summary of the change in *%* trans-AZO-N upon irradiation of PSS with 340 nm LED.** The *cis*-**AZO-N** can be reached in around 65% after irradiation with 340 nm after a total irradiation time of 15 min. Even after a total of 20 min. of irradiation time, the amount of *cis*-**AZO-N** in the PSS did not change further.

| **Time of irradiation (min)** | **% of *cis*-AZO-N in PSS (3.5-3.0ppm)** |
| --- | --- |
| 45 (455 nm) | 28 |
| 2 | 45 |
| 7 | 59 |
| 15 | 65 |
| 20 | 64 |


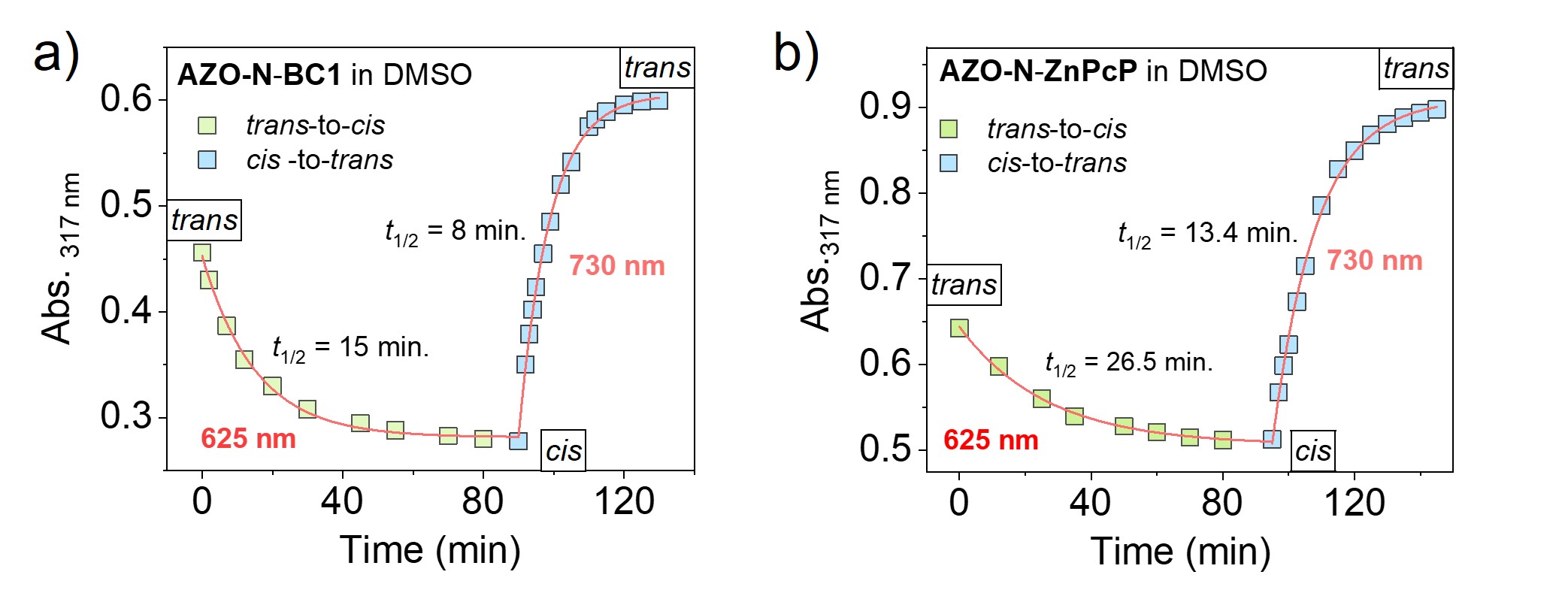


**Figure S34.** Photo-kinetics profiles of; a) **AZO-N-BC1**-DMSO solution and b) **AZO-N-ZnPcP**-DMSO solution, showing *trans-*to*-cis* photoswitching of **AZO-N** upon 625 nm (154 mW cm^-2^) or 730 nm (116 mW cm^-2^) light excitation.


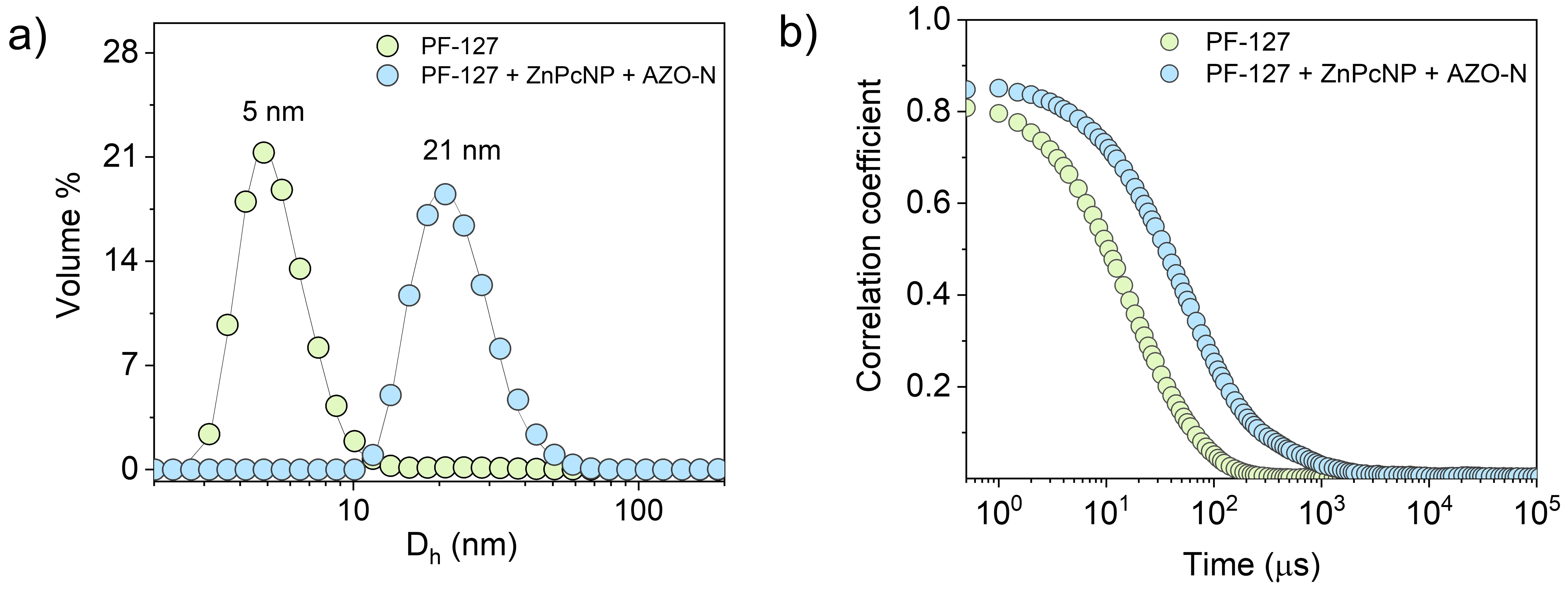


**Figure S35.** a) Dynamic light scattering profiles and b) corresponding autocorrelation function vs delay time profiles of PF-127 and PF-127+**ZnPcNP**+**AZO-N** in phosphate buffer solution at 25 ^o^C.


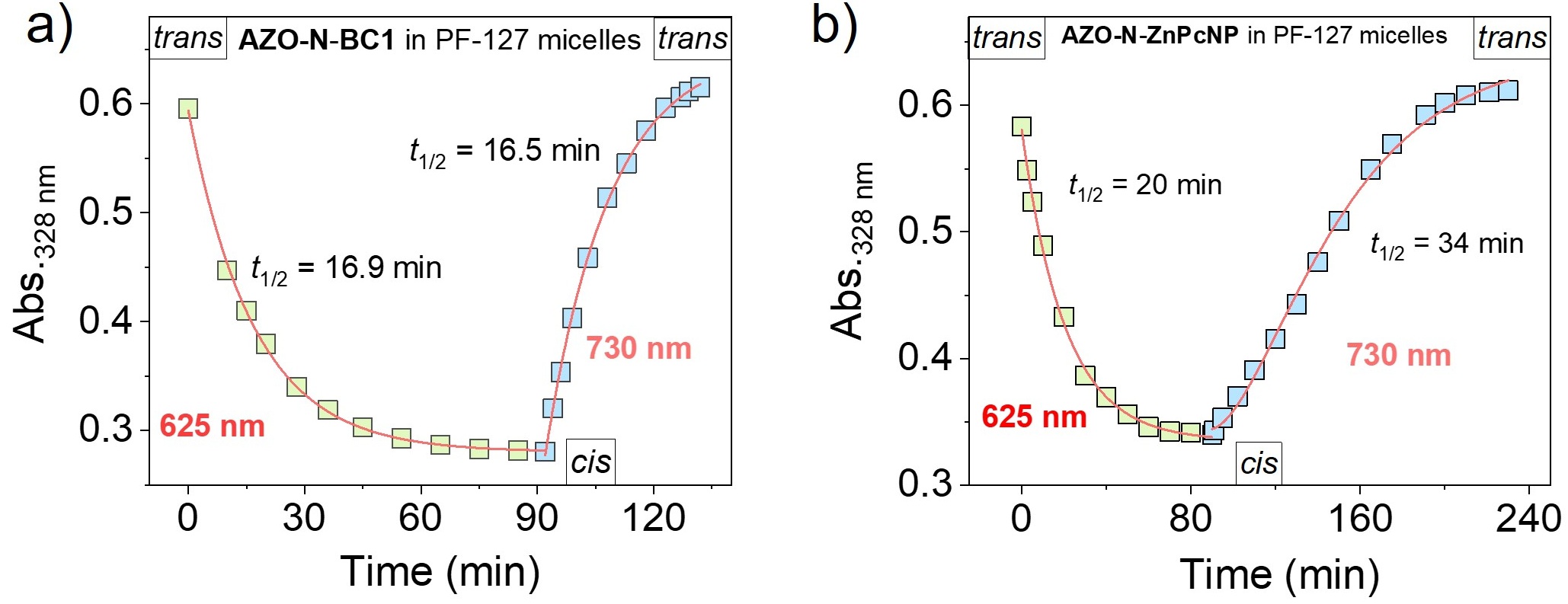


**Figure S36.** Photo-kinetics profiles of; a) **AZO-N-BC1**-PF-127 solution and b) **AZO-N-ZnPcNP**-PF-127 solution, showing *trans-*to*-cis* photoswitching of **AZO-N** upon 625 nm (154 mW cm^-2^) or 730 nm (116 mW cm^-2^) light excitation.


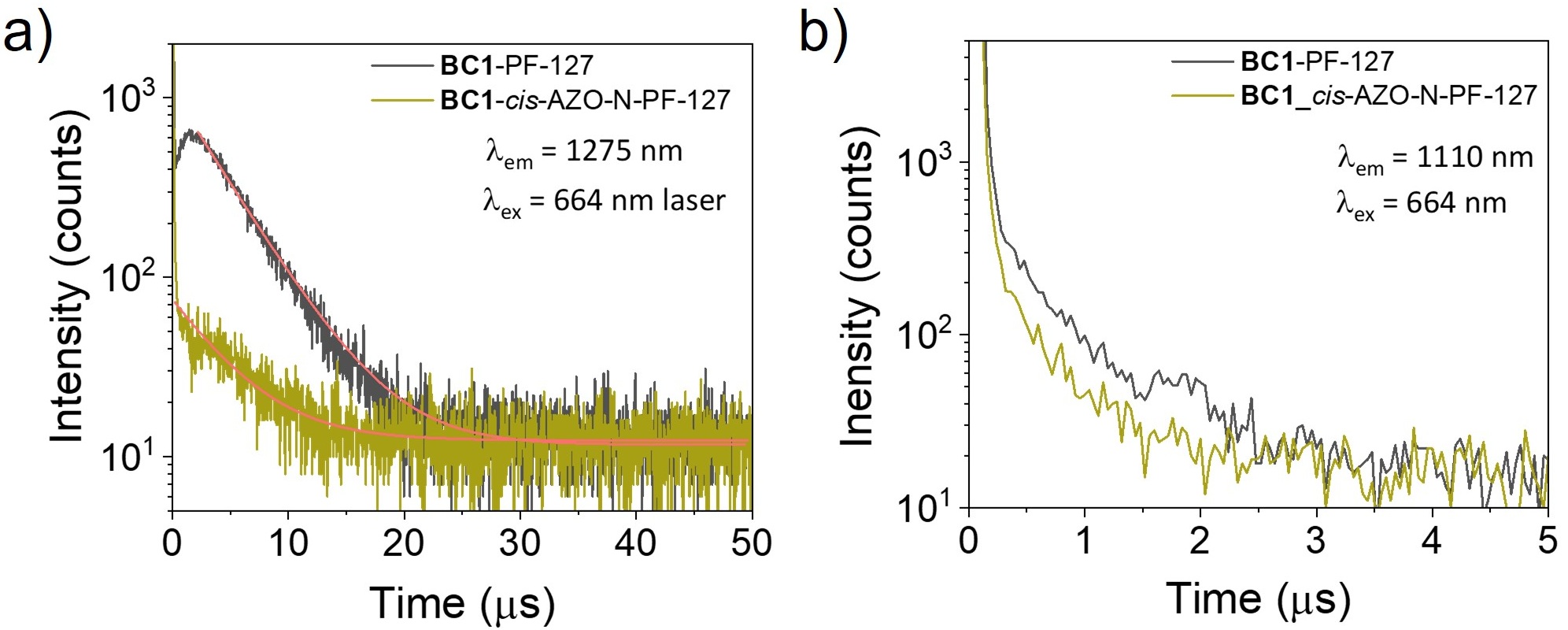


**Figure S37.** a) Time-resolved singlet oxygen phosphorescence of the system **BC1**-PF-127 measured at 1275 nm (λ_ex_ = 664 nm). The signal rises with lifetime 1.2 μs and decays with lifetime of 3.6 μs, which correspond to the triplet photosensitizer decay and singlet oxygen decay, respectively. b) Time resolved emission spectra of **BC1** in PF-127 micelles in the absence and presence of *cis*-AZO-N. λ_em_ = 1110 nm, λ_ex_= 664 nm laser.


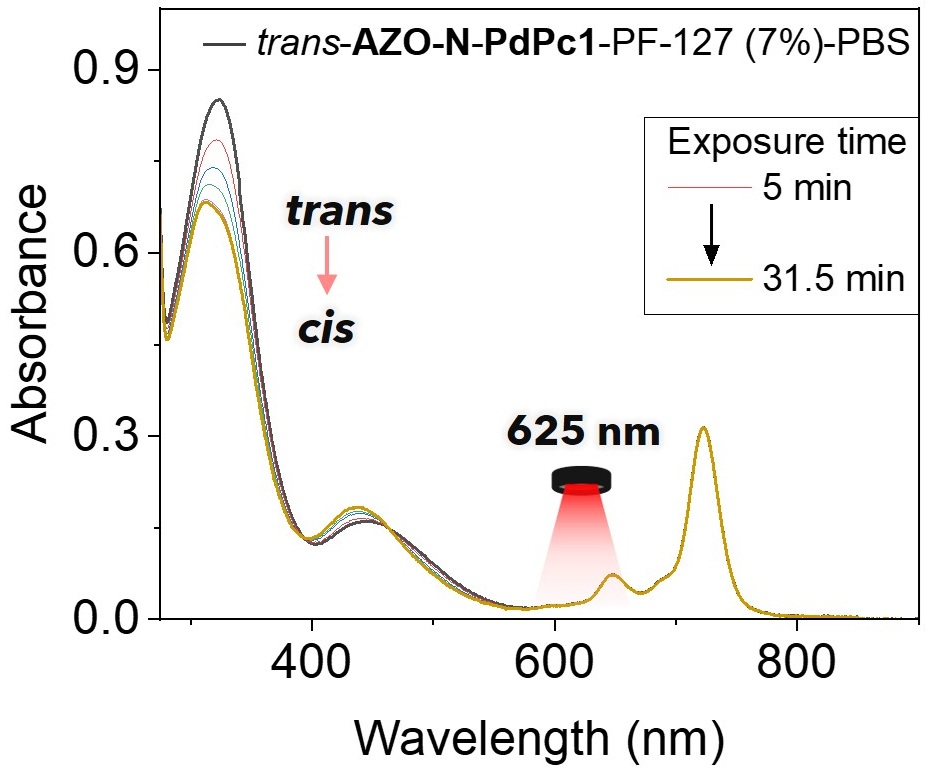


**Figure S38.** Absorption profiles of **AZO-N**-**PdPc1**-PF-127-PBS solution showing ***trans*** $\to$ ***cis*** photoswitching of AZO-N upon 625 nm (154 mW cm^-2^) LED excitation in air. **PdPc1** = 1.2 µM. **AZO-N** = 64.6 µM.

**Photoisomerization Quantum Yield Measurements in AZO-N-PdPc1-PF-127-PBS solution**

A PF-127 (7%) aqueous solution of **AZO-N** (50 µM), and **PdPc1** (1.5 µM) in 3mL phosphate buffer (10 mM, pH. 7.4) was prepared. The air-saturated solution was excited with either a 625 nm LED / 633 laser for *trans*-to-*cis* photoswitching or with a 730 nm LED for *cis*-to-*trans* photoswitching. The 1 cm pathlength quartz cuvette containing the sample solution was irradiated outside the automated sample holder to replicate the measurements performed for other samples, followed by the absorption spectral measurements using the automated sample holder. The photon flux was calculated using Power meter (PM100USB) with a S121C - Standard Photodiode Power Sensor (400-1100nm) and the software Thorlabs Optical Power Monitor.


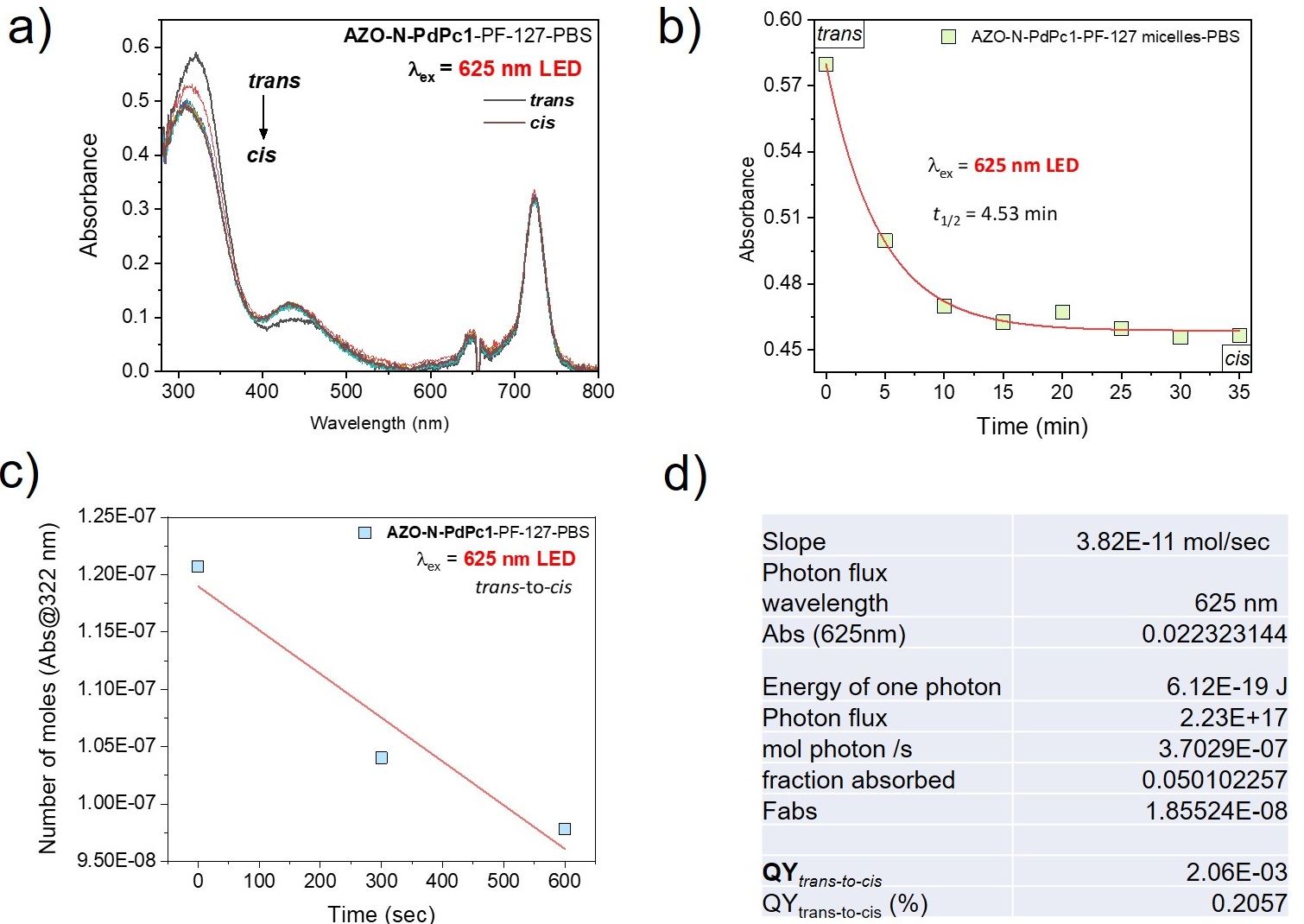


**Figure S39**. ***Trans-to-cis*, photoisomerization quantum yield measurement in air-saturated AZO-N-PdPc1-PF-127-PBS solution.** a) Plot showing change in absorption spectra upon 625 nm LED excitation. b) Kinetics profile for *trans-to-cis* photoswitching. c) Plot showing number of moles of **AZO-N** converting to *cis*-**AZO-N** upon irradiation with 625 nm LED with time. d) Tabulated parameters for the calculation of *trans*-to-*cis* photoisomerization.


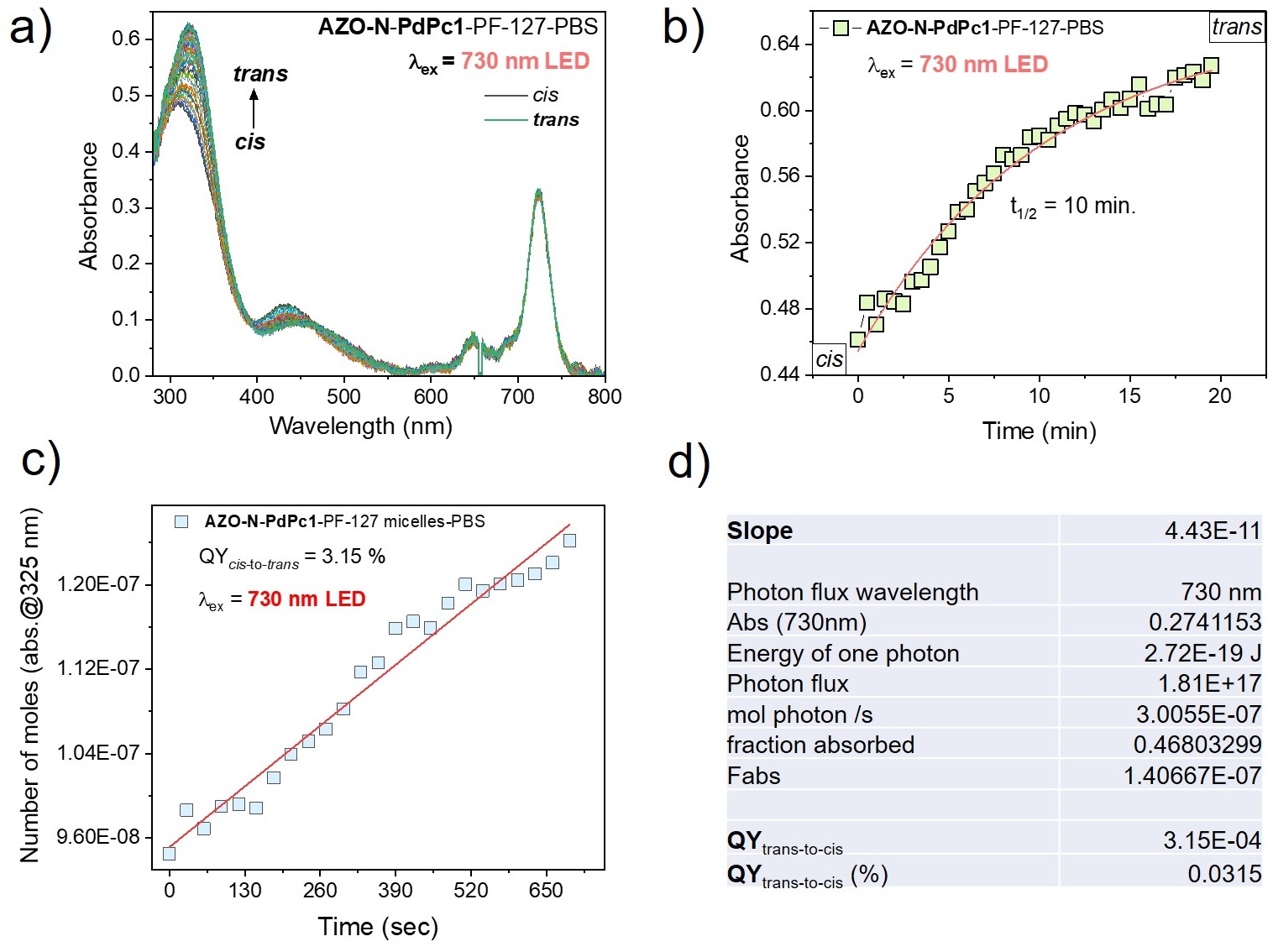


**Figure S40**. ***Cis-to-trans*, photoisomerization quantum yield measurement in the AZO-N-PdPc1-PF-127-PBS solution.** a) Plot showing change in absorption spectra upon 730 nm LED excitation. b) Kinetics profile for *cis-to-trans* photoswitching. c) Plot showing number of moles of **AZO-N** converting to *trans*-**AZO-N** upon irradiation with 730 nm LED with time. d) Tabulated parameters for the calculation of *cis*-to-*trans* photoisomerization.


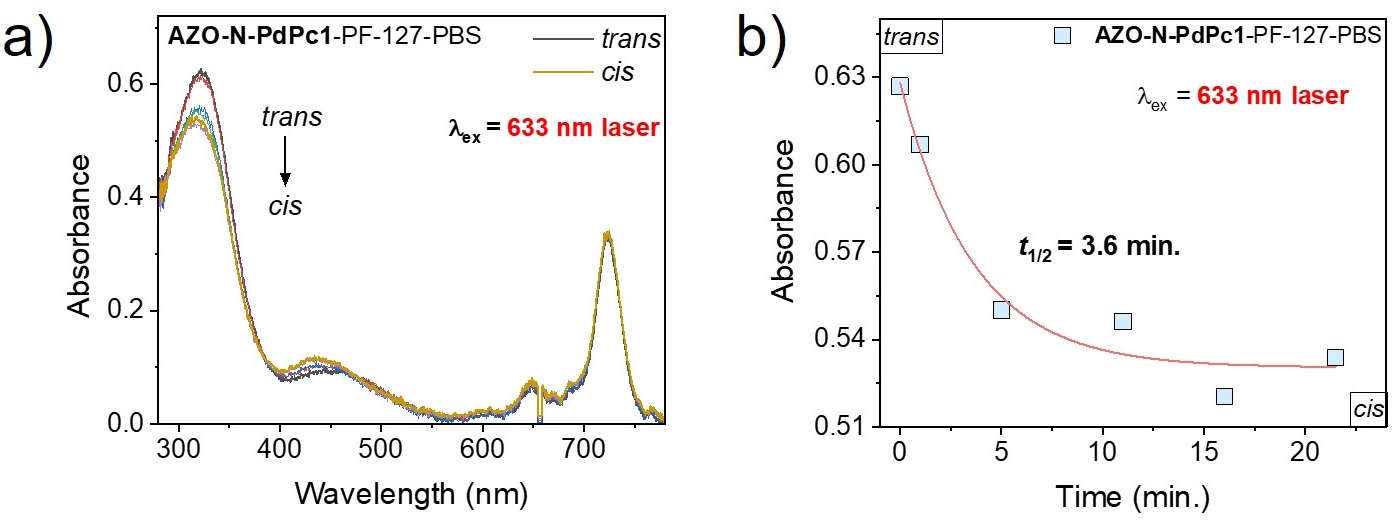


**Figure S41**. ***Trans-to-cis*, photoisomerization measurement in the AZO-N-PdPc1-PF-127-PBS solution with 633 nm laser excitation** a) Plot showing change in absorption spectra upon $633 \mp2 nm$Laser excitation (54 mW cm^-2^) b) Kinetics profile for *trans-to-cis* photoswitching upon laser excitation


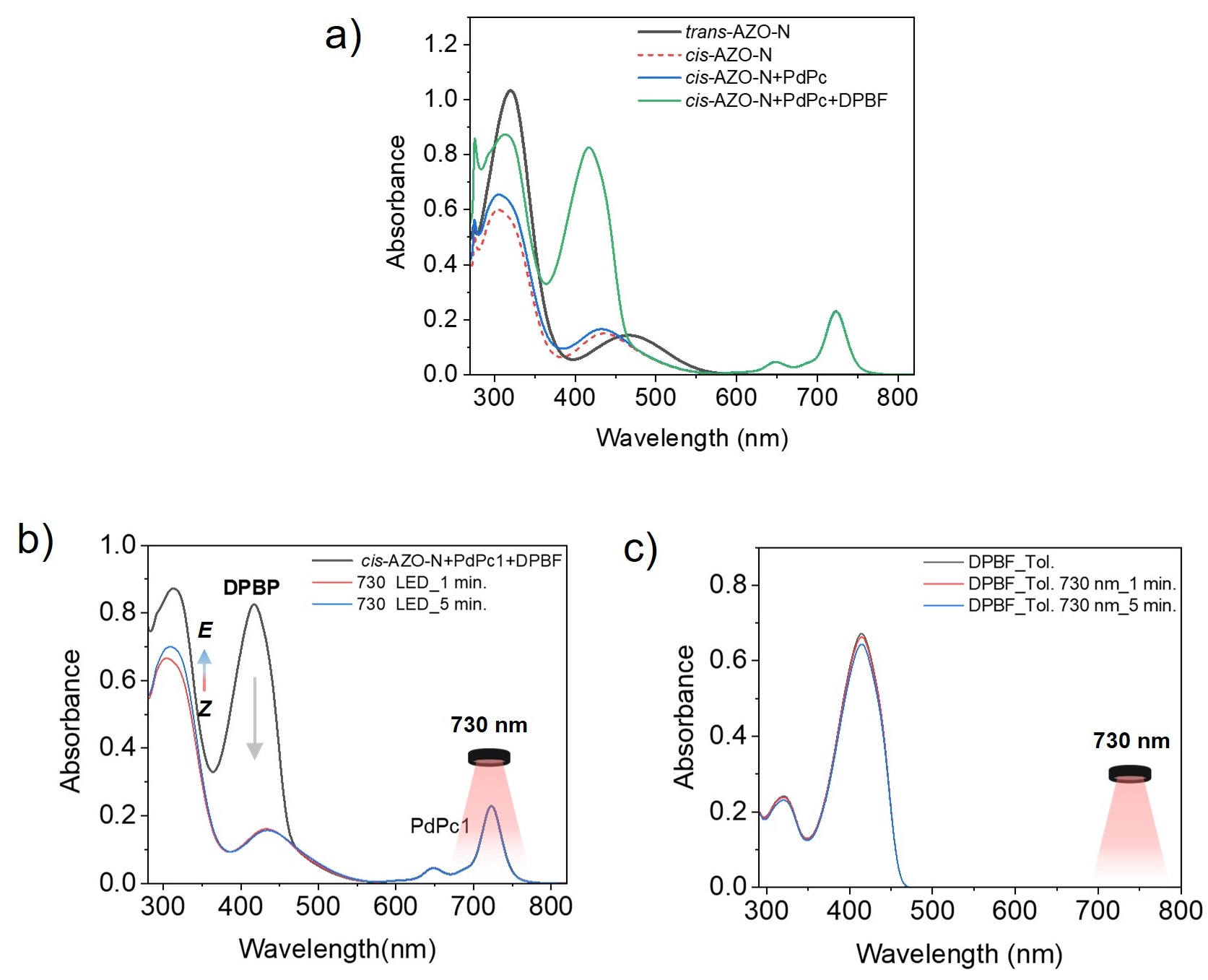


**Figure S42.** a) Absorption spectra of *trans* or *cis*-**AZO-N** in the presence of **PdPc1** and **DPBF** in toluene, b) Absorption profile showing decrease in the absorbance of DPBF in toluene in the presence of *cis*-**AZO-N** and **PdPc1** upon excitation with 730 nm LED. c) Absorption spectra of only DPBF in toluene upon 730 nm LED excitation. **PdPc1** = 1 µM. **AZO-N** = 36 µM. **DPBF** = 50 µM. 1 cm pathlength quartz cuvette.


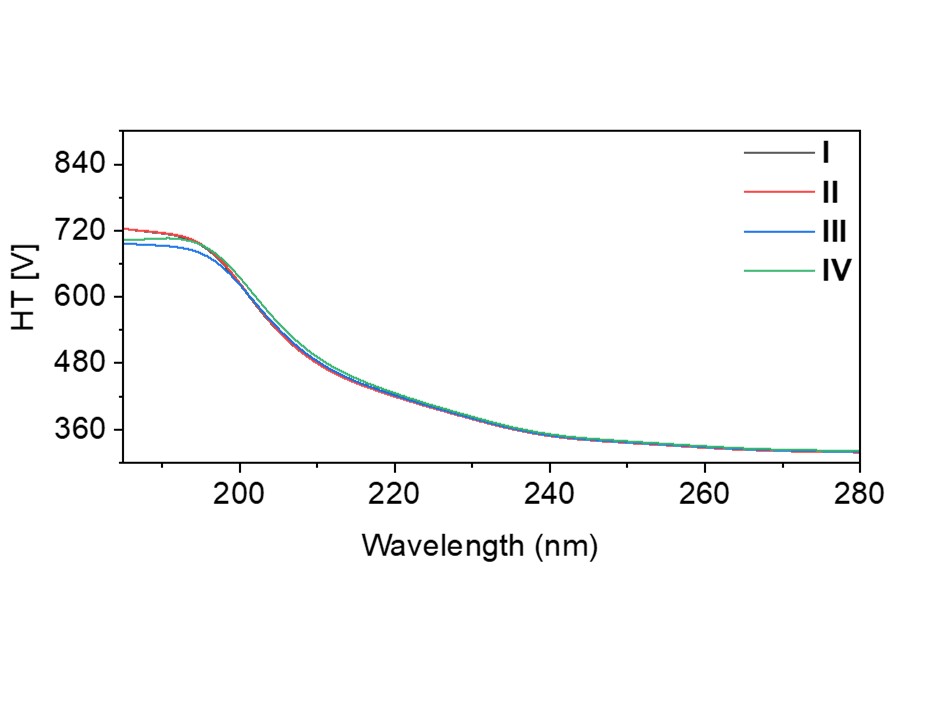


**Figure S43.** HT(V) *vs* wavelength profile of BSA in different solutions (I to IV). I → in PBS; II → PF-127 (2%)-PBS; III and IV → **AZO-N**-**PdPc1**-glutathione-PF-127-PBS, before irradiation (III), and after irradiation with 730 nm light (IV).

**4. Supporting References**

1. P. Bharmoria, S. Ghasemi, F. Edhborg, R. Losantos, Z. Wang, A. Mårtensson, M.-a. Morikawa, N. Kimizuka, Ü. İşci, F. Dumoulin, B. Albinsson, K. Moth-Poulsen, *Chem. Sci*., **2022**, *13*, 11904-11911.
2. M. Dubecký, R. Derian, L. Horváthová, M. Allan, and I. Štich, *Phys. Chem. Chem. Phys.*, **2011**, *13*, 20939-20945.
3. J. L. Elholm, P. Baronas, P. A. Gueben, V. Gneiting, H. Hölzel, K. Moth-Poulsen*,* *Digital Discovery*, **2025**, *4*, 2045-2051.
